# Supplementary material for: Fully lignocellulose-based PET analogues for the circular economy
Source: Nat Commun. 2022 Jun 13;13:3376. doi: 10.1038/s41467-022-30735-4 (PMC9192716; doi:10.1038/s41467-022-30735-4)
Supplement: Supplementary file 1 — Supplementary Information [file 41467_2022_30735_MOESM1_ESM.pdf]

Supplementary information

Fully lignocellulose-based PET analogues for the circular economy

Xianyuan Wu[1], Maxim V. Galkin[1], Tobias Stern[2], Zhuohua Sun*[3]Katalin Barta*[1,4]

[1]Stratingh Institute for Chemistry, University of Groningen, Groningen, The Netherlands.

[2]University of Graz, Institute of Systems Sciences, Innovation and Sustainability Research, Merangasse 18/I, 8010, Graz, Austria

[3] Beijing Key Laboratory of Lignocellulosic Chemistry, Beijing Forestry University, No. 35 Tsinghua East Road Haidian District, Beijing, 100083, P. R. China.

[4]Department of Chemistry, Organic and Bioorganic Chemistry, University of Graz, Heinrichstrasse 28/II, 8010 Graz, Austria

*Correspondence to:katalin.barta@uni-graz.at, sunzhuohua@bjfu.edu.cn

Table of Contents

Supplementary Methods **1**

1.1 Preparation of Cu20-PMO catalyst 3

1.2 Preparation of model compounds 1G, 1S, PC, PC_{cis}, PC_{trans} and DMFD 3

1.3 Conversion, selectivity and yield calculation 5

1.4 Spectral data of the model compounds 6

1.5 General experimental procedures 18

Supplementary Note 1. A comprehensive strategy for the production of PC, DMTA, DMFD, ethanol, furfural and ethylene glycol entirely from lignocellulose **20**

Supplementary Note 2. Catalytic defunctionalization of model compound 1G to PC **24**

2.1 Establishing of reaction conditions (catalysts, reaction temperature, solvents and pressure) for demethoxylation and hydrogenation of 1G 24

2.2 Proposed reaction network for defunctionalization of 1G 25

2.3 Reaction kinetics for catalytic defunctionalization of 1G, 1H, **1** and PC 26

2.4 Kinetic modeling of 1G defunctionalization and hydrogenation 27

2.5 Fractionation, catalytic funneling and analysis of the crude mixture obtained from RCF of beech lignocellulose 27

2.6 Upscaling the synthesis procedure of 1G from pine, 1G/1S from poplar and beech wood 32

Supplementary Note 3. Synthesis of poly (PC/TPA) and poly (PC/FDCA) using model compound PC and its isomers **33**

3.1 Synthesis, analysis and characterization of poly (PC/TPA) 33

3.1.1 DSC, TGA and GPC of poly (PC_{cis}/TPA), poly (PC_{trans}/ TPA) and poly (PC/TPA) 42

3.2 Synthesis, analysis and characterization of poly (PC/FDCA) 45

3.2.1 DSC, TGA and GPC of poly (PC/FDCA) 48

3.3 The FTIR spectra of poly (PC/TPA) and poly (PC/FDCA) 51

3.4 Copolymerization with TPA and FDCA using crude aliphatic alcohols mixture from defunctionalization of the model 1G/1S mixture 52

3.4.1 DSC, TGA and GPC of poly (PC/**1**/TPA) and poly (PC/**1**/FDCA) 54

3.5 A comprehensive biorefinery strategy for the production of gasoline, PET analogues and jet fuels from beech wood (10 g) 55

3.5.1 Fraction A: analysis, characterization, and hydrodeoxygenation 57

3.5.2 Fraction B: analysis, characterization, and polymerization 57

3.5.3 Fraction C: analysis, characterization, and hydrodeoxygenation 59

3.6 Proposed catalytic downstream conversion pathways 62

Supplementary Note 4. Recycling studies 63

4.1 Establishing of reaction conditions for alcoholysis of poly (PC/TPA) 63

4.2 A circular economy for production, recycling, reproduction of poly (PC/TPA) 63

Supplementary Note 5. Considerations for the catalytic activity of Raney Ni catalyst in the demethoxylation /hydrogenation of 1G 68

Supplementary Note 6. Determination of the lignin utilization efficiency based on a per carbon basis 69

Supplementary Note 7. Establishment of pathway toward separation and conversion of carbohydrate to FDCA 70

Supplementary Note 8. Preliminary techno-economic analysis (TEA) 72

Supplementary Note 9. Spectral data of isolated compounds 76

Supplementary Note 10. DynaFit script and input data 78

Supplementary references 80

Supplementary Methods

Column chromatography: was performed using Merck silica gel type 9385 230–400 mesh and typically dichloromethane and methanol or EtOAc and pentane as eluent.

Thin layer chromatography (TLC): Merck silica gel 60, 0.25 mm. The components were visualized by UV or $KMnO_4$ staining.

Gas Chromatography (GC) was used for products identification as well as determination of conversion and selectivity values.

At the University of Groningen (RUG): Products identification was performed by GC-MS (Shimadzu QP2010 Ultra) equipped with an HP-1MS column, and helium as carrier gas.

GC-MS analysis method (RUG): The temperature program started at 50 °C for 5 min, heated by 10 °C·min^{-1} to 320 °C and held for 5 min. Conversion and products selectivity were determined by GC-FID (Shimadzu GC-2014) equipped with an HP-5MS column using nitrogen as carrier gas.

GC-FID analysis method (RUG): The temperature program started at 50 °C for 5 min, heated by 10 °C·min^{-1} to 320 °C and held for 5 min.

At the University of Graz (UniGraz): Products identification was performed by GC-MS (5975C MSD) equipped with an HP-5 MS column, and helium as carrier gas.

GC-MS method (UniGraz): The temperature program started at 50 °C for 5 min, heated by 10 °C·min^{-1} to 325 °C and held for 5 min. Conversion and products selectivity were determined by GC-FID (Agilent 8890 GC) equipped with an HP-5MS column using nitrogen as carrier gas.

GC-FID analysis method (UniGraz): The temperature program started at 50 °C for 5 min, heated by 10 °C·min^{-1} to 325 °C and held for 5 min.

Nuclear Magnetic Resonance (NMR) spectroscopy:

University of Groningen: ^{1}H, ^{13}C NMR and 2D NMR spectra were recorded on a Varian Mercury Plus 400, Agilent MR 400 (400 and 101 MHz, respectively) and a Bruker Avance NEO 600 (600 and 151 MHz, respectively);

University of Graz: ^{1}H, and ^{13}C NMR spectra were recorded on a Bruker Avance III 300 MHz (300 and 75 MHz, respectively) and 2D NMR spectra were recorded on a Bruker Avance III 700 MHz with Cryoplatform and a 5mm Triple-Resonance cryoprobe (700 and 175 MHz, respectively).

^{1}H,^{13}C NMR and 2D NMR spectra were recorded at RT. Chemical shift values are reported in ppm with the solvent resonance as the internal standard ($CDCl_3$: 7.26 for ^{1}H, 77.0 for ^{13}C; CD_3OD: 3.31 for ^{1}H, 49.0 for ^{13}C; DMSO-d_6: 2.50 for ^{1}H, 39.5 for ^{13}C). Data are reported as follows: chemical shifts, multiplicity (s = singlet, d = doublet, t = triplet, q = quartet, br. = broad, m = multiplet), coupling constants (Hz), and integration.

Gel Permeation Chromatography (GPC): GPC was performed at the University of Graz on a SHI-MADZU NEXERA equipped 2×SDV analytical Linear M (8×300 mm, 5µm) plus 1×precolumn SVD (8×50mm, 5µm). The columns were operated at 40 °C with a flow-rate of 1 mL·min^{-1} of THF. Detection

was accomplished at 40 °C using an SPD-M40 photoarray detector in series. The molecular weight determination were performed using polystyrene standards of known molecular weight distribution.

Differential Scanning Calorimetry (DSC) was conducted at the Graz University of Technology on a Perkin Elmer DSC 8500. In a typical procedure, the sample (5-10 mg) was weighed into a DSC aluminium pan and then capped with a lid. The sample was sealed and heated from 25 to 250 °C with a heating rate of 10 $°C·min^{-1}$. Then, it was cooled to 25 °C with a heating rate of 10 $°C·min^{-1}$. Subsequently, a second heating scan to 250 °C with the same heating rate was performed. All of the experiments were performed under N_2 flow with a flow rate of 20 $mL·min^{-1}$.

Thermogravimetric analysis (TGA) was performed at the Graz University of Technology on a Netzsch Jupiter STA 449C thermogravimetric analyzer. Typically, the sample (1-3 mg) was weighed into a platinum pan. The sample was heated from 20 to 550 °C with a heating rate of 10 $°C·min^{-1}$ under N_2 flow with a flow rate of 20 $mL·min^{-1}$. The temperatures were recorded when 5 % weight loss ($T_{5\%}$) and 90% weight loss rate ($T_{90\,\%}$) occurred.

Inductively coupled plasma mass spectrometry (ICP-MS) was performed at University of Graz on a Agilent 7900 ICP-MS. Typically, the samples were solubilized with 5 mL HNO_3 in the MLS ultraclave and then heated to 250 °C for 30 mins before analysis by ICP-MS.

Abbreviations

1G: dihydroconiferyl alcohol / 4-(3-hydroxypropyl)-2-methoxyphenol
1S: dihydrosinapyl alcohol / 4-(3-hydroxypropyl)-2,6-dimethoxyphenol
1H: 3-(4-hydroxyphenyl)-1-propanol
1: 4-(3-hydroxypropyl)-2-methoxycyclohexanol
2H: 4-propylphenol
2G: 2-methoxy-4-propylphenol
2: 4-ethyl cyclohexanol
2S: 2,6-dimethoxy-4-propylphenol
3G: 4-ethylguaiacol
3S: 4-ethyl-2,6-dimethoxyphenol
3: 4-propyl cyclohexanol
3H: 4-ethylphenol
DMFD: dimethyl 2,5-furandicarboxylate
TPA: terephthalic acid
DMTA: dimethyl terephthalate
TBT: titanium (IV) butoxide
FDCA: 2,5-furandicarboxylic acid
HDO: hydrodeoxygenation
PC: 4-(3-hydroxypropyl)cyclohexanol
PET: polyethylene terephthalate
PEG polyethylene glycol
RCF: reductive catalytic fractionation

1.1 Preparation of Cu20-PMO catalyst

The Cu20-PMO catalyst was prepared according to our previously reported procedure.[1] In a typical procedure, a solution containing $AlCl_3 \cdot 6H_2O$ (12.07 g, 0.05 mol), $Cu(NO_3)_2 \cdot 2.5H_2O$ (6.98 g, 0.03 mol) and $MgCl_2 \cdot 6H_2O$ (24.4 g, 0.12 mol) in deionized water (200 mL) was dropwise added to a solution containing Na_2CO_3 (5.30 g, 0.05 mol) in water (300 mL) at 60 °C under vigorous stirring. The pH value was always kept between 9 and 10 by addition of small portions of a 1 M solution of NaOH. The mixture was vigorously stirred at 60 °C for 72 h. After cooling to room temperature, the light blue solid was filtered and resuspended in a 2 M solution of Na_2CO_3 (300 mL) and stirred overnight at 40 °C. The catalyst precursor was filtered and washed with deionized water until chloride free. After drying the solid for 6 h at 100 °C followed by the calcination at 460 °C for 24 h in air, 9.5 g of Cu20-PMO was obtained.

1.2 Preparation of model compounds 1G, 1S, PC, PC_{cis}, PC_{trans} and DMFD

Preparation of model compound 1G: 1G was synthesized according to previously developed procedure with slight modifications.[2, 3] Typically, a 100 mL high pressure Parr autoclave was charged with 5 % Pd/C (0.1 g), 40 mL methanol and trans-ferulic acid (5 g, 25.74 mmol). The reactor was sealed, purged 3 times with H_2 and then pressurized with H_2 (40 bar) and stirred at room temperature overnight. After filtering through a Celite plug, the solvent was evaporated under reduced pressure to provide 5.02 g of 3-(4-hydroxy-3-methoxyphenyl)propanoic acid (25.61 mmol, 99.5 % yield), which was used without further purification. To a rapidly stirred suspension of $LiAlH_4$ (1.45 g, 38.26 mmol) in 50 mL of THF was dropwise added a solution of 3-(4-hydroxy-3-methoxyphenyl)propanoic acid (5 g, 25.51 mmol) in 20 mL of THF at 0 °C. After the addition was completed, the reaction mixture was allowed to warm to RT and stirred overnight. The reaction mixture was quenched by ice water, neutralized by 1M HCl (10 mL) and then filtrated to remove the white solid. The remaining solution was extracted with EtOAc (3×50 mL). The combined organic extract was washed with saturated solution of $NaHCO_3$ (50 mL) and brine (100 mL), dried over anhydrous $MgSO_4$ and the solvent was evaporated under reduced pressure to provide 3.2 g of dihydroconiferyl alcohol (1G) (17.58 mmol, 68.9% yield).

Preparation of model compound 1S: 1S was synthesized according to previously developed procedure with slight modifications.[2, 3] Typically, a 100 mL high pressure Parr autoclave was charged with 5 % Pd/C (0.1 g), 40 mL methanol, 3-(4-hydroxy-3,5-dimethoxyphenyl) acrylic acid (22.32 mmol, 5 g). The reactor was sealed, purged 3 times with H_2 and then pressurized with H_2 (40 bar) and stirred at RT overnight. After filtering through a Celite plug, the solvent was evaporated under reduced pressure to provide 5.0 g of 3-(4-hydroxy-3,5-dimethoxyphenyl)propanoic acid (22.1 mmol, 99% yield). To a rapidly stirred suspension of $LiAlH_4$ (1.26 g, 38.18 mmol) in 50 mL of THF was dropwise added a solution of 3-(4-hydroxy-3-methoxyphenyl)propanoic acid (5 g, 25.51 mmol) in 20 mL of THF at 0 °C. After the addition was completed, the reaction mixture was allowed to warm to RT and stirred overnight. The reaction mixture was quenched by ice water, neutralized by 1M HCl and then filtrated to remove

the white solid. The remaining solution was extracted by EtOAc (3×50 mL). The combined organic extract was washed with a saturated solution of $NaHCO_3$ (50 mL), brine (50 mL), dried over anhydrous $MgSO_4$; the solvent was evaporated under reduced pressure to provide 3.42 g of dihydrosinapyl alcohol (1S) (16.13 mmol, 63.2% yield).

Preparation of model compounds PC_{cis} and PC_{trans}:The synthesis of PC_{cis} and PC_{trans} was performed in three steps according to a reported procedure with slight modifications.[4]

Step 1: Synthesis of para-coumaric acid ethyl ester. 5 g of para-coumaric acid (30.47 mmol) was dissolved in 60 mL of ethanol in round bottom flask under continuous stirring. Then 1 mL of concentrated hydrochloric acid (37-38 wt %) was added and refluxed at 95 °C overnight. After cooling to RT, ethanol was removed under reduced pressure. The obtained oil was dissolved in 100 mL of EtOAc and washed with saturated $NaHCO_3$ solution (50 mL), brine (2×50 mL) and dried over anhydrous $MgSO_4$. Para-coumaric acid ethyl ester was obtained in 95 % yield (28.94 mmol, 5.55 g).

Step 2: Synthesis of 3-(4-hydroxycyclohexyl)propionic acid ethyl ester (cis and trans isomers). The autoclave was charged with 2 g Raney Ni catalyst, 5 g para-coumaric acid ethyl ester (26.04 mmol), and 20 mL isopropanol. The reactor was sealed and pressurized with H_2 (40 bar) at RT. The reactor was heated to 160 °C and stirred at 400 rpm overnight. After completion of the reaction, the reactor was cooled to RT. The products 3-(4-hydroxycyclohexyl)propionic acid ethyl ester containing cis-isomer and trans-isomer with the ratio of 1:1 were isolated by silica gel column chromatography (gradient elution: Pentane : EtOAc 90:10 to 40:10) in 93 % yield (24.24 mmol, 4.8 g, cis:trans 1:1).

Step 3: Synthesis of 4-(3-hydroxypropyl)cyclohexanol (PC) diol (cis and trans isomers). For synthesis of PC (cis and trans), the same procedure as specified below was used. To a rapidly stirring suspension containing 30 mL THF and 0.4 g $LiAlH_4$ as reducing agent, a solution of 3-(4-hydroxy-3-methoxyphenyl)propanoic acid (cis or trans, 7.57 mmol, 1.5 g) in 20 mL THF was dropwise added in a three-neck flask that was cooled in an ice-bath. After the addition was completed, the reaction mixture was allowed to warm to RT and stirred overnight. After completion of the reaction, the mixture was poured onto ice water to reduce the excess of $LiAlH_4$, and 20 mL solution containing 1M HCl (10 mL) was added. The precipitate was removed by filtration. The separated solution containing THF, water and the product was concentrated: THF was removed under reduced pressure and the remaining solution was extracted with dichloromethane (4×50 mL). The combined organic extract was washed with saturated $NaHCO_3$ solution (2×50 mL) and brine (2×50 mL), dried over anhydrous $MgSO_4$ and the solvent was evaporated under reduced pressure to provide 0.75 g (4.74 mmol) and 0.81 g (5.12 mmol) of 4-(3-hydroxypropyl)cyclohexanol (cis and trans) as a white solid, respectively (cis: 62.6 % yield, trans: 67.6 % yield).

Preparation of model compound DMFD: The synthesis of dimethyl 2,5-furamdicarboxylate (DMFD) was carried out according to previously reported procedure.[5] Typically, a 100 mL round bottom flask, equipped with a magnetic stirring bar and reflux condenser, was charged with 4 g of 2,5-furandicarboxylic acid (25.64 mmol) (FDCA), 50 mL methanol, and 1 mL 12 M HCl. The mixture was refluxed at

80 °C for 18 h. After completion of the reaction, the pH value of the reaction mixture was adjusted to 7 by adding appropriate amount of 1 M Na_2CO_3 solution. The obtained solution was concentrated and then extracted with dichloromethane (4×30 mL). The combined organic extract was dried over anhydrous $MgSO_4$ and the solvent was evaporated under reduced pressure to provide 3.82 g of dimethyl 2,5-furandicarboxylate (DMFD) (20.76 mmol) as a white solid in 81 % yield.

1.3 Conversion, selectivity and yield calculation

i. For catalytic demethoxylation/hydrogenation of 1G to PC:

$$\text{Conversion (\%)} = \frac{\text{Mol of (original 1G} - \text{remaining 1G)}}{\text{mol oforiginal 1G}} \times 100\ \% \quad \text{Eq 1}$$

$$\text{Selectivity (\%)} = \frac{\text{Mol of the obtained PC}}{\text{Mol of (original 1G} - \text{remaining 1G)}} \times 100\ \% \quad \text{Eq 2}$$

$$\text{Yield (\%)} = \text{Conversion (\%)} \times \text{Selectivity (\%)} \quad \text{Eq 3}$$

ii. For copolymerization of PC with DMTA and DMFD:

$$\text{Yield (\%)} = \frac{\text{Mass of the obtained poly (PC/ TPA/FDCA)}}{\text{Mass of theoretically obtained poly(PC, TPA/FDCA)}} \times 100\ \% \quad \text{Eq 4}$$

iii. For methanolysis depolymerization of poly (PC/TPA):

$$\text{Conversion (\%)} = \frac{\text{Mass of}\left(\text{original poly (PC/TPA)} - \text{remaining poly (PC/TPA)}\right)}{\text{Mass of initial poly (PC/TPA)}} \quad \text{Eq 5}$$

$$\text{Monomers yield (\%)} = \frac{\text{Mass of the obtained monomers}}{\text{Theoretical mass of monomers from poly (PC/TPA)}} \times 100\ \% \quad \text{Eq 6}$$

iv. For calculation of yield to hydrocarbon alkanes

The quantification of hydrocarbon alkanes was performed using the response of the flame-ionization detector (FID) and the response factor was estimated by Effective Carbon Number (ECN) method.[6]

$$\text{F (R} - \text{wt)} = \frac{\text{Mw of products} \times \text{ECN of dodecane}}{\text{Mw of dodecane} \times \text{ECN of product}} \quad \text{Eq 7}$$

ECN of dodecane = 12 (Carbon number)

$$\text{ECN of products} = \text{Carbon number of hydrocarbons} \quad \text{Eq 8}$$

$$\text{F (R} - \text{wt)} = \frac{\text{Peak area counts for dodecane} \times \text{wt of products}}{\text{Peak area counts for products} \times \text{wt of dodecane}} \quad \text{Eq 9}$$

1.4 Spectral data of the model compounds

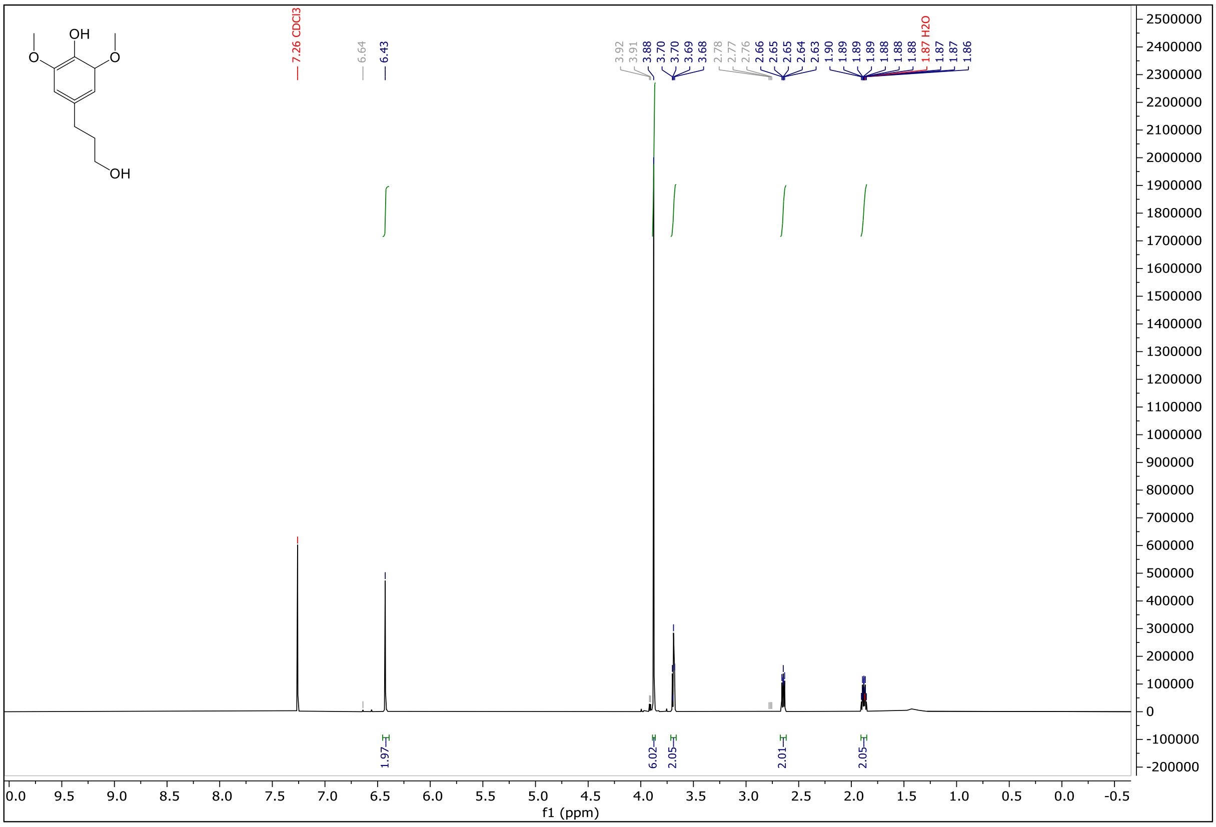

Supplementary Figure 1. ^{1}H NMR spectrum of 1S.

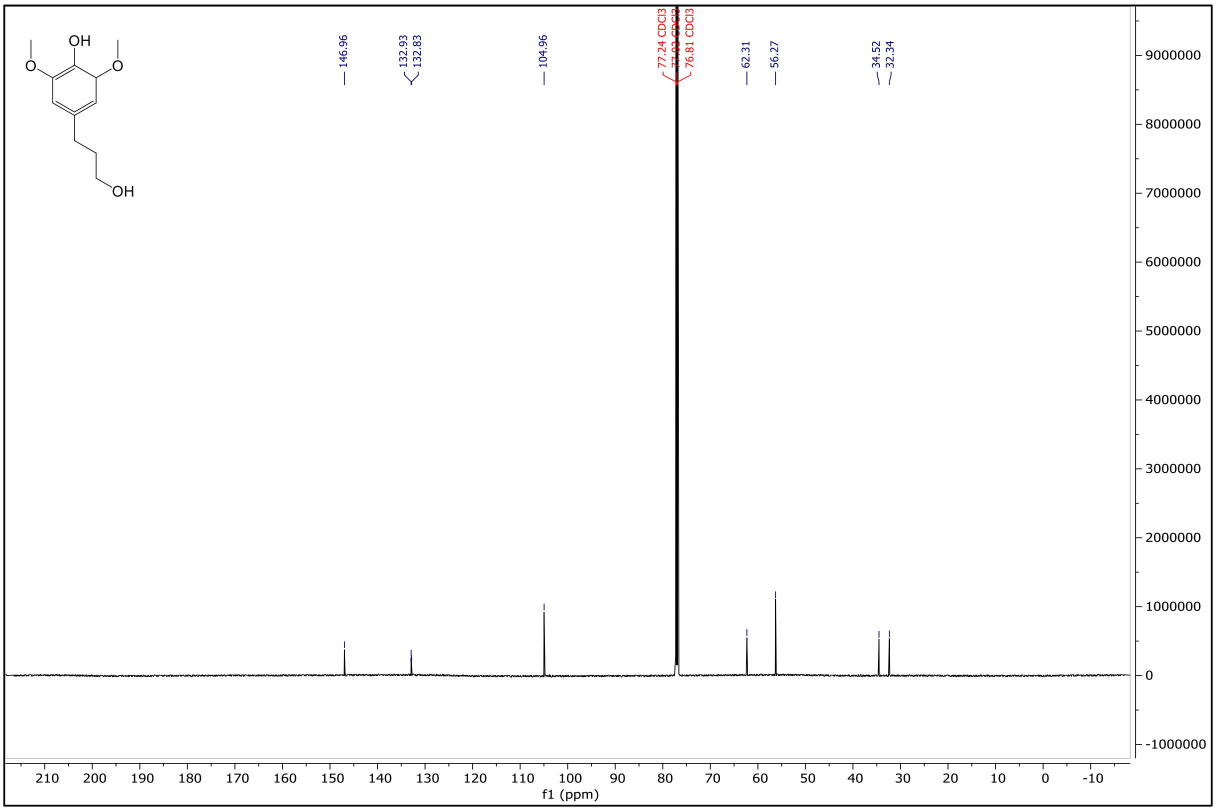

Supplementary Figure 2. ^{13}C NMR spectrum of 1S.

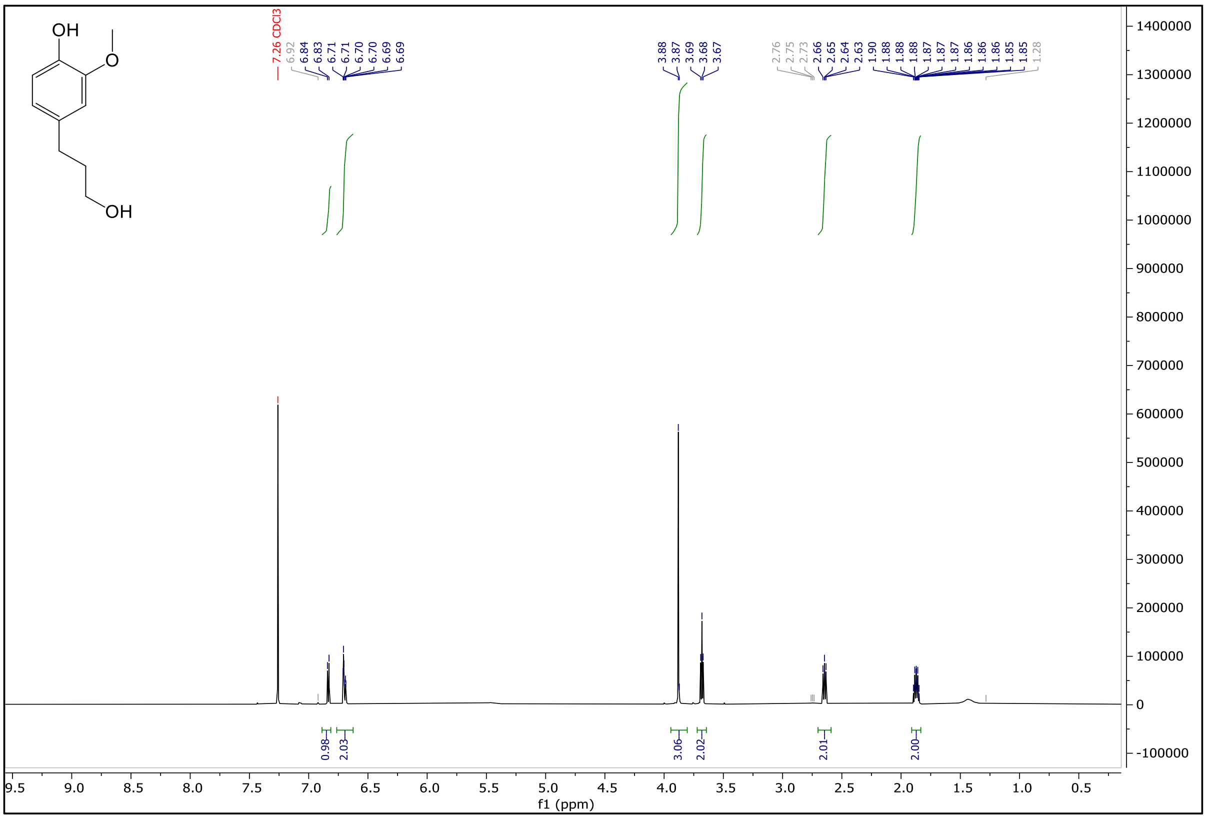

Supplementary Figure 3. ^{1}H NMR spectrum of 1G.

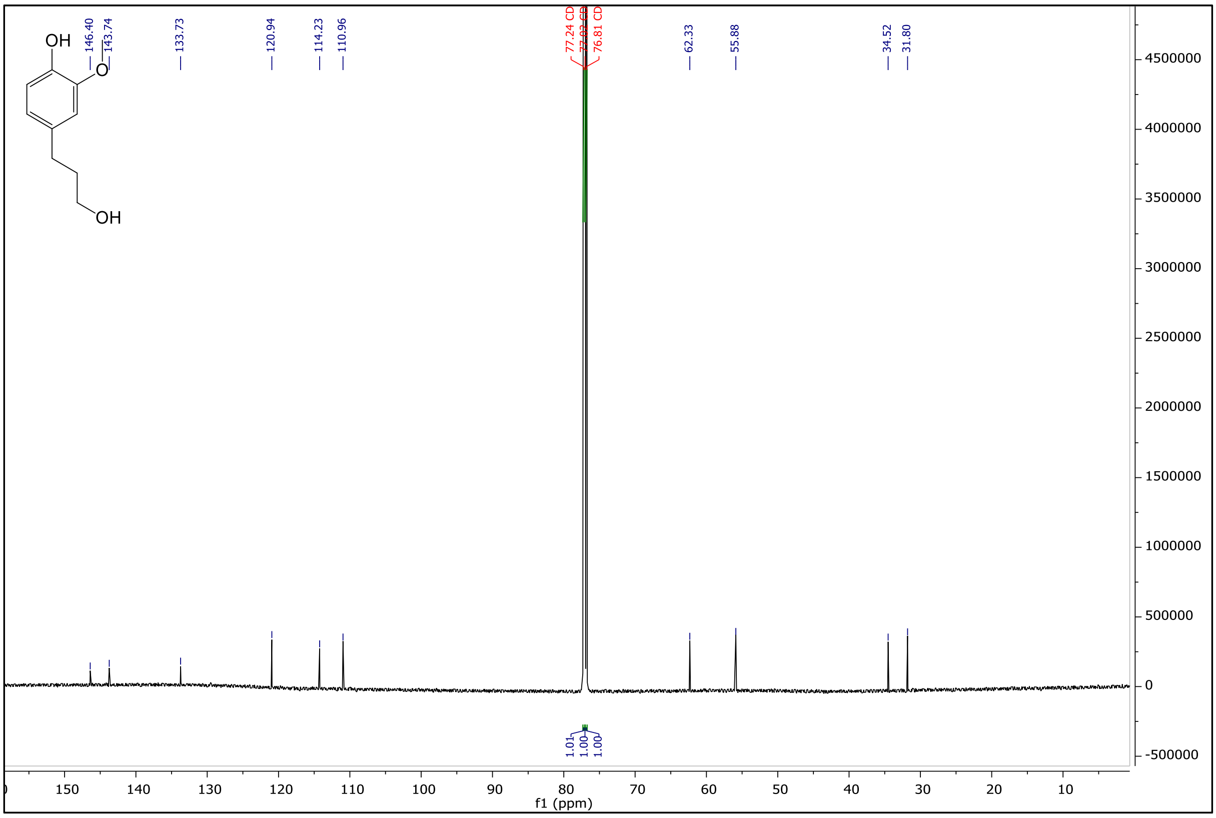

Supplementary Figure 4. ^{13}C NMR spectrum of 1G.

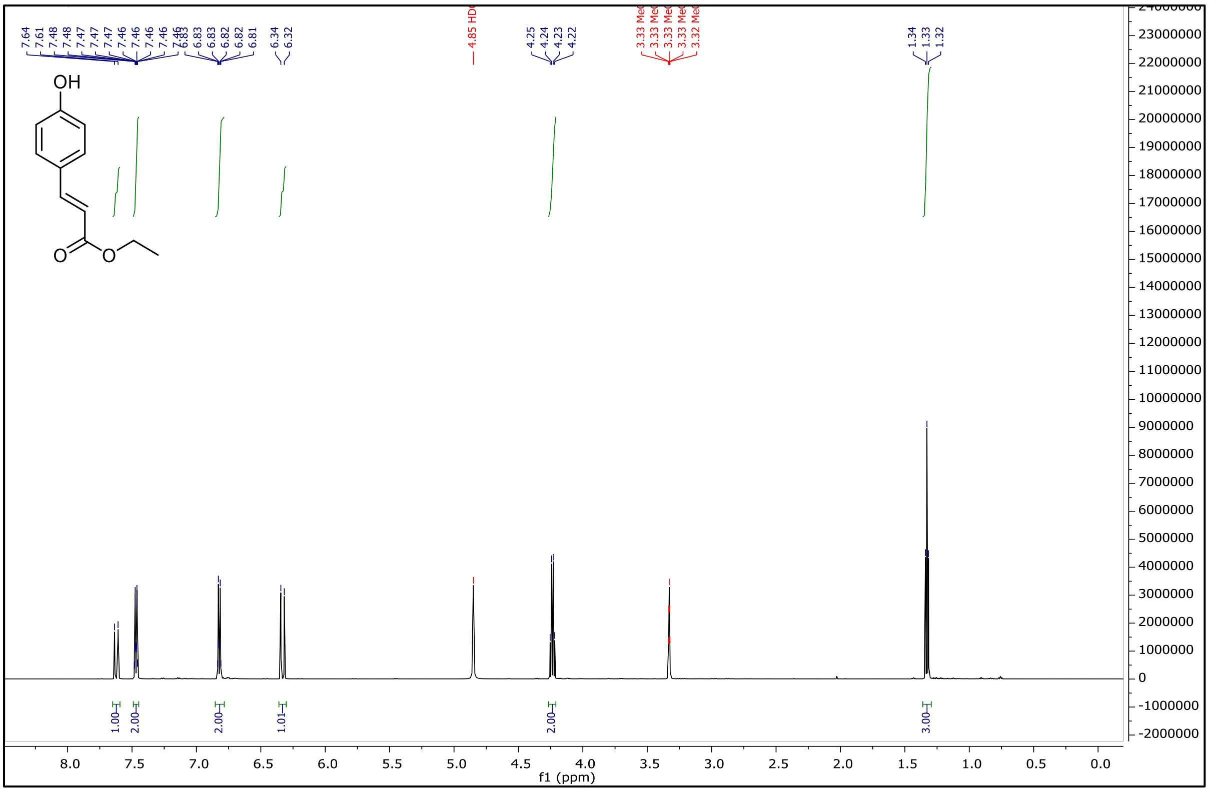

Supplementary Figure 5. ^{1}H NMR spectrum of para-coumaric acid ethyl ester.

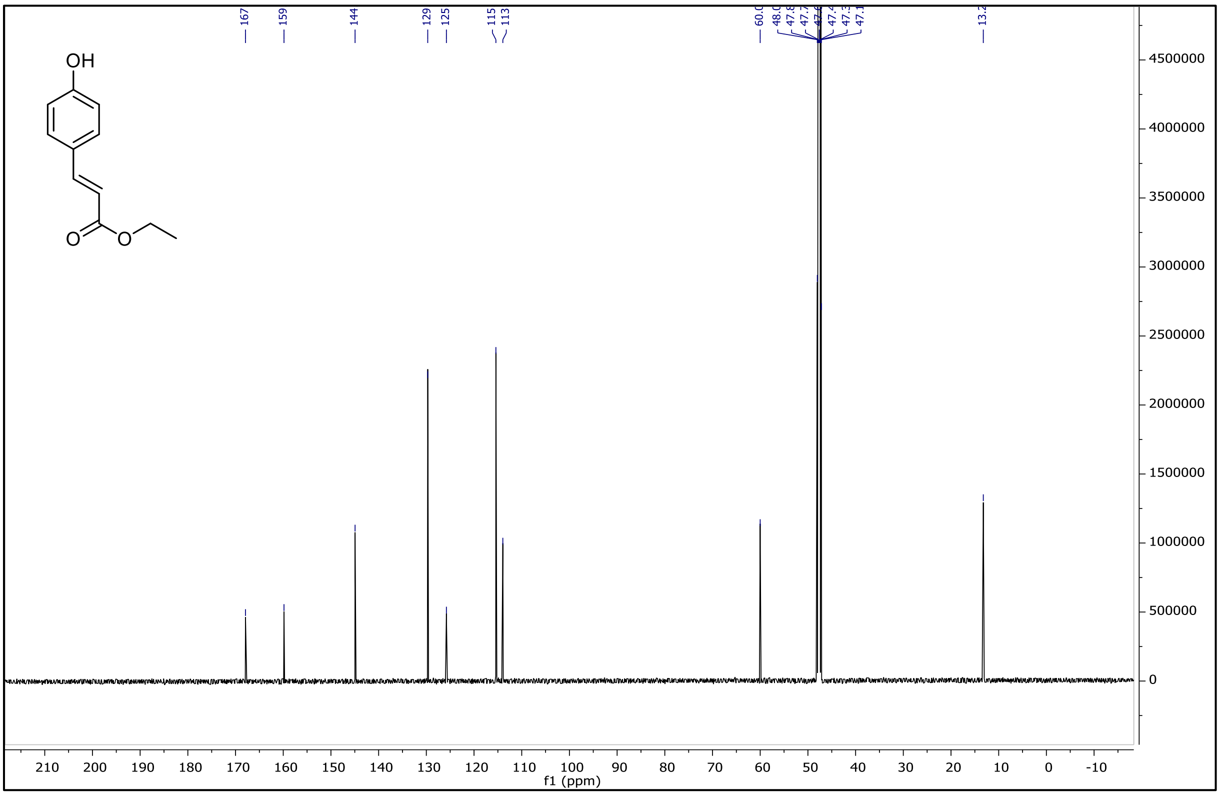

Supplementary Figure 6. ^{13}C NMR spectrum of para-coumaric acid ethyl ester.

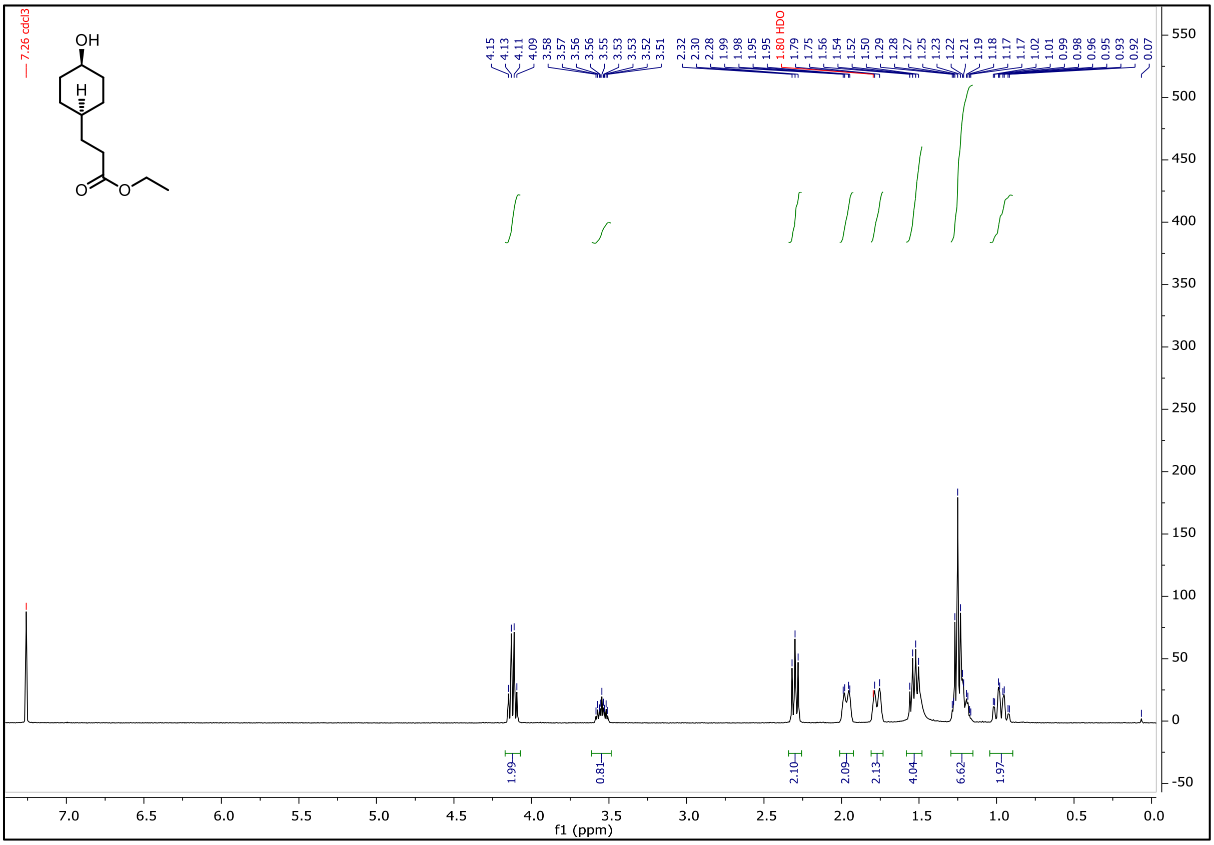

Supplementary Figure 7. ^{1}H NMR spectrum of 3-(4-hydroxycyclohexyl)propionic acid ethyl ester (trans).

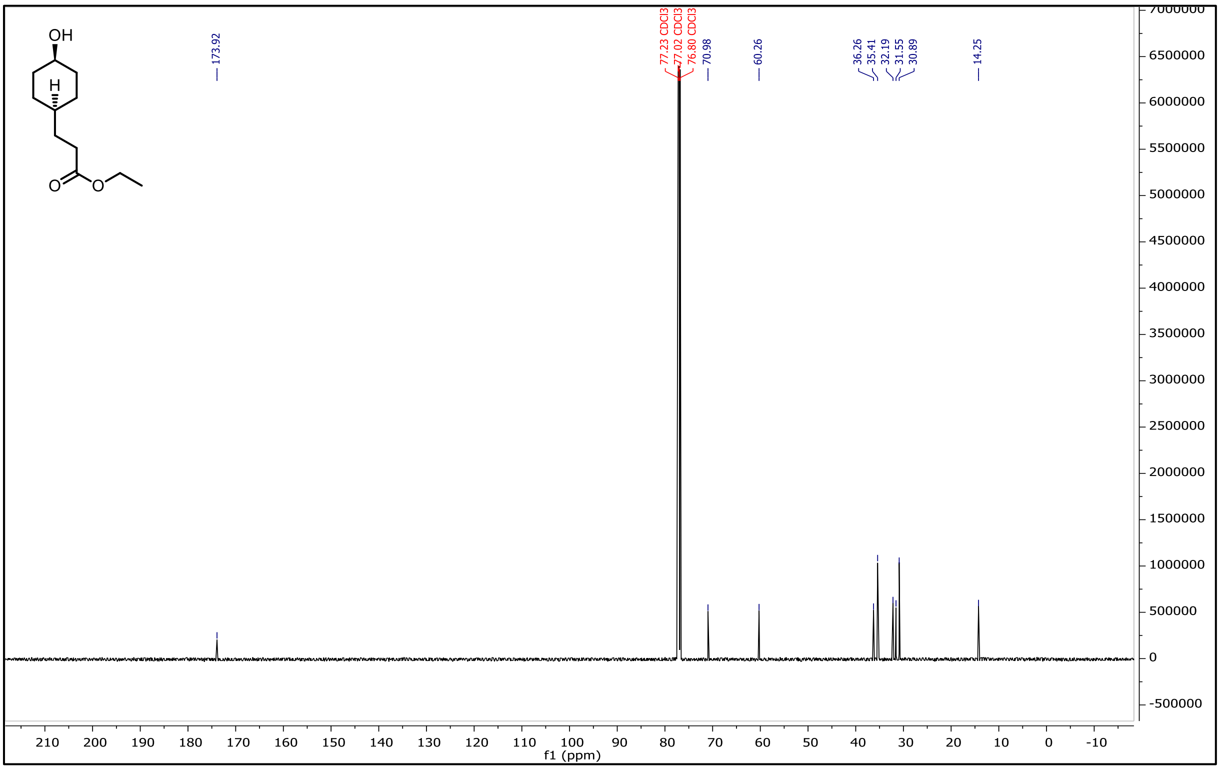

Supplementary Figure 8. ^{13}C NMR spectrum of 3-(4-hydroxycyclohexyl)propionic acid ethyl ester (trans).

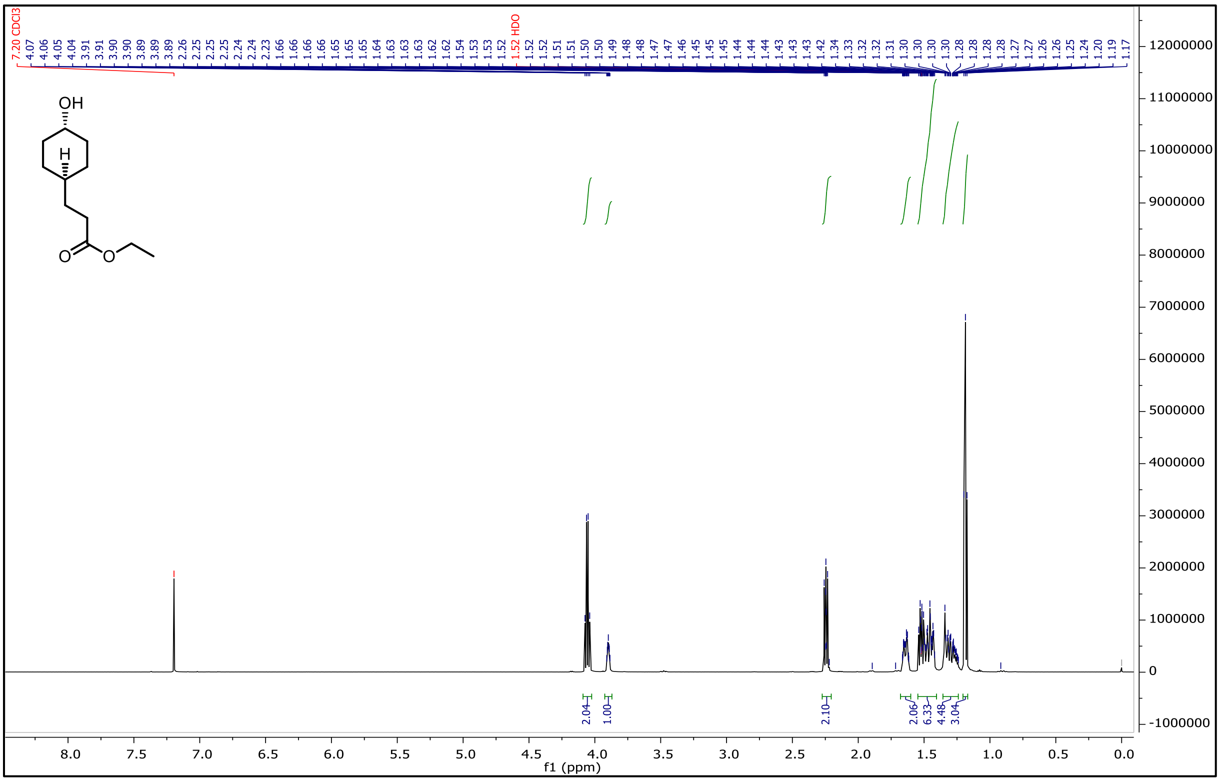

Supplementary Figure 9. ^{1}H NMR spectrum of 3-(4-hydroxycyclohexyl)propionic acid ethyl ester (cis).

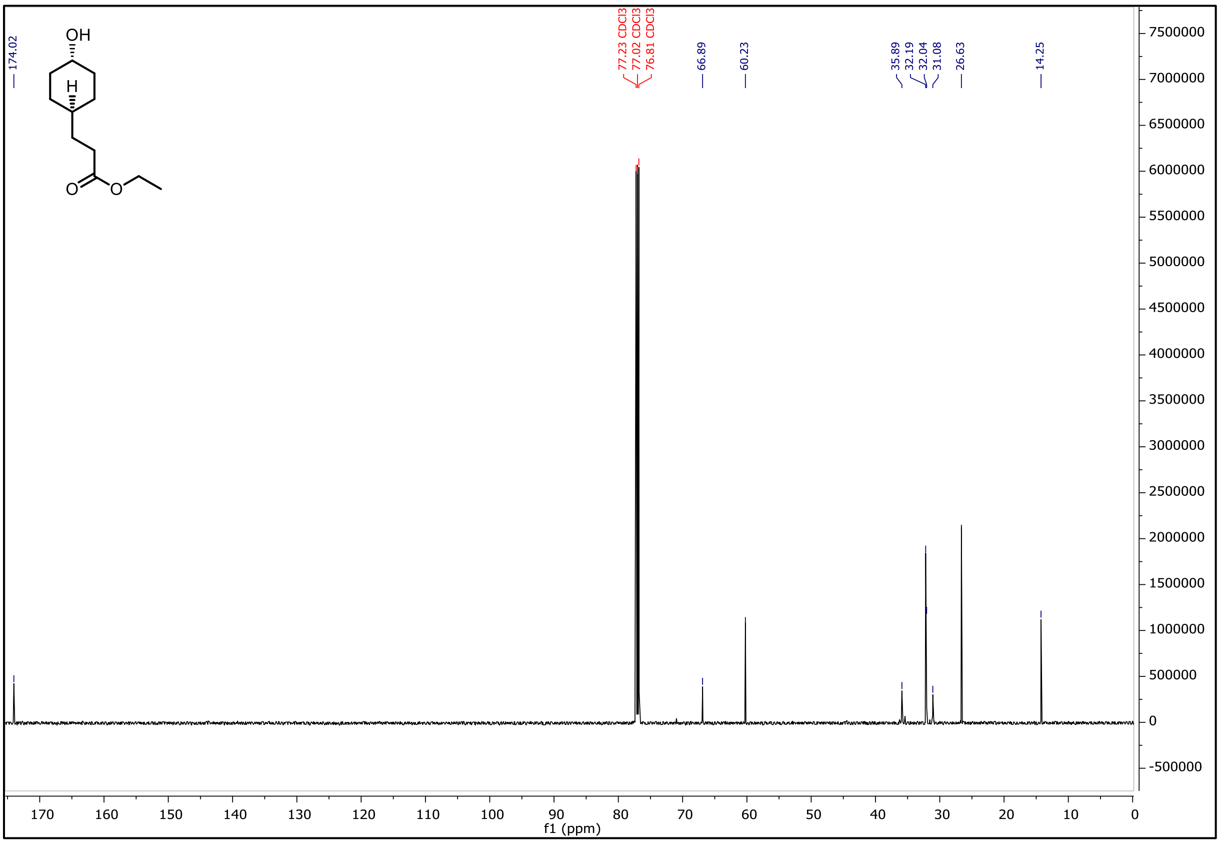

Supplementary Figure 10. ^{13}C NMR spectrum of 3-(4-hydroxycyclohexyl)propionic acid ethyl ester (cis).

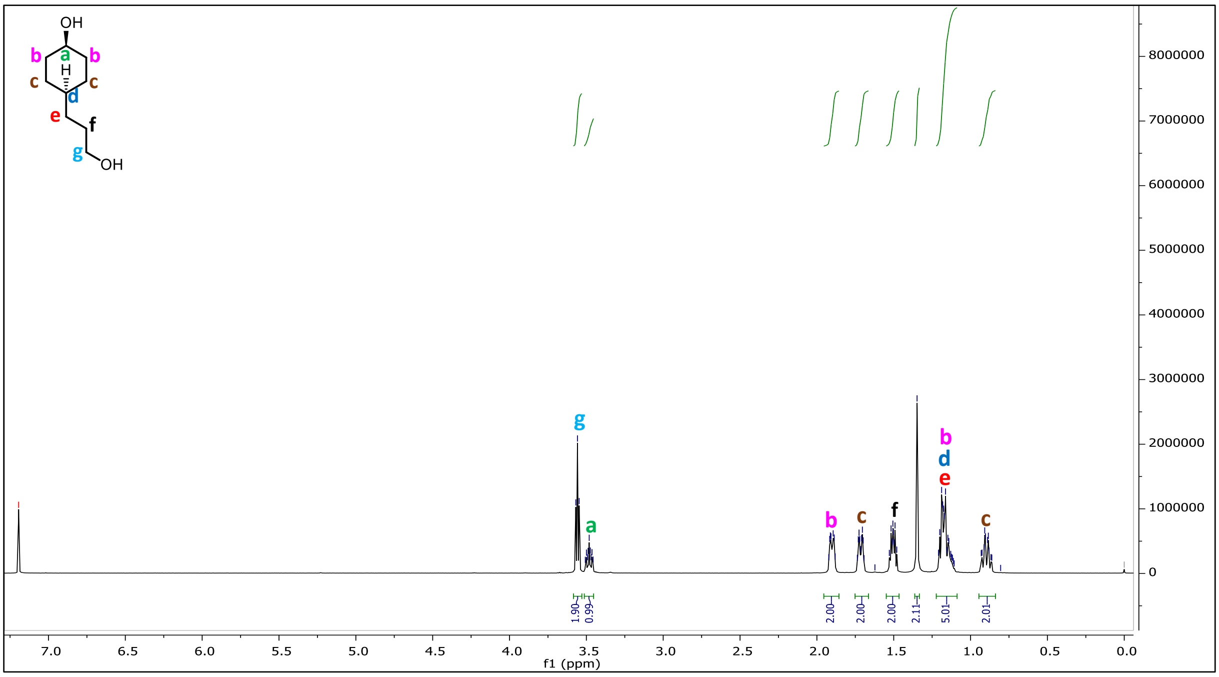

Supplementary Figure 11. ^{1}H NMR spectrum of PC_{trans}.

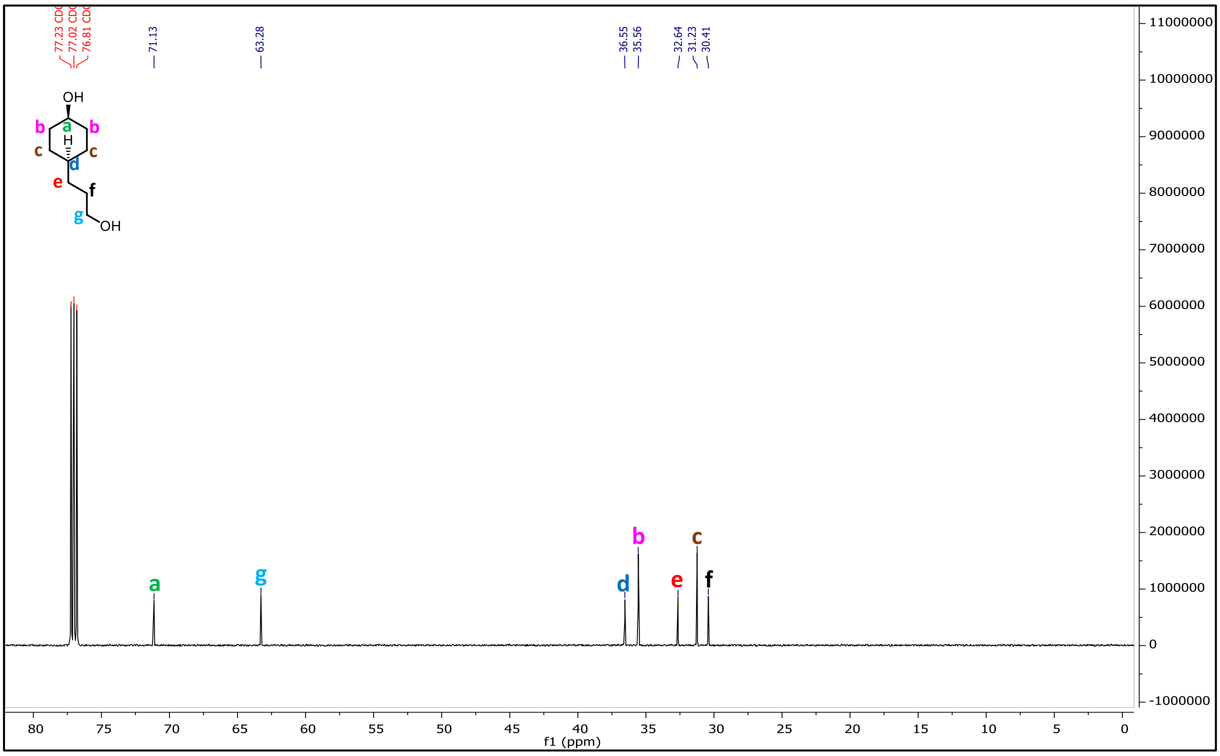

Supplementary Figure 12. ^{13}C NMR spectrum of PC_{trans}.

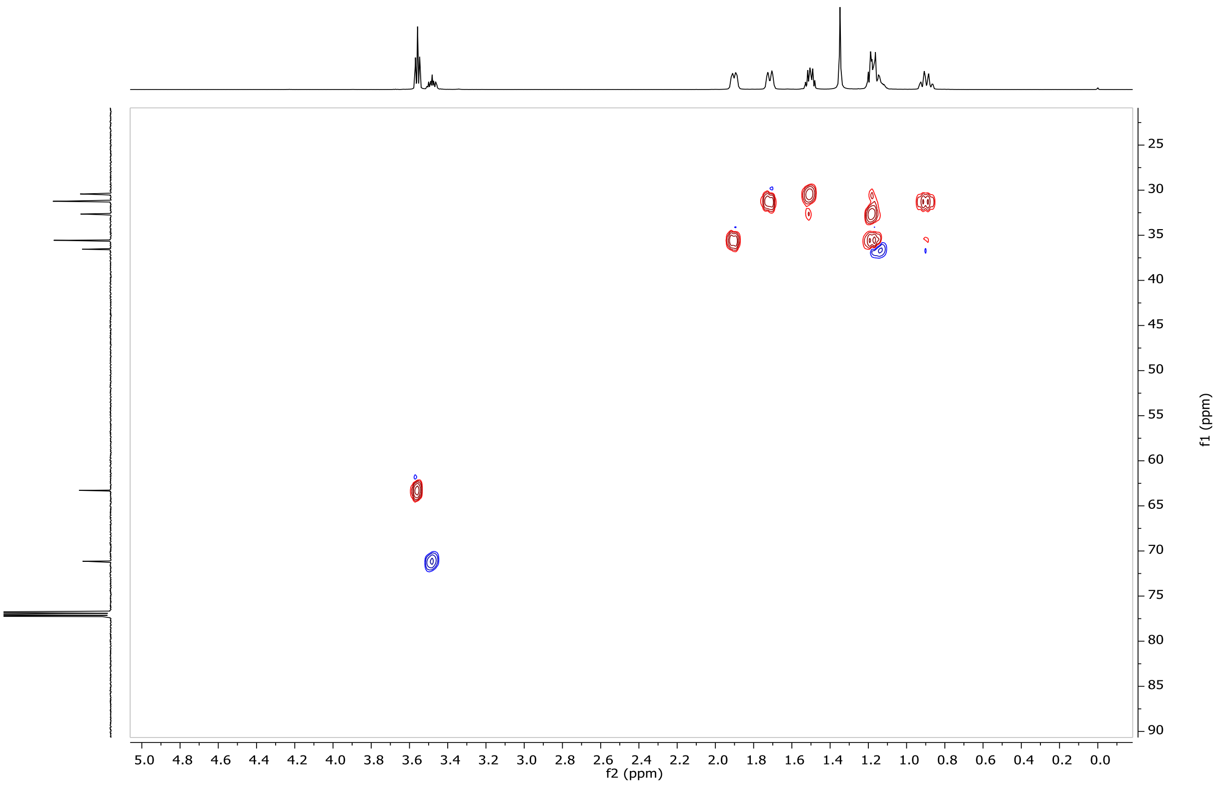

Supplementary Figure 13. 2D HSQC spectrum of PC_{trans}.

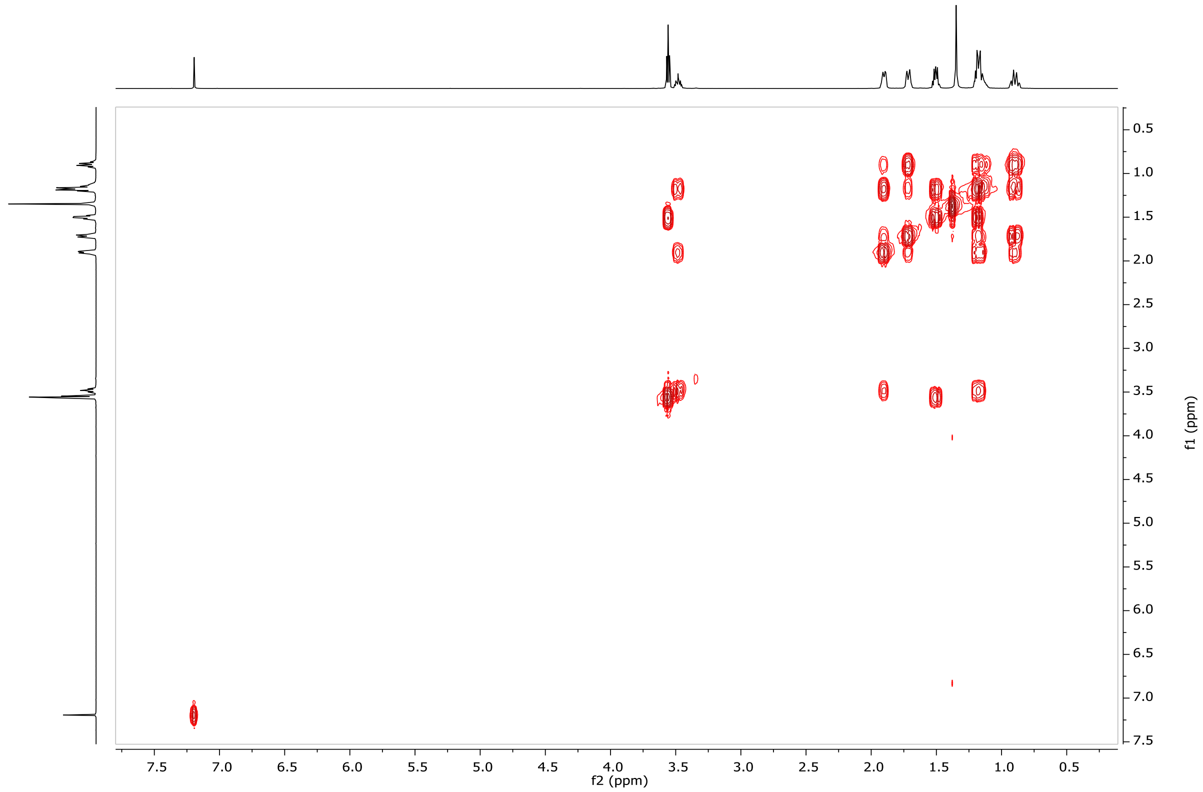

Supplementary Figure 14. 2D COSY spectrum of PC_{trans}.

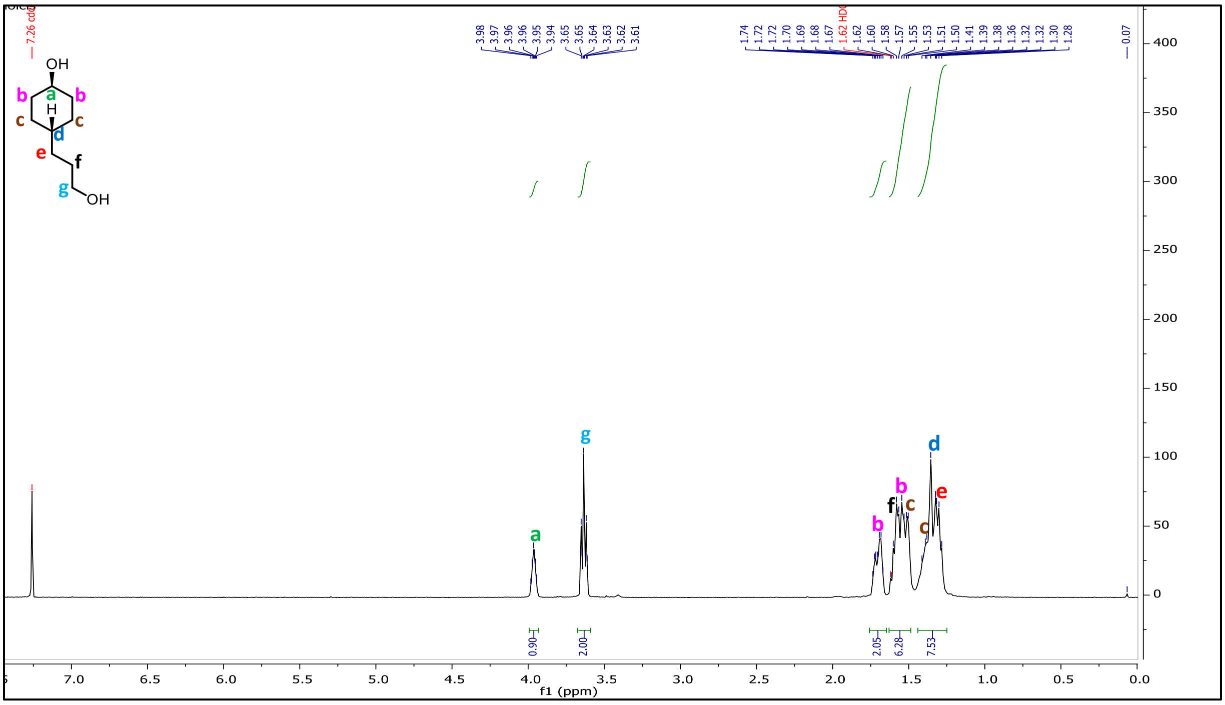

Supplementary Figure 15. ^{1}H NMR spectrum of PC_{cis}.

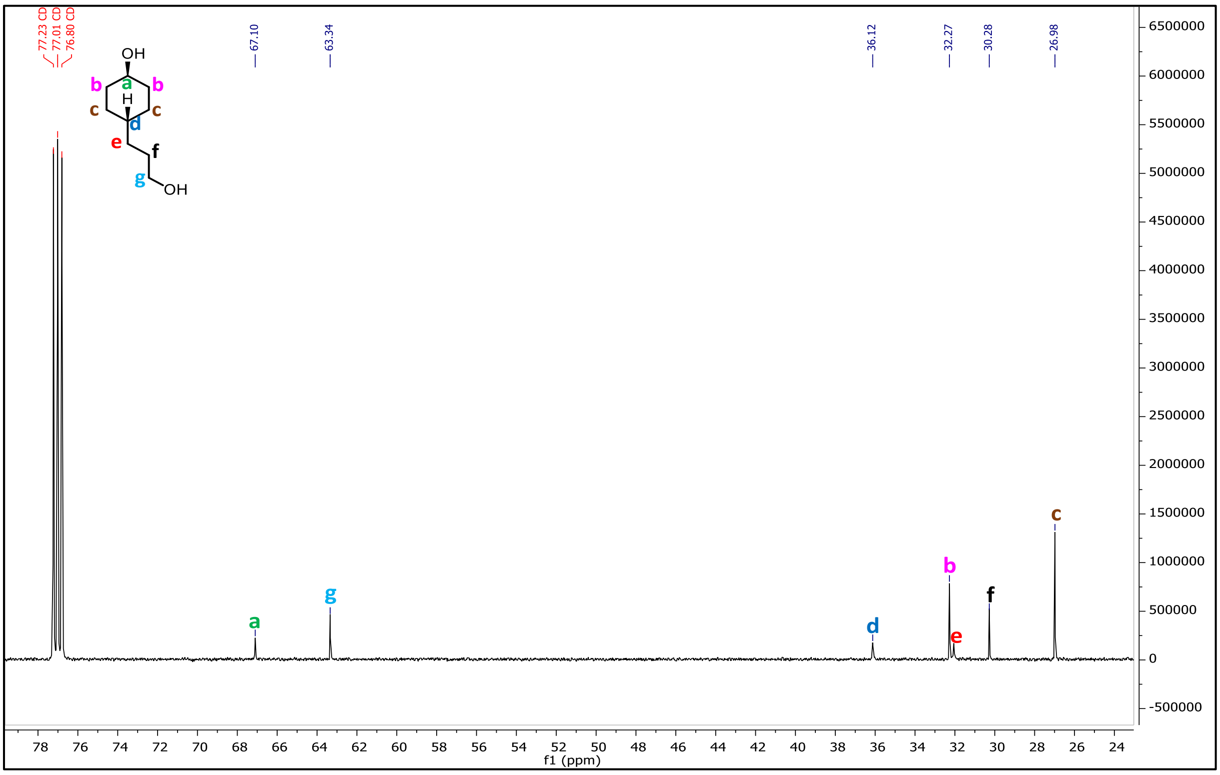

Supplementary Figure 16. ^{13}C NMR spectrum of PC_{cis}.

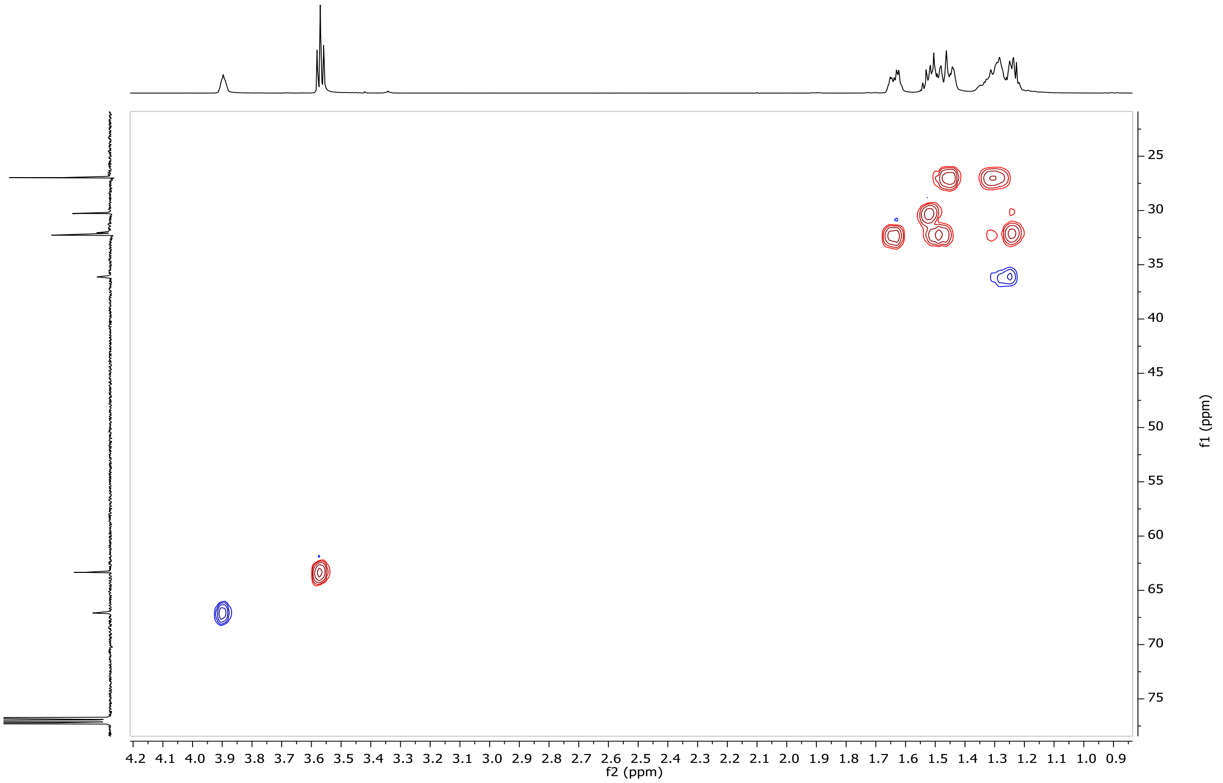

Supplementary Figure 17. 2D HSQC spectrum of PC_{cis}.

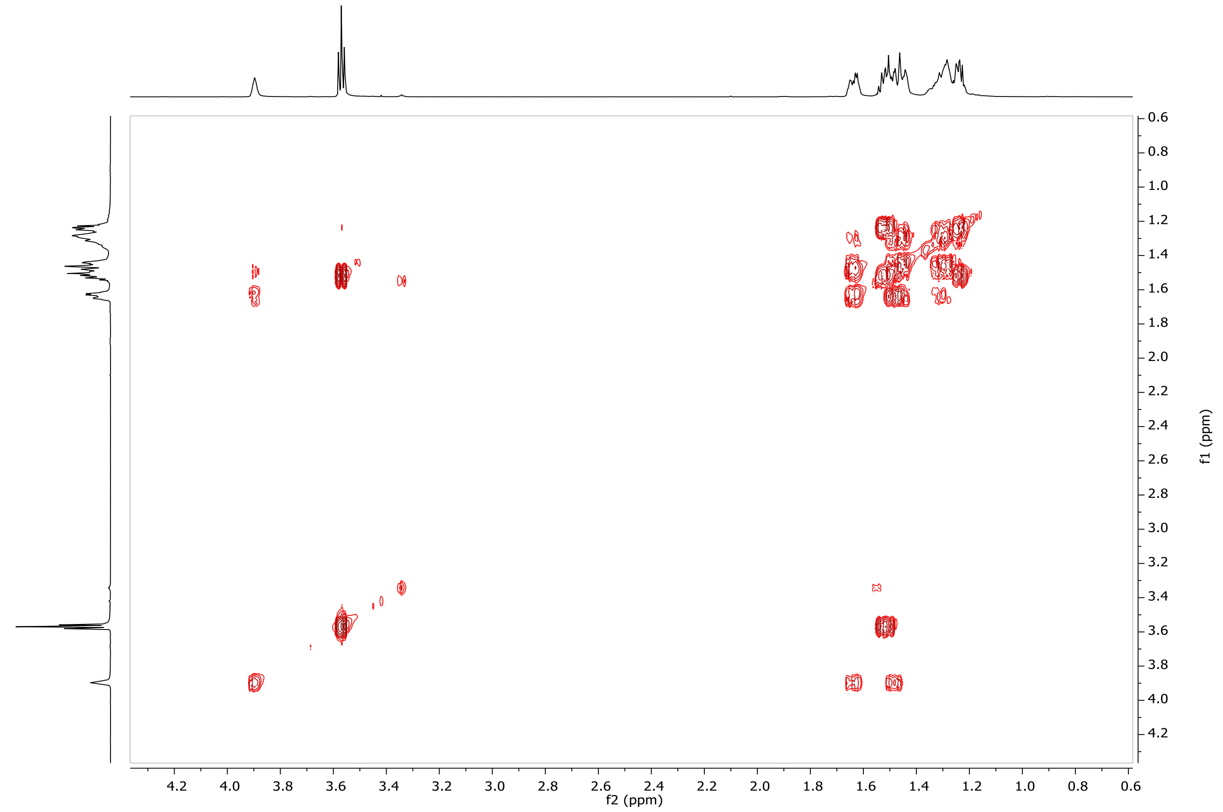

Supplementary Figure 18. 2D COSY spectrum of PC_{cis}.

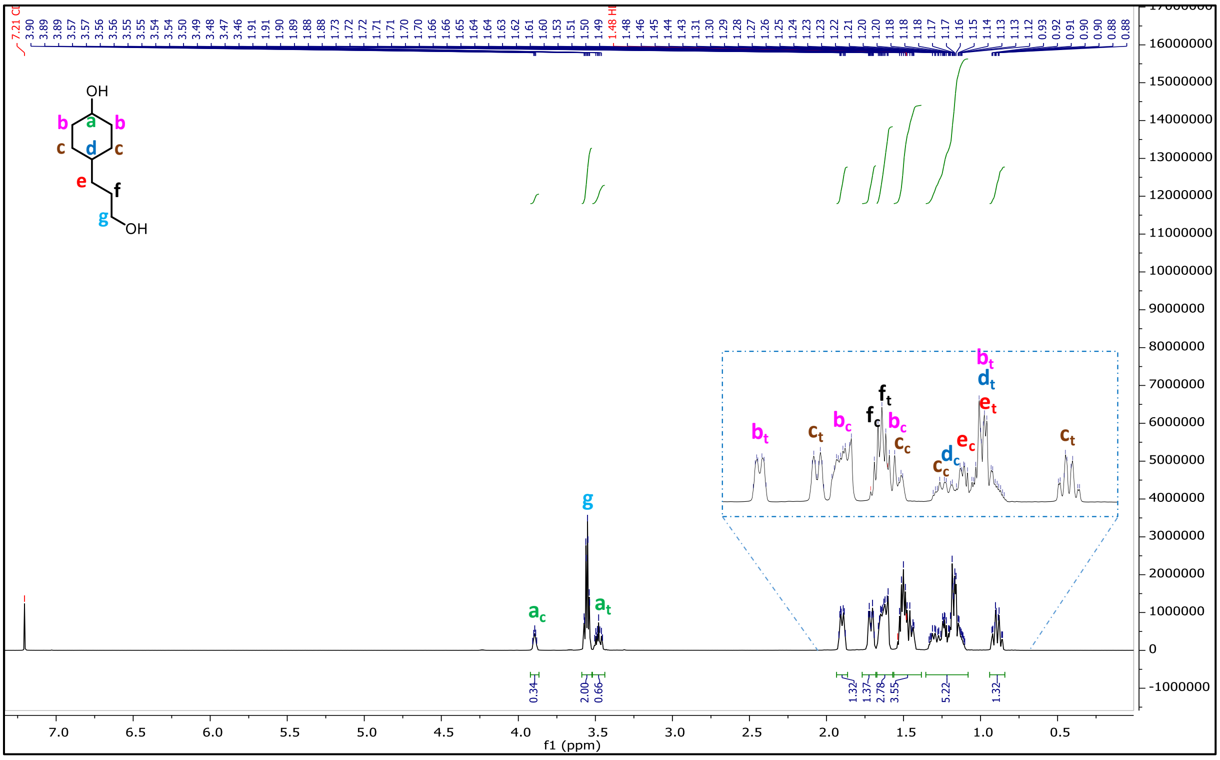

Supplementary Figure 19. ^{1}H NMR spectrum of $PC_{cis\text{-}trans}$.

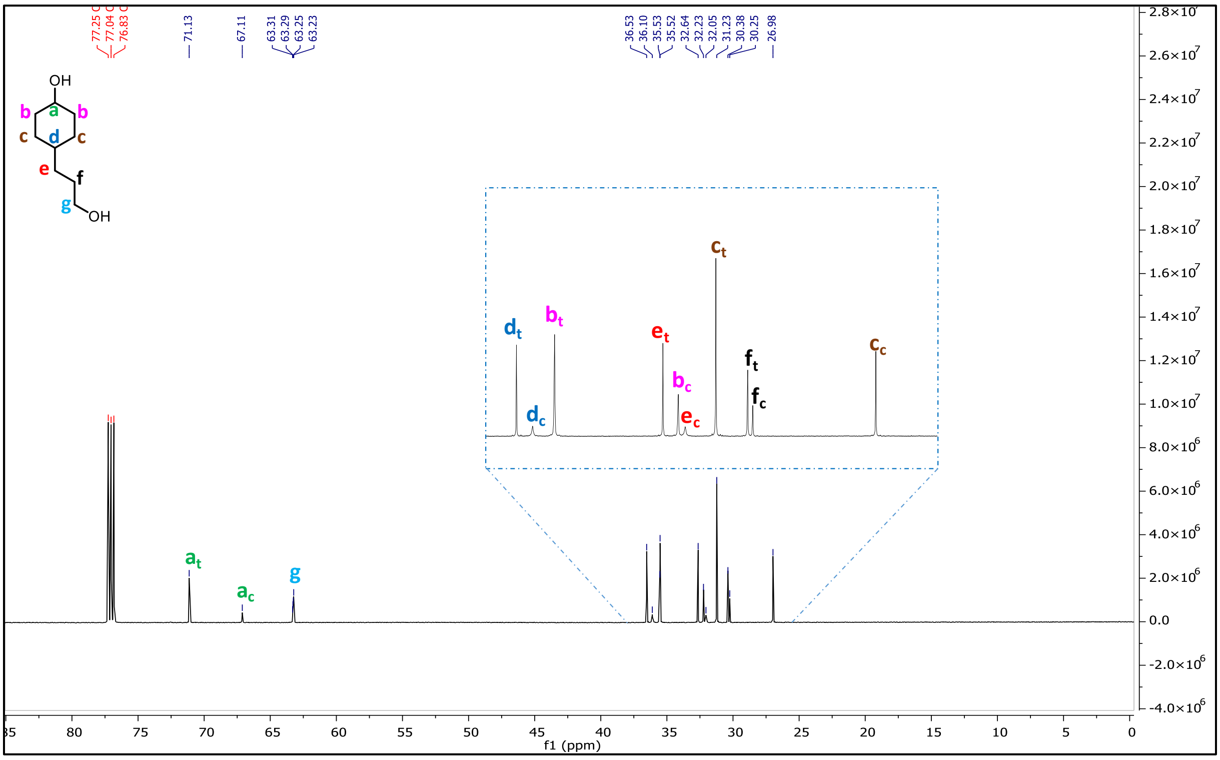

Supplementary Figure 20. ^{13}C NMR spectrum of $PC_{cis\text{-}trans}$.

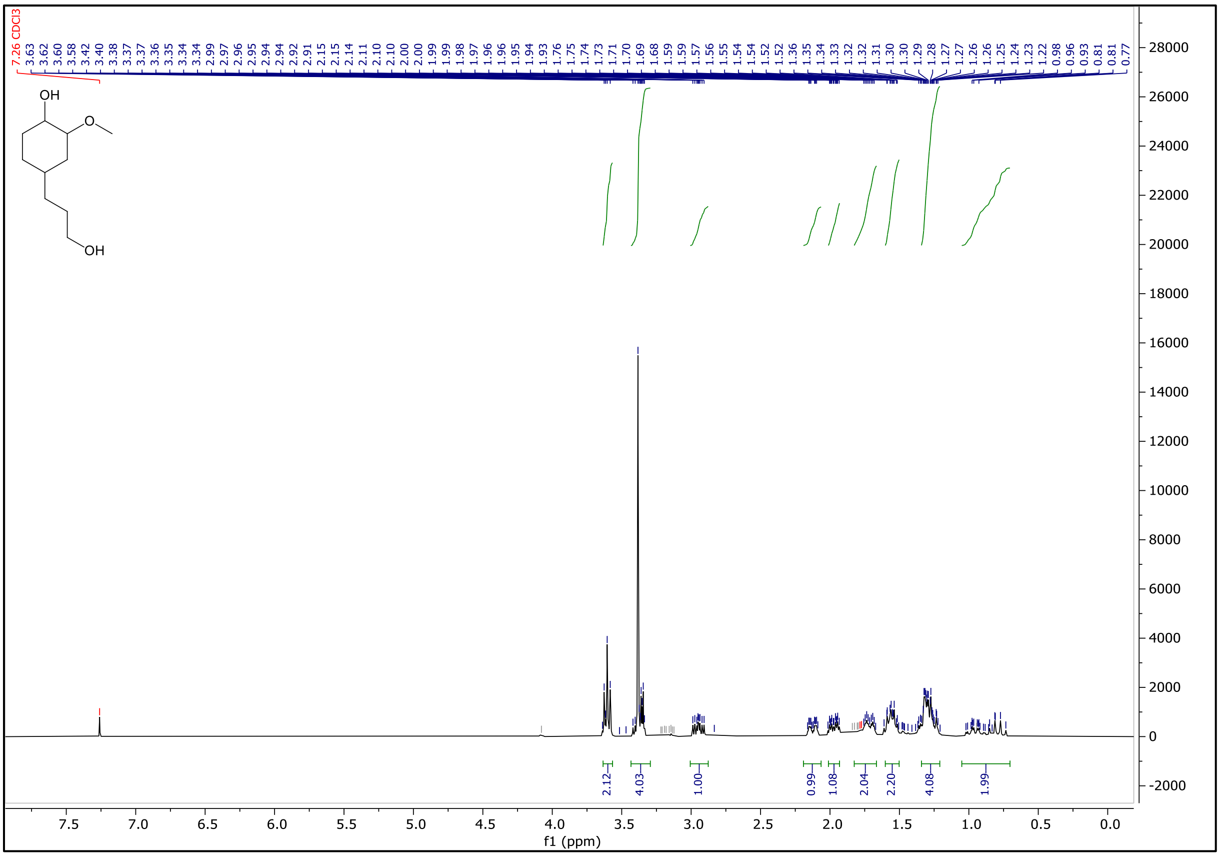

Supplementary Figure 21. ^{1}H NMR spectrum of **1**.

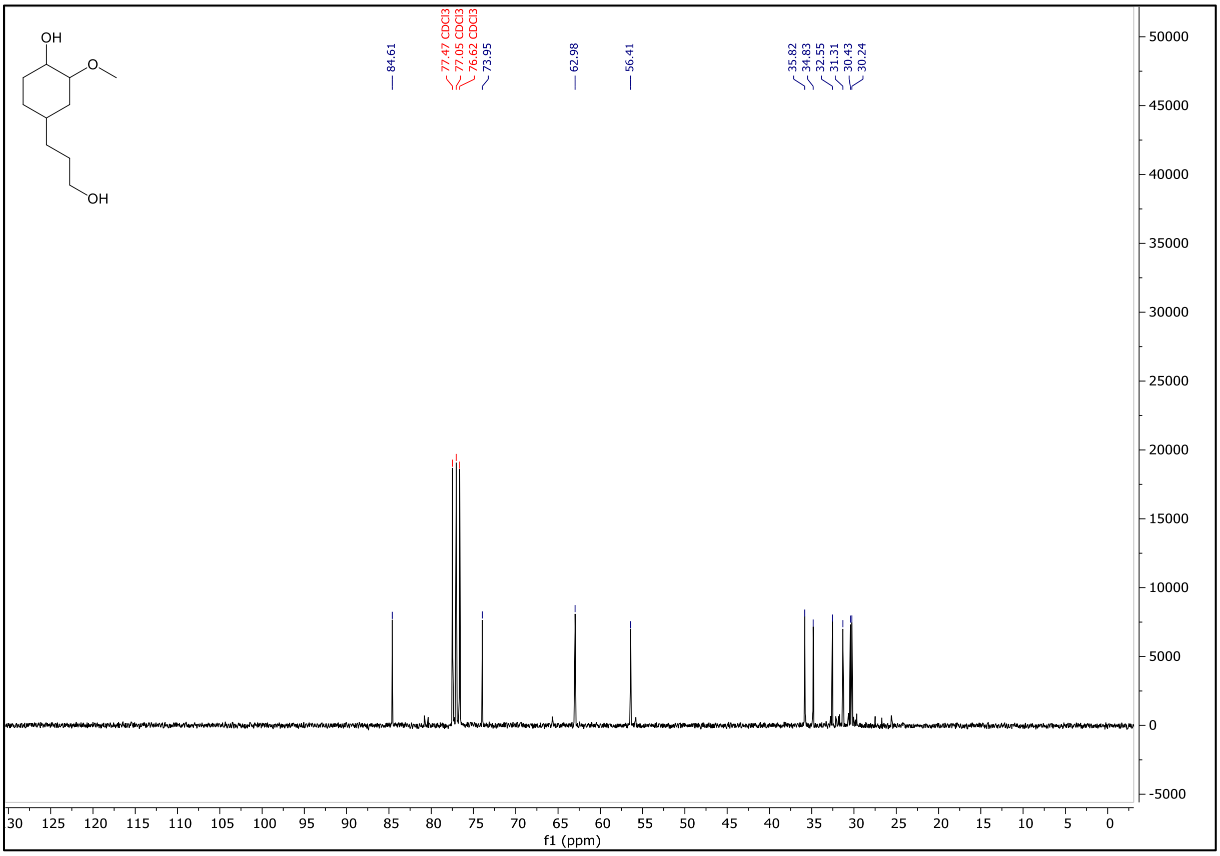

Supplementary Figure 22. ^{13}C NMR spectrum of **1**.

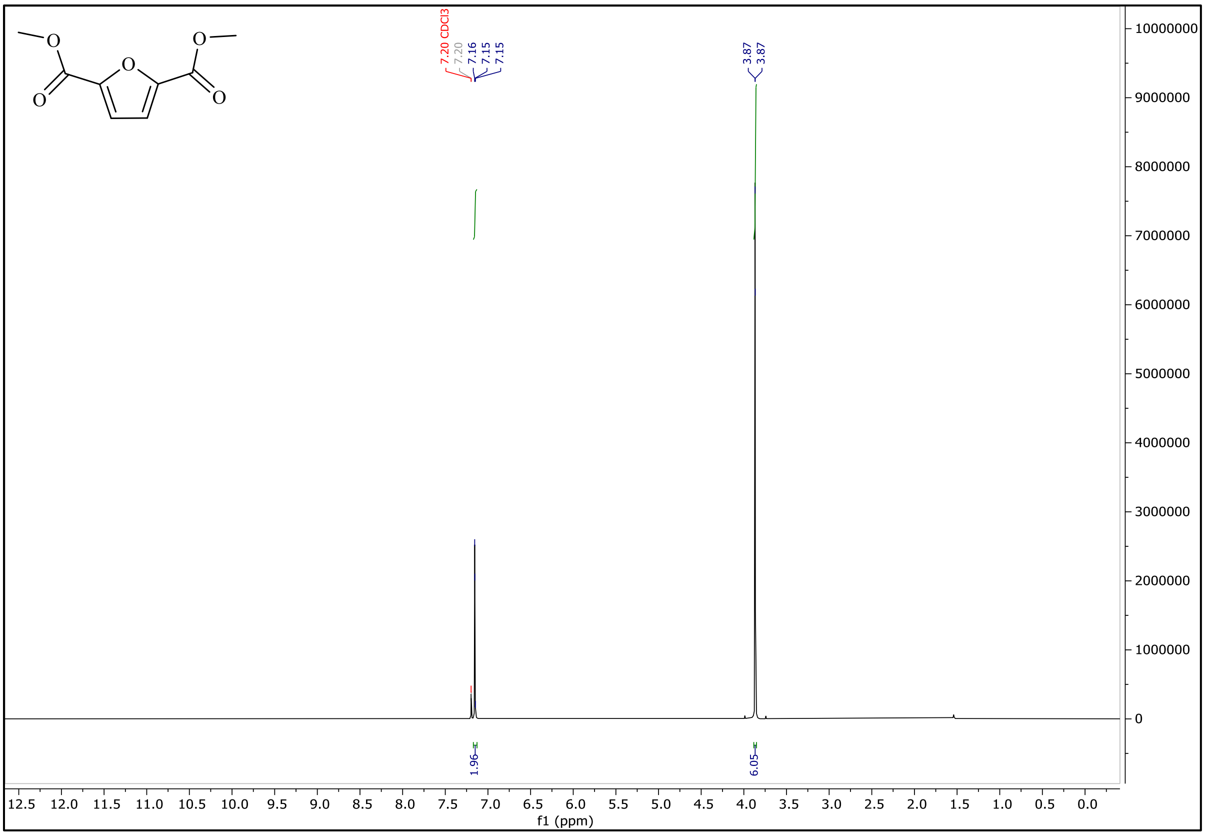

Supplementary Figure 23. ^{1}H NMR spectrum of DMFD.

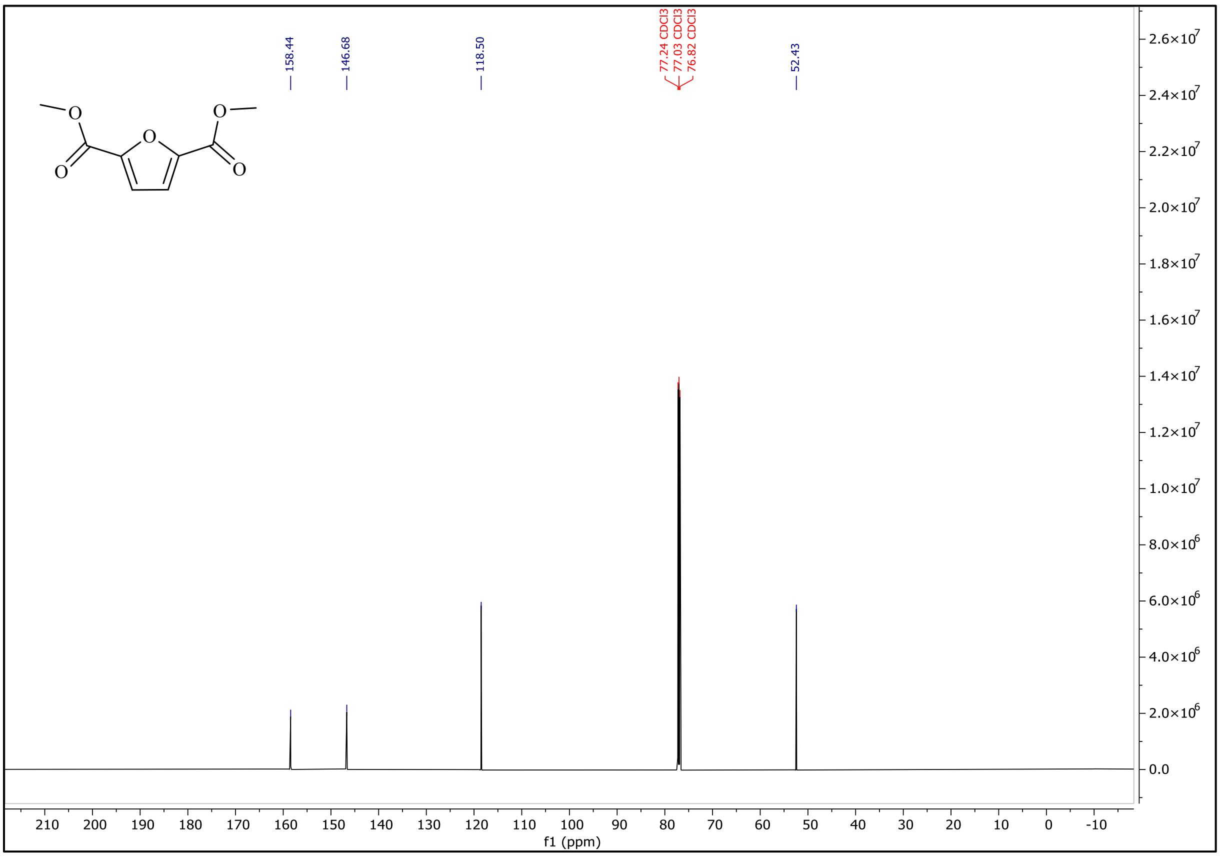

Supplementary Figure 24. ^{13}C NMR spectrum of DMFD.

1.5 General experimental procedures

Reductive catalytic fractionation of lignocellulosic biomass (General procedure A): The mild depolymerization of pine, beech and poplar lignocellulose was carried out in a high-pressure Parr autoclave equipped with an overhead stirrer. Typically, the autoclave was charged with 0.4 g of Cu20-PMO catalyst, 2 g of lignocellulose (beech, pine or poplar) and methanol (20 mL) as a solvent. The reactor was sealed and pressurized with H_2 (40 bar) at room temperature. The reactor was heated to 180 °C and stirred at 400 rpm for 18 h. After completion of the reaction, the reactor was cooled to room temperature. Then 0.1 mL solution was collected through a syringe and injected to GC-MS or GC-FID after filtration through a PTFE filter (0.45 μm). The solid was separated from the solution by centrifugation and subsequent decantation and additionally washed with methanol (3×20 mL). The methanol washings were combined in a round bottom flask and the solvent was removed in vacuo. The crude product was dried in a desiccator in vacuo overnight and was further used as specified below. Fractionation procedure: To the obtained crude mixture, EtOAc (20 mL) was added and it was stirred overnight at room temperature, which resulted in precipitation of brownish colored solid. The suspension was then transferred into a 20 mL centrifuge tube. The solid was separated by centrifugation and decantation and additionally washed with EtOAc (2×20 mL) and dried in vacuo until constant weight . The EtOAc washings were combined in a separating funnel and were washed with small amount of saturated $NaHCO_3$ (1×10 mL) and brine (2×10 mL) and the organic phase was dried over anhydrous $MgSO_4$. After filtration, the solvent was transferred in a round bottom flask and the solvent was removed in vacuo for further use as specified below to give yellow brown crude product.

Demethoxylation/hydrogenation of model compound 1G to 4-n-propanolcyclohexanol (PC) (General procedure B): The demethoxylation/hydrogenation of 1G was carried out in 100 mL high-pressure Parr autoclave equipped with an overhead stirrer. Typically, the autoclave was charged with 1 g Raney Ni catalyst, 0.2 g (1.1 mmol) 1G, 15 mL isopropanol, 20 mg dodecane as internal standard. The reactor was sealed and pressurized with H_2 (10 bar) at room temperature. The reactor was heated to 120 °C and stirred at 400 rpm for 2 h. After completion of the reaction, the reactor was cooled to room temperature. Then 0.1 mL solution was collected through a syringe and injected to GC-MS or GC-FID after filtration through a PTFE filter (0.45 μm). The Raney Ni was separated from the solution by centrifugation and subsequent decantation and additionally washed with isopropanol (3×20 mL). Then the isopropanol soluble fractions were combined in a round bottom flask and the solvent was removed in vacuo. The crude product was dried in a desiccator in vacuo overnight and was further used as specified below.

Copolymerization of PC with DMTA and DMFD (General procedure C): The two-step melt polymerizations (esterification and polycondensation) were performed using equal molar ratio of PC and dimethyl terephthalate (DMTA) or dimethyl 2,5-furandicarboxylate (DMFD) over $Zn(OAc)_2$ or Titanium (IV) butoxide (TBT) as catalyst. For example, a 100 mL three-neck flask equipped with a magnetic stirrer and reflux condenser was charged with 0.53 g (3.35 mmol) of PC diol, 0.66 g (3.35

mmol) of DMTA and 1 mol % (0.0057 g) $Zn(OAc)_2$ catalyst. The esterification reaction was performed at 190 °C for 1 h under nitrogen flow. Then, the reaction temperature was increased to 230 °C and the pressure was reduced to 1 mPa using an oil pulp for 3 h. The same experiments were carried out under the same reaction conditions but polymerization at 230 °C for 1h for comparable purpose. After that, the reaction mixture was cooled down to room temperature and the pressure was returned to atmospheric pressure by introducing nitrogen. The off-white solid poly (PC/TPA)-3h (0.71 g) was obtained in a yield of 72.1 % and was characterized by NMR, DSC, TGA, FTIR and GPC. Then poly (PC/TPA)-3h was dissolved in chloroform (5 mL) and precipitated with methanol (50 mL) to get purified poly (PC/TPA) solid (0.49 g) in a yield of 50.8 %.

Chemical recycling of the synthesized poly (PC/TPA) (General procedure D): The mild depolymerization of the synthesized poly (PC/TPA) through alcoholysis was carried out in a 100 mL high pressure Parr autoclave equipped with an overhead stirrer. Typically, the autoclave was charged with the respective polymer, such as poly (PC/TPA) (0.2 g), dodecane (as internal standard) (20 mg) and methanol (30 mL). The reactor was sealed and flushed with N_2 three times. The reactor was heated to 190 °C and stirred at 400 rpm for 4 h. After completion of the reaction, the reactor was cooled to room temperature. The mixture rich in PC was then isolated and purified by column chromatography using EtOAc/n-hexane (1:2 to 1:1 to 2:1)

Hydrodeoxygenation (HDO) of aliphatic oxygenated biomass derived fractions (General procedure E): The hydrodeoxygenation (HDO) of aliphatic oxygenated biomass derived fractions was carried out in a 100 mL high pressure Parr autoclave equipped with an overhead stirrer. Typically, the autoclave was charged with 50 mg of starting material, 200 mg of Raney Ni, 100 mg of HZSM-5 catalyst, 5 mg of dodecane and 20 mL of cyclohexane. The reactor was sealed and pressurized with 30 bar of H_2. Then the mixture was heated to 220 °C and stirred for 4 h or 6 h. After completion of the reaction, the reactor was cooled to room temperature. Then 0.1 mL solution was collected through a syringe and injected to GC-MS or GC-FID after filtration through a PTFE filter (0.45 μm).

Supplementary Note 1. A comprehensive strategy for the production of PC, DMTA, DMFD, ethanol, furfural and ethylene glycol entirely from lignocellulose

Case 1: Production of fully wood-based PET analogue poly (PC/TPA)

PC from lignocellulose: The weight of PC was determined experimentally using 2000 mg beech wood as the starting material. From the analysis as described in Supplementary Figure 25, 2000 mg beech lignocellulose was subjected to RCF over Cu20-PMO in methanol to give crude lignin oil rich in 1G and 1S with a yield of 24.3 %, which was further subjected to catalytic funneling over Raney Ni/isopropanol to deliver crude aliphatic alcohol mixture with PC as main component. PC was obtained as 52.4 mg (0.3316 mmol) calculated by GC yield, corresponding to a yield of 13.9 %.

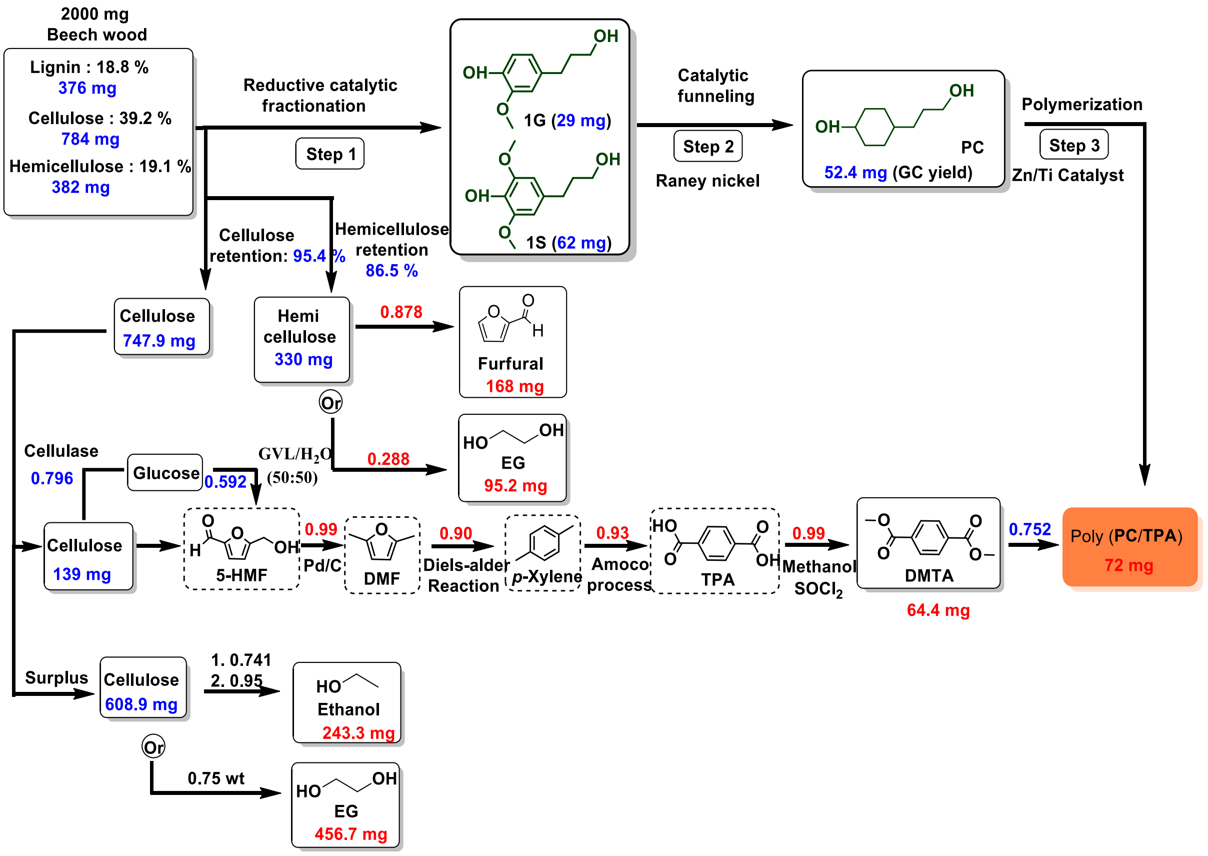

Supplementary Figure 25 A comprehensive proposed strategy for the production of fully wood-based PET analogues. The calculation includes the two-step RCF/catalytic funneling strategy presented in this paper, in combination with literature data for the conversion of the carbohydrate pulp fraction to DMTA, ethanol, furfural and ethylene glycol. Blue number stands for experimental data while red number stands for the data obtained based on literatures.The chemical composition of lignocellulose and carbohydrate pulp was determined following National Renewable Energy laboratory's (NREL) standard analytical procedure.

Weight determination of cellulose for the production of poly (PC/TPA):

The following calculations are relying on experimental data: RCF of 2 g beech lignocellulose (comprising 39.2 wt % cellulose) proceeds with cellulose retention up to 95.4 % in the investigated

reaction conditions. Thus, the amount of usable cellulose is 747.9 mg (747.9 = 784 × 0.954 mg). Cellulose is subjected to enzymatic hydrolysis to glucose in the yield of 79.6 % over cellulase at 50 °C for 72 h, following by isomerization and dehydration to 5-HMF (59.2 % yield) over $AlCl_3$ catalyst at 120 °C for 4 h.

The following calculations are relying on selected literature routes/examples: The obtained 5-HMF was then subjected to hydrogenation/hydrogenolysis to DMF (99 % yield) over Pd/C catalyst[7]. DMF is further converted to p-xylene (90 %) by Diels-Alder cycloaddition with ethylene followed by dehydration over a H-Beta zeolite catalyst[8]. TPA can be obtained commercially by oxidation of p-xylene (93 %) over cobalt-manganese-bromide catalyst (Amoco process)[9], followed by methyl ester formation in a yield of 99 % (DMTA)[10].

Based on the values above, 0.3316 mmol of PC diol would require 0.3316 mmol of DMTA to produce 72 mg poly (PC/TPA). This theoretically requires the following amount of cellulose:

$$\text{Cellulose (mg)} = 162 \times 0.3316 \div (0.99 \times 0.93 \times 0.90 \times 0.99 \times 0.592 \times 0.796) = 139 \text{ mg} \quad \text{Eq10}$$

Therefore, the available amount of cellulose definitely covers the need for the diacid building block from the same wood. The surplus of cellulose is 608.9 mg (608.9 mg = 747.9 – 139 mg).

Calculating theoretical maximum PC yield and cellulose needed:

RCF and 100 % yield for catalytic funneling, as well as 100% yield of polymer synthesis would provide the following PC amount:

The theoretical maximum aromatic monomers yield (~ 34 % (0.34 = 0.58 × 0.58)) is determined by the β-O-4 content (~ 58 %) in beech wood[11]. Because S/G ratio in beech wood is 2.8[1], the theorectical amount of 1G and 1S should be 8.89 % and 24.9 %, respectively. Thus, the RCF of 2000 mg beech wood (376 mg lignin) would give 33.42 mg of 1G and 92.5 mg of 1S, which can then be funneled to give 92.95 mg, 0.5883 mmol of PC in the maximum yield (24.7 %). Thus this accounts for 169.4 mg poly (PC/TPA), quantified as follows:

$$\text{Poly (PC/TPA) (mg)} = 0.5883 \times (158 + 194 - 32 \times 2) \times 1 = 169.4 \text{ mg} \quad \text{Eq11}$$

For this, 113.5 mg DMTA and consequently 245 mg cellulose are needed, followed by the calculation shown above.

Thus, the surplus of cellulose in this case is (747.9-245 = 502.9 mg).

Option 1: determination of ethanol weight from excess cellulose.

Surplus of 608.9 mg cellulose, as calculated above can be transformed into bio-ethanol. One mol of glucose, yields two moles of ethanol. For a process with 74.1 efficiency (from cellulose to fructose)[12] and 95 % efficiency (from fructose to ethanol)[13], the ethanol mass would be:

$$\text{Ethanol (mg)} = \frac{608.9}{162.1} \times 0.741 \times 0.95 \times 2 \times 46 \text{ mg} = 243.3 \text{ mg} \quad \text{Eq12}$$

Option 2: determination of ethylene glycol weight from excess cellulose.

Recently cellulose has been efficiently converted to ethylene glycol (EG) with 75 wt % yield using Ni-W_2C/MC catalyst in water[14]. Thus following this procedure, complete conversion of the surplus cellulose (608.9 mg) would lead to 456.7 mg EG (456.7 mg = 608.9 × 0.75 mg).

<u>Weight determination of hemicellulose for the production of other valuable products:</u> RCF of 2 g beech lignocellulose (comprising 19.1 wt % cellulose) proceeds with hemi(cellulose) retention up to 86.5 % in the investigated reaction conditions. RCF of 2 g beech lignocellulose (comprising 19.1 wt % hemicellulose) proceeds with hemi(cellulose) retention up to 86.5 % in the investigated reaction conditions. The amount of hemicellulose after RCF of 2 g beech lignocellulose is:

$$\text{Hemicellulose (mg)} = 2000 \times 0.191 \times 0.865 = 330.4 \text{ mg} \qquad \text{Eq13}$$

Hemicellulose has been demonstrated as interesting starting material towards the production of value added products (furfural and ethylene glycol). For fufural, with a yield of 87.8 %, as reported using $Al_2(SO_4)_3$ catalyst in GVL/water[15] from xylan, thus furfural can be quantified (168 mg = 330.4 ÷ 166 × 96 × 0.878 mg). For ethylene glycol, with a combined yield of 28.8 wt % from hemicellulose reported from previous work[16], thus ethylene glycol can be quantified (95.2 mg = 330.4 × 0.288 mg)

Case 2: Production of fully wood-based PET analogue poly (PC/FDCA)

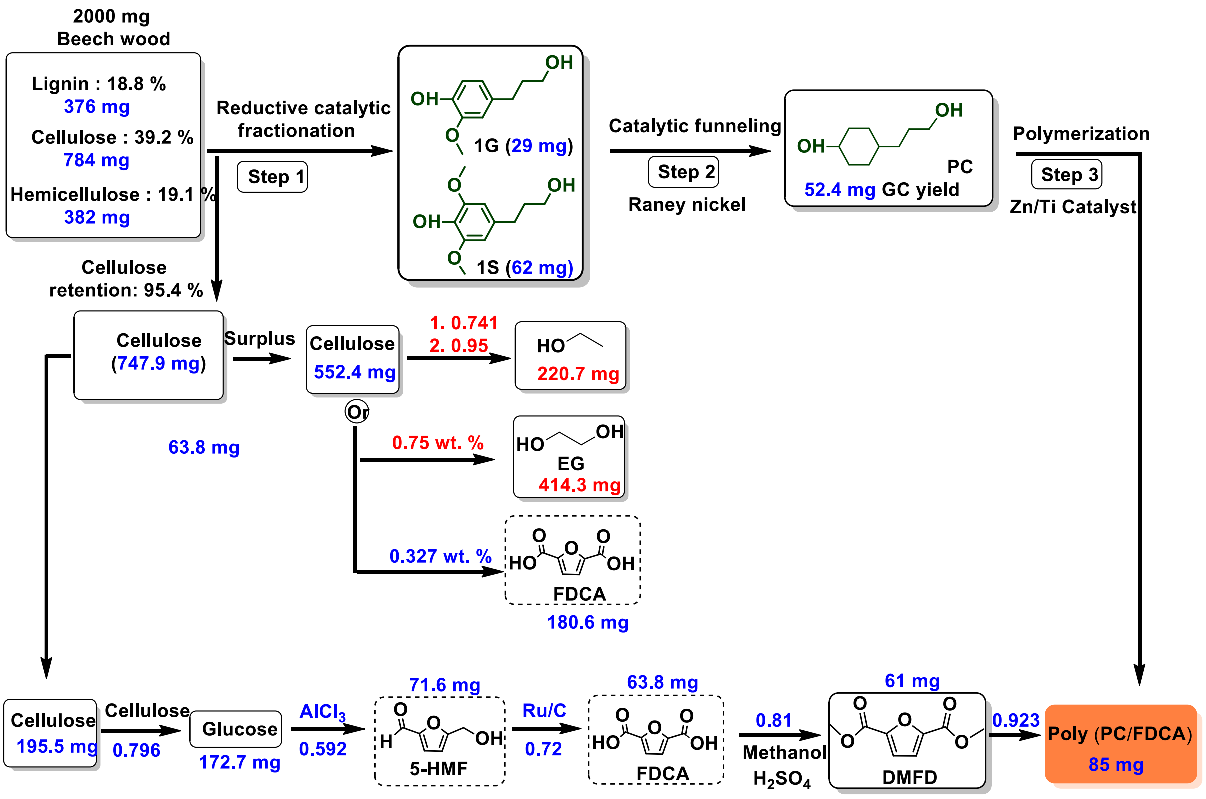

Supplementary Figure 26 A comprehensive proposed strategy for the production of fully wood-based PET analogues. The calculation includes the two-step RCF/catalytic funneling strategy presented in this paper, in combination with literature data for the conversion of the carbohydrate pulp fraction to DMFD, ethanol, furfural and ethylene glycol. Blue number stands for experimental data while red number stands for the data obtained based

on literatures.

Weight determination of cellulose for the production of poly (PC/TPA):

The following calculations are relying on experimental process: Cellulose is subjected to enzymatic hydrolysis to glucose in the yield of 79.6 % over cellulase at 50 °C for 72 h, following by isomerization and dehydration to 5-HMF (59.2 % yield) over $AlCl_3$ catalyst at 120 °C for 4 h. The obtained 5-HMF was oxidized to FDCA in the isolated yield of 72 %, following by methylation into DMFD in the isolated yield of 81 %.

Based on the results obtained above, producing 0.3316 mmol of DMFD for poly (PC/FDCA) theoretically requires the following amount of cellulose:

$$\text{Cellulose (mg)} = 162 \times 0.3316 \div (0.81 \times 0.72 \times 0.592 \times 0.796) = 195.5 \text{ mg} \qquad \text{Eq14}$$

Therefore, the surplus of cellulose is 552.4 mg (552.4 mg = 747.9 – 195.5 mg). Poly (PC/FDCA) can be obtained in 92.3 % yield via two-step melt polymerizations (esterification and polycondensation) using equal molar ratio of diol and diacid methyl ester over TBT catalyst. Poly (PC/FDCA), therefore can be quantified:

$$\text{Poly (PC/FDCA) (mg)} = 0.3316 \times (158 + 184 - 32 \times 2) \times 0.923 = 85 \text{ mg} \qquad \text{Eq15}$$

Considering 100 % yield of polymer synthesis, this accounts for 92.2 mg poly (PC/TPA), quantified as follows:

$$\text{Poly (PC/FDCA) (mg)} = 0.3316 \times (158 + 184 - 32 \times 2) \times 100\,\% = 92.2 \text{ mg} \qquad \text{Eq16}$$

Considering theoretical maximum RCF and 100 % yield for catalytic funneling, as well as 100 % yield of polymer synthesis, this accounts for 163.5 mg poly (PC/FDCA), quantified as follows:

$$\text{Poly (PC/FDCA) (mg)} = 0.5883 \times (158 + 184 - 32 \times 2) \times 1 = 163.5 \text{ mg} \qquad \text{Eq17}$$

Option 1: Determination of ethanol weight from excess carbohydrate pulp.

RCF procedure of beech lignocellulose (2000 mg) using Cu20-PMO towards the production of fully bio-based poly (PC/FDCA) results in approximately 552.4 mg of surplus cellulose. Thus, the ethanol mass would be:

$$\text{Ethanol (mg)} = \frac{552.4}{162.1} \times 0.741 \text{ (cellulose to fructose)} \times 0.95 \times 2 \times 46 \text{ mg} = 220.7 \text{ mg} \qquad \text{Eq 18}$$

Option 2: determination of ethylene glycol weight from excess cellulose.

Thus complete conversion of the surplus cellulose (552.4 mg) would lead to 414.3 mg EG (414.3 mg = 552.4 × 0.75 mg).

Option 3: determination of FDCA weight from excess cellulose.

Thus complete conversion of the surplus cellulose (552.4 mg) would lead to 180.6 mg FDCA (180.6 mg = 552.4 × 0.327 mg).

Supplementary Note 2. Catalytic defunctionalization of model compound 1G to PC

2.1 Establishing of reaction conditions (catalysts, reaction temperature, solvents and pressure) for demethoxylation and hydrogenation of 1G

Supplementary Table 1. Catalytic demethoxylation and hydrogenation of 1G to PC over various commercially available metal catalyst[a].

Catalyst	Conversion[d] (%)	Selectivity[d] (%)							PC yield[d] (%)
		**1**	**2**	**3**	2H	PC	1H	Others	
Raney Ni	84.8	10.8	2.6	1.6	-	84.4	0.5	0.1	71.6
Raney Ni[b]	99.1	11.2	4.0	3.5		81.2	-	0.1	80.5
Ni/γ-Al_2O_3	-	-	-	-	-	-	-	-	-
Ni/SiO_2-Al_2O_3	26.8	45.7	2.4	2.3	-	25.7	-	23.7[c]	6.9
Ni/C	-	-	-	-	-	-	-	-	-
Pd/C	83.4	30.5	2.6	1.8	3.7	11.5	10.2	39.5	9.6
Ru/C	100	51.6	4.2	2.1	11.4	29.0	-	1.7	29.0

[a] General reaction conditions: 1G, (1.1 mmol, 0.2 g), catalyst 1 g , 2 h, 100 °C, 10 bar H_2, 15 mL isopropanol, 20 mg dodecane as internal standard; [b] 0.2 g wet Raney Ni, 160 °C; [c] For Ni/SiO_2-Al_2O_3 catalyst, others contain 20 % selectivity of 3G; [d] Conversion, selectivity and yield determined by GC-FID based on calibration curves and internal standard.

Supplementary Table 2. Catalytic demethoxylation and hydrogenation of **1G** to **PC** over various gas sources[a]

Pressure	Conversion[b] (%)	Selectivity[b] (%)								PC yield[b] (%)
		**1**	**2**	**3**	2H	3H	PC	1H	Others	
1 Bar air	76.7	3.6	17.0	2.1	9.3	12.9	45.5	2.4	13.5	34.9
1 Bar N_2	54.6	10.0	16.5	11.2	2.6	2.5	45.1	3.0	9.0	24.7
30 Bar N_2	92.9	4.5	21.2	13.1	0.6	-	52.7	2.0	5.9	49.0
10 Bar H_2	84.8	10.8	2.6	1.6	-	-	84.4	0.5	0.1	71.6
30 Bar H_2	84.6	15.2	1.6	0.7	-	-	82.0	-	0.5	69.4

[a] General reaction conditions: 1G, (1.1 mmol, 0.2 g), 1 g wet Raney Ni, 2 h, 100 °C, 15 mL solvent, 20 mg dodecane as internal standard; [b] Conversion, selectivity and yield determined by GC-FID based on calibration curves and internal standard.

Supplementary Table 3. Catalytic demethoxylation and hydrogenation of 1G to PC over various solvents [a].

Solvents	Conversion[b] (%)	Selectivity[b] (%)							PC yield[b] (%)
		**1**	**2**	**3**	2H	PC	1H	Others	
Water	21.3	17.1	29.4	-	-	53.4	-	0.4	11.4
Methanol	-	-	-	-	-	-	-	-	-
Ethanol	-	-	-	-	-	-	-	-	-
Butanol	9.3	17.9	-	-	-	74.5	7.6	-	6.9
EtOAc	27.5	16.3	-	-	-	82.5	1.2	-	22.7
2-Me THF	33.5	12.5	-	-	-	83.9	3.4	-	28.2
2-Me BuOH	37.4	14.3	-	-	-	84.7	1.0	-	31.7
THF	56.9	10.1	-	-	-	83.7	5.8	-	47.6
THF	100	11.5	0.6	0.8	-	85.5	1.2	0.4	85.5
Isopropanol	84.8	10.8	2.6	1.6	-	84.4	0.5	0.1	71.6

[a] General reaction conditions: 1G, (1.1 mmol, 0.2 g), 1 g wet Raney Ni, 2 h, 100 °C, 10 bar H_2, 15 mL solvent, 20 mg dodecane as internal standard; [b] Conversion, selectivity and yield determined by GC-FID

based on calibration curves and internal standard;

Supplementary Table 4. Catalytic demethoxylation and hydrogenation of 1G to PC at different reaction temperature [a].

Temperature (°C)	Conversion[b] (%)	Selectivity[b] (%)							PC yield[b] (%)
		**1**	**2**	**3**	2H	PC	1H	Others	
80	40.6	21.3	2.8	-	-	66.7	-	9.2	27.1
90	57.8	14.8	2.5	0.6	-	80.3	1.4	0.4	46.6
100	84.8	11.3	2.5	1.8	-	84.4	-	-	71.6
100-2nd	84.2	14.7	2.9	1.5	-	80.6	-	0.3	67.9
100-3nd	79.9	16.8	1.4	1.9	-	78.3	0.9	0.7	62.6
110	91.8	9.2	1.3	0.9	-	85.5	0.6	2.5	78.5
120	100	13.1	1.8	0.2	-	84.8	-	0.1	84.8

[a] General reaction conditions: 1G, (1.1 mmol, 0.2 g), 1 g wet Raney Ni, 2 h, 80 - 120 °C, 10 bar H_2, 15 mL isopropanol, 20 mg dodecane as internal standard; [b] Conversion, selectivity and yield were determined by GC-FID based on calibration curves and internal standard.

Supplementary Table 5. Catalytic demethoxylation and hydrogenation of 1S to PC diol[a].

Substrates	Tem. [°C]	Time [h]	Pressure [bar]	Conv. [%]	Selectivity[b] (%)					PC Yield[b] (%)
					PC	**1**	**2**	**3**	1G	
1S	100	2	10	27.3	60.2	11.7	2.9	1.5	15.9	16.4
1S	120	2	10	91.0	69.3	7.9	6.7	3.4	2.4	63.3
1S	130	2	10	97.9	72.7	8.8	4.3	3.9	4.4	71.2
1S	130	2	20	98.1	83.3	10.6	1.9	1.6	1.3	81.7
1S	140	2	20	100	84.6	10.7	1.7	2.1	-	84.6

[a] 1 mmol (0.212 g) 1S, 1 g wet Raney Ni, 15 mL isopropanol, 20 mg dodecane as an internal standard; [b] Selectivity and yield values were determined using dodecane as internal standard.

2.2 Proposed reaction network for defunctionalization of 1G

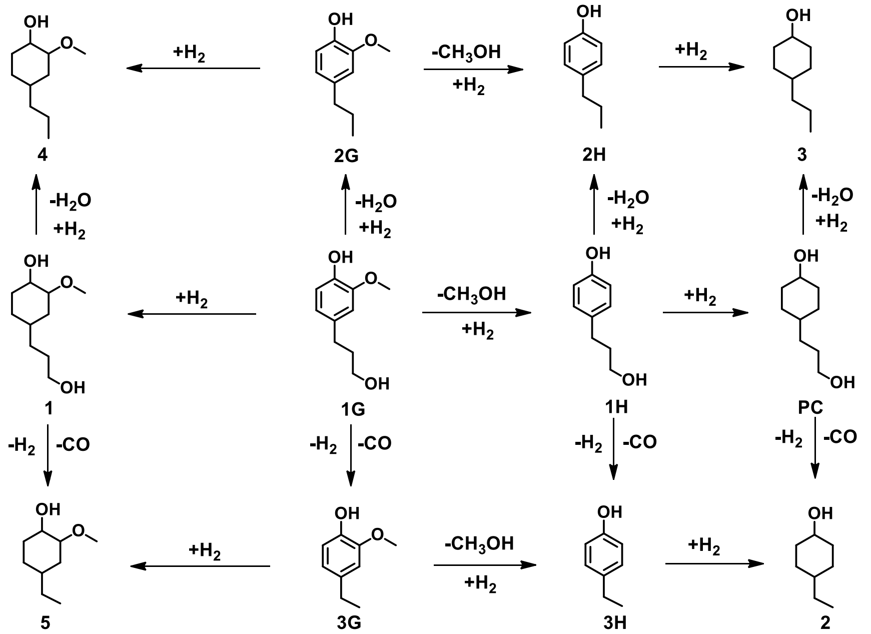

Supplementary Figure 27. Proposed reaction network for the catalytic demethoxylation and hydrogenation of 1G over Raney Ni catalyst using isopropanol as solvent.

2.3 Reaction kinetics for catalytic defunctionalization of 1G, 1H, 1 and PC

Supplementary Table 6. Reaction kinetics for catalytic demethoxylation and hydrogenation of 1G[a].

Time (min)	Conversion[b] (%)	Selectivity (%)[b] PC	1H	**1**	others	Conversion rate[c] (10^{-5} mol·g_{cat}^{-1}·min^{-1})
5	3.6	70.7	4.5	24.5	0.3	1.55
10	7.6	71.1	6.2	22.1	0.6	1.62
20	16.6	76.2	5.5	17.3	1.0	1.78
30	23.5	78.0	4.7	17.1	0.3	1.68
40	33.2	78.1	4.8	17.0	0.1	1.77
50	41.4	76.8	5.5	17.3	0.4	1.77
60	49.7	78.4	4.2	16.9	0.5	1.77
80	58.0	81.0	3.3	15.4	0.3	1.55
100	60.3	82.1	2.2	15.1	0.6	1.29
120	64.5	83.3	1.9	14.2	0.5	1.15

[a] 1.1 mmol 1G, 0.5 g wet Raney Ni, 100 °C, 20 bar H_2, 20 mL isopropanol, 20 mg dodecane as internal standard; [b] Conversion and selectivity determined by GC-FID based on calibration curves and internal standard; [c] Conversion rate (mol·g_{cat}^{-1}·min^{-1}) = converted mol of substrate / (weight catalyst × reaction time).

Supplementary Table 7: Reaction kinetics for catalytic hydrogenation of 1H [a].

Time (min)	Conversion[b] (%)	Selectivity (%)[b] **2**	PC	others	Conversion rate[c] (10^{-5} mol·g_{cat}^{-1}·min^{-1})
5	5.8	0.10	99.8	0.1	3.07
10	9.9	0.11	99.7	0.19	2.62
20	16.2	0.12	99.8	0.08	2.13
30	25.4	0.08	99.5	0.42	2.22
40	40.0	0.09	99.7	0.21	2.63
50	48.7	0.09	99.6	0.31	2.56
60	57.0	0.10	99.4	0.5	2.50
80	67.5	0.08	99.5	0.42	2.22
100	77.7	0.11	99.3	0.59	2.04
120	88.0	0.08	99.4	0.52	1.93

[a] 1.1 mmol 1H, 0.5 g wet Raney Ni, 100 °C, 20 bar H_2, 20 mL isopropanol, 20 mg dodecane as internal standard; [b] Conversion and selectivity determined by GC-FID based on calibration curves and internal standard; [c] Conversion rate (mol·g_{cat}^{-1}·min^{-1}) = converted mol of substrate / (weight catalyst × reaction time).

Supplementary Table 8: Reaction kinetics for catalytic demethoxylation of **1**[a].

Time (min)	Conversion[b] (%)	Selectivity (%)[b] **PC**	others	Conversion rate[c] (10^{-7} mol·g_{cat}^{-1}·min^{-1})
5	0.09	99.8	0.2	4.00
10	0.40	99.9	0.1	8.40
20	0.49	99.8	0.2	5.20
30	0.51	99.6	0.4	3.60
40	0.57	99.8	0.2	3.00
50	0.59	99.9	0.1	2.48
60	0.59	99.7	0.3	2.07
80	0.60	99.6	0.4	1.60
100	0.60	99.8	0.2	1.28
120	0.60	99.8	0.2	1.07

[a] 1.1 mmol **1**, 0.5 g wet Raney Ni, 100 °C, 20 bar H_2, 20 mL isopropanol, 20 mg dodecane as internal standard; [b] Conversion and selectivity determined by GC-FID based on calibration curves and internal standard; [c] Conversion rate (mol·g_{cat}^{-1}·min^{-1}) = converted mol of substrate / weight catalyst × reaction time.

2.4 Kinetic modeling of 1G defunctionalization and hydrogenation

To simplify the calculation, we assume a pseudo-homogeneous power-law model to obtain the apparent kinetic constants. Also, we assume a very high and constant hydrogen concentration in the liquid phase and on the catalyst surface [H] >> [any other compounds]. For the proposed reaction network for 1G transformation to diol PC, the system of ordinary deferential equations will be as following:

$$\frac{d[\mathbf{1G}]}{dt} = -(r_1 + r_3) = -k_{1,app} \times [1H] - k_{3,app} \times [\mathbf{1}] \qquad \text{Eq19}$$

$$\frac{d[1H]}{dt} = r_1 - (r_2 + r_6) = k_{1,app} \times [1G] - k_{2,app} \times [PC] - k_{6,app} \times [\mathbf{2} + other] \qquad \text{Eq20}$$

$$\frac{d[1]}{dt} = r_3 - (r_4 + r_7) = k_{3,app} \times [1G] - k_{4,app} \times [PC] - k_{7,app} \times [\mathbf{2} + other] \qquad \text{Eq21}$$

$$\frac{d[2+other]}{dt} = r_5 + r_6 + r_7 = k_{5,app} \times [1G] + k_{6,app} \times [1H] + k_{7,app} \times [\mathbf{1}] \qquad \text{Eq22}$$

$$\frac{d[PC]}{dt} = r_2 + r_4 = k_{2,app} \times [1H] + k_{4,app} \times [\mathbf{1}] \qquad \text{Eq23}$$

The point when the reactor reached 100 °C was selected as the starting point of the reaction. During experiments, samples were taken directly from the reaction solution using a liquid sampling valve. The collected experimental data (Supplementary Tables S6-S8) were processed & solved numerically; Parameters were estimated by least-square fit of the experimental data using the classic Levenberg-Marquardt algorithm implemented in DynaFit software.[17]

2.5 Fractionation, catalytic funneling and analysis of the crude mixture obtained from RCF of beech lignocellulose

The reductive catalytic fractionation (RCF) of beech lignocellulose over Cu20-PMO catalyst was carried out according to *General procedure A.* Reaction conditions: 2 g beech wood, 0.4 g catalyst, 180 °C, 40 bar H_2, 18 h. RCF of beech wood gave crude aromatic bio-oil (251 mg), which was firstly characterized by GC-FID (Supplementary Figure 28-b) (1G, 31.3 mg, 1S, 65.2 mg, 2S, 21.8 mg, 2G, 5.2 mg and 3S, 4.6 mg), GPC (Supplementary Figure 29) (Mw = 551 g / mol, Mn = 388 g / mol and Mw/Mn = 1.42), 2D HSQC (Supplementary Figure 30), which confirmed the presence of monomers 1G/1S, dimers, trimers, oligomers, lignin residues and sugars. To separate possible oligomeric lignin residues and carbohydrates, which could lead to catalyst deactivation during the subsequent defunctionalization (demethoxylation and hydrogenation) of RCF mixtures, fractionation of crude aromatic bio-oil was performed as demonstrated below. Fractionation of the crude aromatic bio-oil: To the crude aromatic bio-oil, 20 mL of EtOAc was added and it was stirred overnight after which the precipitation of brown solid (25.4 mg) was observed that was separated by centrifugation, decantation and additionally washed with EtOAc (2 × 20 mL). Then the EtOAc insoluble brown solid was characterized by 2D HSQC (Supplementary Figure 31) that confirmed the presence of lignin residues and small amount of sugars. The obtained EtOAc soluble fraction was then washed carefully with saturated $NaHCO_3$ (1 × 5 mL) and brine (2 × 10 mL) to remove possible residue of organic acids and carbohydrates. The aqueous washings were discarded and the EtOAc extracts (201 mg) was characterized by GC-FID (Supplementary Figure

28b overlapped with GC-FID trace of Crude 1), GPC (Mw = 435 g/mol, Mn = 321 g/mol and Mw/Mn = 1.36) (Supplementary Figure 28-c) and 2D HSQC (Supplementary Figure 28-d). The 2D HSQC characterization result confirmed the removal of residual sugars. The EtOAc extracts containing (1G, 29.3 mg, 1S, 61.9 mg, 2S, 19.1 mg, 2G, 4.5 mg and 3S, 4.2 mg) was analysed, then the solvent was removed *in vacuo*. The product was dried in a desiccator *in vacuo* overnight and was used for further demethoxylation and hydrogenation as specified below.

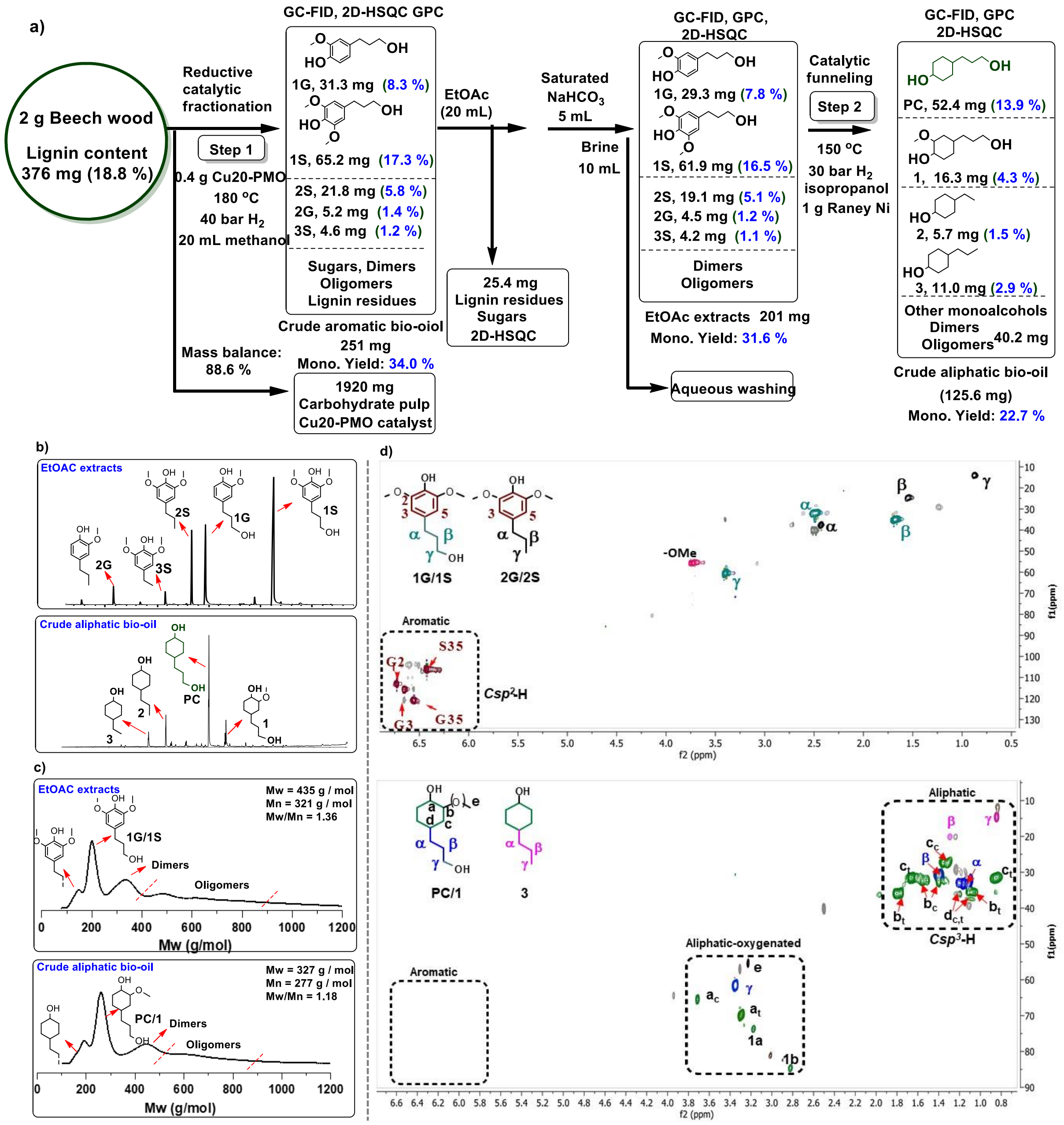

Supplementary Figure 28. A comprehensive catalytic protocol toward the production of PC by RCF of 2 g beech lignocellulose (a) Process scheme showing all catalytic and fractionation steps, with specific composition of the obtained crudes and fractions; (b) Sample GC-FID traces of EtOAc extracts overlapped with GC-FID trace of crude aromatic bio-oil (rich in 1G and 1S) obtained by RCF of lignocellulos and crude aliphatic bio-oil (rich in PC) obtained by catalytic funneling of EtOAc extracts; (c) Gel permeation chromatograms (GPC) of EtOAc extracts and crude aliphatic bio-oil; (d) 2D HSQC spectra of EtOAc extracts (signals clearly assigned to 1G/1S and 2S) and crude aliphatic bio-oil (signals clearly assigned to diol PC and main side product **1** and **3.** Monomers yield

(1G, 1S, 2G, 2S, 3S, PC, **1, 2, 3**) marked in blue color in parentheses was given based on lignin content.

Catalytic funneling of EtOAc extracts: EtOAc extracts (201 mg) was treated using Raney Ni catalyst under the following reaction conditions: (1 g Raney Ni, 150 °C, 20 bar H_2, 3 h, 20 mL isopropanol). After completion of the reaction, the reactor was cooled to room temperature. The Raney Ni was then separated from the solution by centrifugation and decantation and additionally washed with isopropanol (3×20 mL). Then the isopropanol soluble transparent and colorless crude aliphatic bio-oil were combined in a round bottom flask and the solvent was removed in vacuo. The crude aliphatic bio-oil was then characterized by 2D HSQC (Supplementary Figure 28-d), GPC (Mw = 327 g/mol, Mn = 227 g/mol and Mw/Mn = 1.18) (Supplementary Figure 28-c), and GC-FID (PC, 52.4 mg, 1, 16.3 mg, 2, 5.7 mg, 3, 11 mg) (Supplementary Figure 28-b). The results of 2D HSQC and GC-FID characterizations showed that all aromatic monomers, dimers, even oligomers were hydrogenated completely and gave a range of aliphatic alcohols mixture. The mixture rich in PC was then isolated and purified by column chromatography using EtOAc/n-hexane (1:2 to 1:1 to 2:1). PC (44 mg) was obtained from beech wood (42 mg obtained from poplar wood and 20 mg from pine wood as a mixture of cis:trans isomers in 1:2 ratio (GC-FID (Supplementary Figure 28-b)), ^{1}H NMR (Supplementary Figure 32) and ^{13}C NMR (Supplementary Figure 33)).

GPC trace of crude aromatic bio-oil

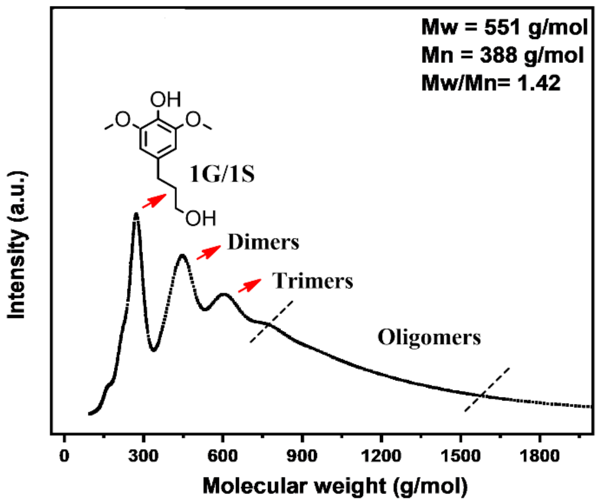

Supplementary Figure 29 GPC trace of crude aromatic bio-oil (2 g beech wood, 0.4 g Cu20-PMO catalyst, 20 ml methanol, 180 °C, 40 bar H_2, 18 h.)

2D HSQC spectra of crude aromatic bio-oil and EtOAc insoluble fraction

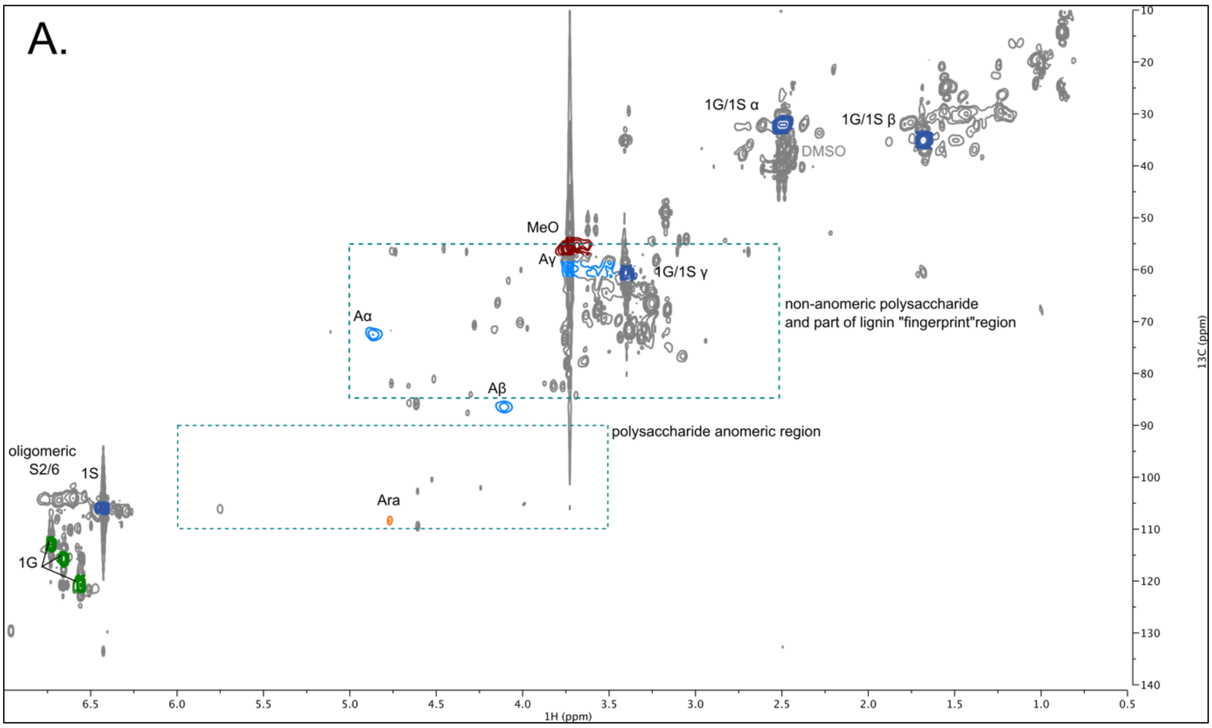

Supplementary Figure 30. 2D HSQC spectrum of crude aromatic bio-oil obtained from RCF of beech wood.

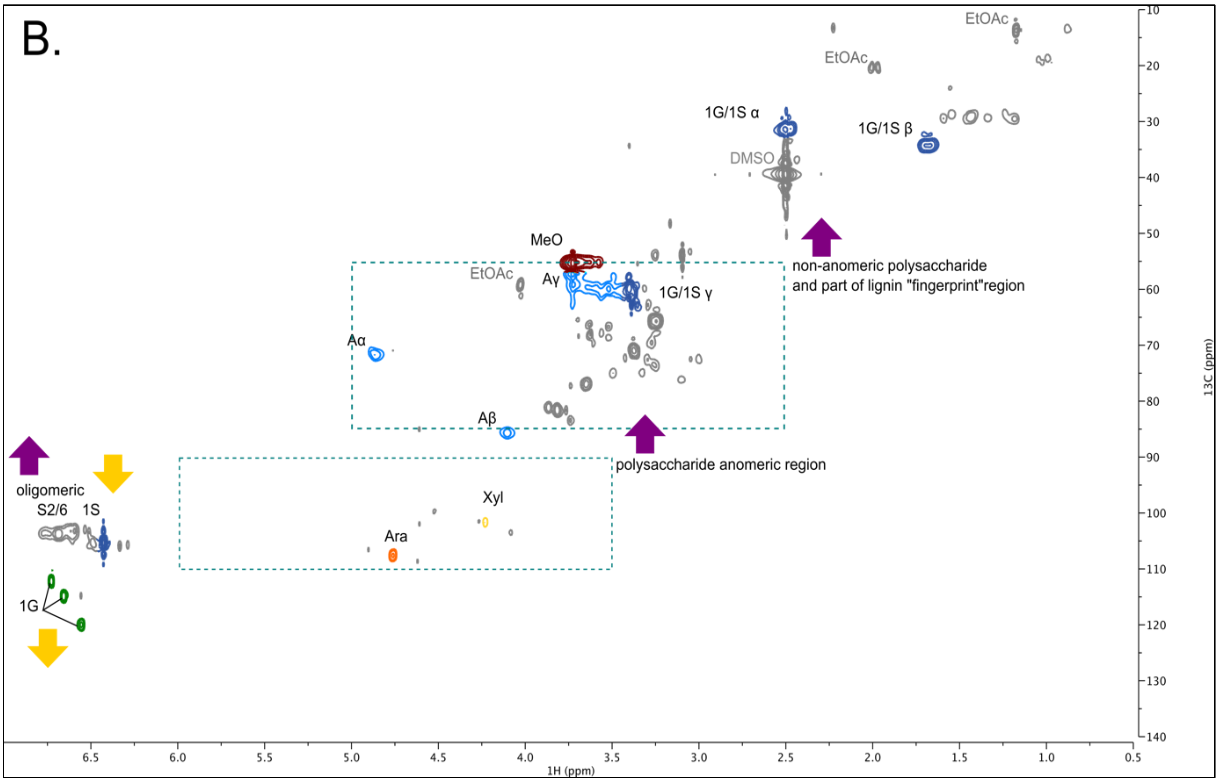

Supplementary Figure 31. 2D HSQC spectrum of EtOAc insoluble fraction obtained from fractionation of crude aromatic bio-oil with EtOAc.

^{1}H NMR and ^{13}C NMR spectra of PC obtained from beech wood.

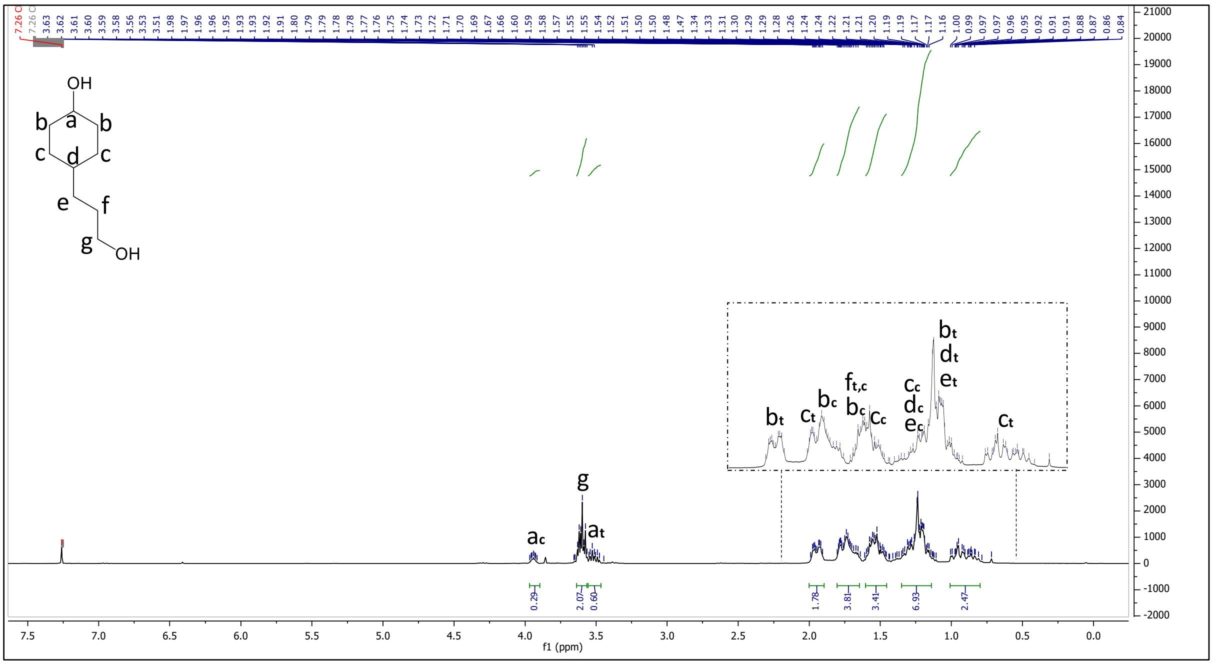

Supplementary Figure 32. ^{1}H NMR spectrum of **PC** isolated from catalytic funneling of EtOAc extracts..

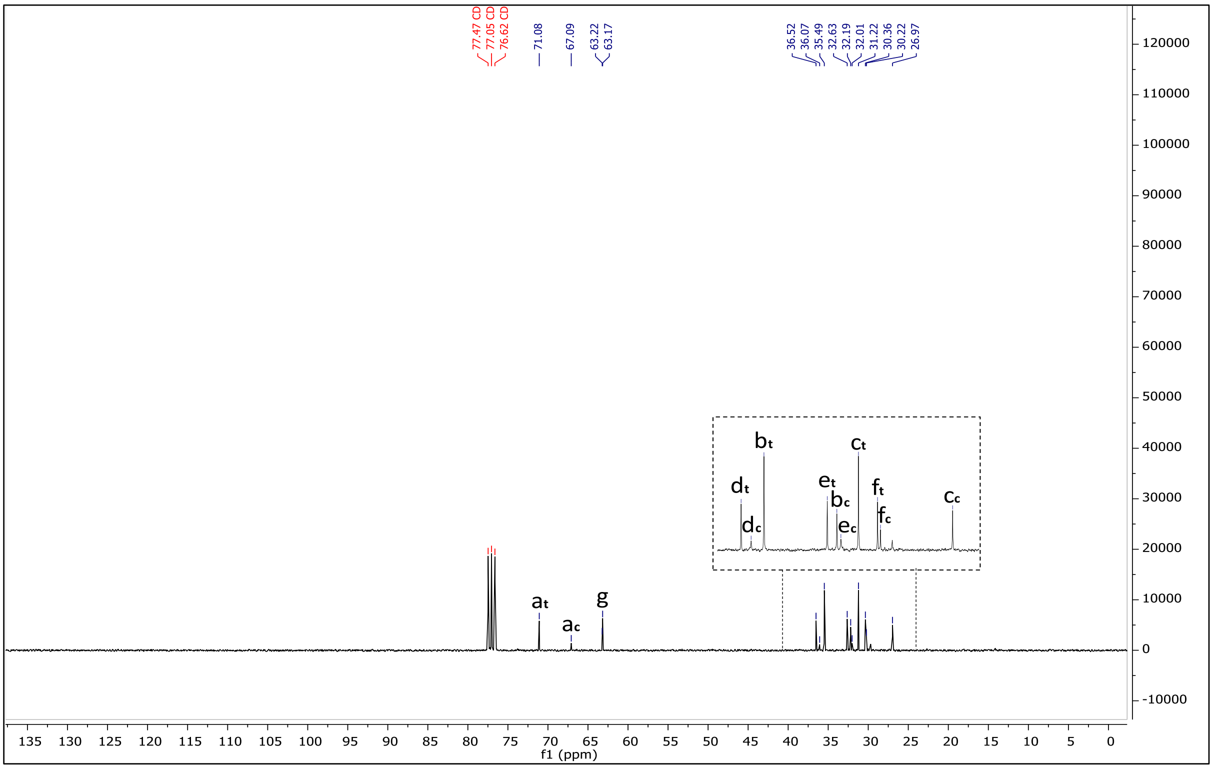

Supplementary Figure 33. ^{13}C NMR spectrum of PC isolated from catalytic funneling of EtOAc extracts

2.6 Upscaling the synthesis procedure of 1G from pine, 1G/1S from poplar and beech wood

Supplementary Table 9. Reproducibility of the established RCF procedure repeated in various scales, including upscaling the synthesis procedure to obtain 1G from pine, and 1G/1S from poplar and beech wood[a].

Substrates	Wood weight (g)	Catalyst weight (g)	Selectivity (%) Weight (mg)						Monomers yield (%)[c]
			3G	3S	2G	2S	1G	1S	
Pine Wood	1	0.2	3.2	0	6.7	0	90.0	0	8.9
			0.7		1.6		23.4		
	2	0.4	4.2	0	7.4	0	88.4	0	8.4
			1.7		3.3		43.2		
	10	2	3.2	0	9.7	0	87.1	0	7.9
			6.3		20.6		202(186)[b]		
Poplar wood	1	0.2	1.9	3.1	3.4	13.0	30.3	48.3	37.3
			1.0	2.0	2.0	9.0	19.4	36	
	2	0.4	0.9	3.8	5.1	13.9	30.0	46.1	37.1
			2.0	4.9	6.0	19	38.1	68	
	10	2	2.2	4.6	6.7	16.1	29.2	41.0	37.4
			12.0	30.3	40	113	190.2	311	
Beech wood	2	0.4	-	3.9	4.8	17.3	26.8	47.9	34.0
				4.6	5.2	21.8	31.3	65.2	
	10	2	-	4.6	5.6	14.3	28.8	46.6	32.9
				26.4	29	88.2	165	310	

[a]. Lignocellulose, Cu20-PMO, methanol, 20 mg 3,5-dimethylphenol as internal standard, 180 °C, 40 bar, 18 h; [b]. Isolated mass in parentheses; [c]. GC yield determined by GC-FID based on calibration curves and internal standard.

Supplementary Table 10. RCF of beech wood over different commercial catalysts[a].

Entry	Catalyst	Selectivity (%) Weight (mg)						Monomers yield (%)[b]
		3G	3S	2G	2S	1G	1S	
1	Cu20-PMO	0	3.9	4.8	17.3	26.8	47.9	34.0
		0	4.6	5.2	21.8	31.3	65.2	
2	Pd/C	0	1.7	0	4.1	34.3	59.7	32.9
		0	2.1	0	4.9	38.6	78.3	

[a]. 2 g beech wood, 200 mg catalyst, methanol, 20 mg 3,5-dimethylphenol as internal standard, 180 °C, 40 bar, 18 h; [b]. GC yield determined by GC-FID was based on calibration curves and internal standard.

Supplementary Note 3. Synthesis of poly (PC/TPA) and poly (PC/FDCA) using model compound PC and its isomers

3.1 Synthesis, analysis and characterization of poly (PC/TPA)

Synthesis of poly (PC/TPA): The two-step melt polymerizations (esterification and polycondensation) were performed according to General procedure C using equal molar ratio of PC and cellulose-derived DMTA over $Zn(OAc)_2$ catalyst[18]. Briefly, a 100 mL three-neck flask, equipped with a magnetic stirrer and reflux condenser, was charged with 0.53 g (3.35 mmol) of PC diol, 0.66 g of DMTA (3.35 mmol) and 1 mol % (0.0057 g) $Zn(OAc)_2$ catalyst. The esterification reaction was performed at 190 °C for 1 h under nitrogen flow. Then the reaction temperature was increased to 230 °C, the pressure was reduced to 1 mPa using an oil pulp for 1 h and 3 h, respectively. After that, the reaction mixture was cooled down to room temperature and the pressure was returned to atmospheric pressure by introducing nitrogen. Finally, 0.734 g and 0.710 g of white and transparent solid poly (PC/TPA) was obtained in 75.2 % (at vacuum for 1 h) and 72.1% (at vacuum for 3 h) yield, respectively. The procedure for synthesis of poly (PC/TPA) was shown in Supplementary Figure 34. The synthesized poly (PC/TPA) was structurally characterized by ^{1}H NMR (Supplementary Figure 42), ^{13}C NMR (Supplementary Figure 43), 2D HSQC (Supplementary Figure 44), FTIR (Supplementary Figure 68) and GPC (Supplementary Figure 50). Thermal properties of the synthesized (PC/TPA) were characterized by TGA (Supplementary Figure 49) and DSC (Supplementary Figure 48). Then poly (PC/TPA)-3h was dissolved in chloroform (5 mL) and precipitated with methanol (50 mL) to obtain purified poly (PC/TPA) solid (0.49 g) in a yield of 50.8 %. The purified poly (PC/TPA)-3h was characterized by GPC (Supplementary Figure 56), TGA (Supplementary Figure 55) and DSC (Supplementary Figure 54).

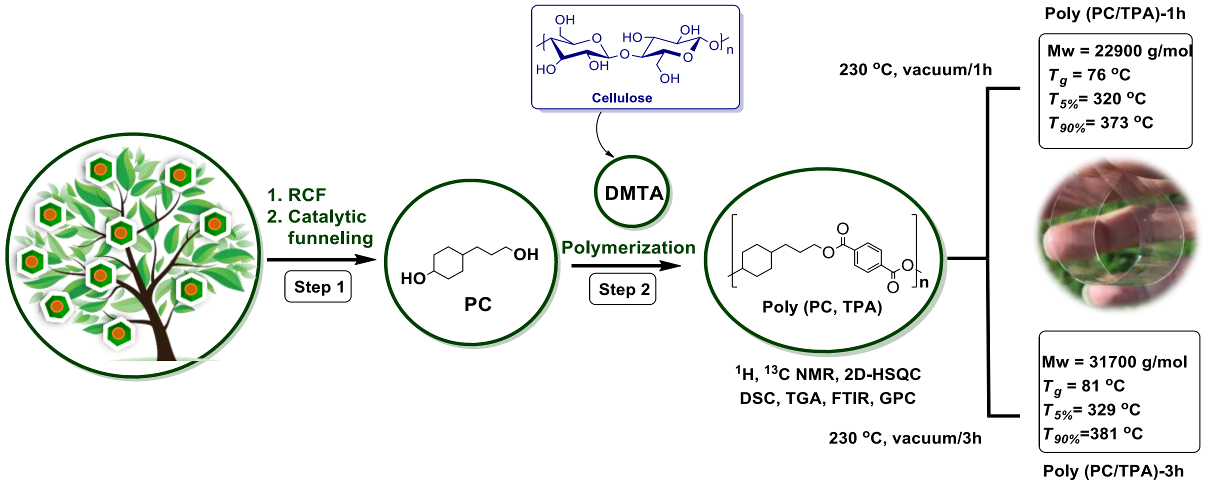

Supplementary Figure 34. The copolymerization of PC with cellulose-derived DMTA for synthesis of poly (PC/TPA). Reaction conditions: 0.53 g (3.35 mmol) PC diol, 0.66 g (3.35 mmol) DMTA, 1 mol % (5.7 mg) $Zn(OAc)_2$, 190 °C/N_2 for 1 h, 230 °C under vacuum for 1 h or 3 h.

Synthesis of poly (PC_{cis}/TPA) and poly (PC_{trans}/TPA): The two-step melt polymerizations (esterification and polycondensation) were performed according to General procedure C using equal molar ratio of PC_{cis} or PC_{trans} (3.35 mmol, 0.53 g) and cellulose-derived DMTA (3.35 mmol, 0.66 g) over 1 mol% (0.0057 g) $Zn(OAc)_2$ catalyst, as described above for synthesis of poly (PC/TPA). The esterification reaction and polycondensation were performed at 190 °C for 1 h under nitrogen flow and at 230 °C for 1 h under reduced vacuum (1 mPa) using an oil pulp, respectively. Finally, 0.82 g and 0.52 g of white and transparent solid poly (PC_{cis}/TPA) and poly (PC_{trans}/TPA) was obtained in 84 % yield and 53 % yield, respectively. The synthesized poly (PC_{cis}/TPA) and (PC_{trans}/TPA) were structurally characterized by ^{1}H NMR (Supplementary Figure 35 and Supplementary Figure 38), ^{13}C NMR (Supplementary Figure 36 and Supplementary Figure 39), 2D HSQC (Supplementary Figure 37 and Supplementary Figure 40), and GPC (Supplementary Figure 52). Thermal properties of the synthesized (PC_{cis}/TPA) and (PC_{trans}/TPA) were characterized by TGA (Supplementary Figure 51) and DSC (Supplementary Figure 50).

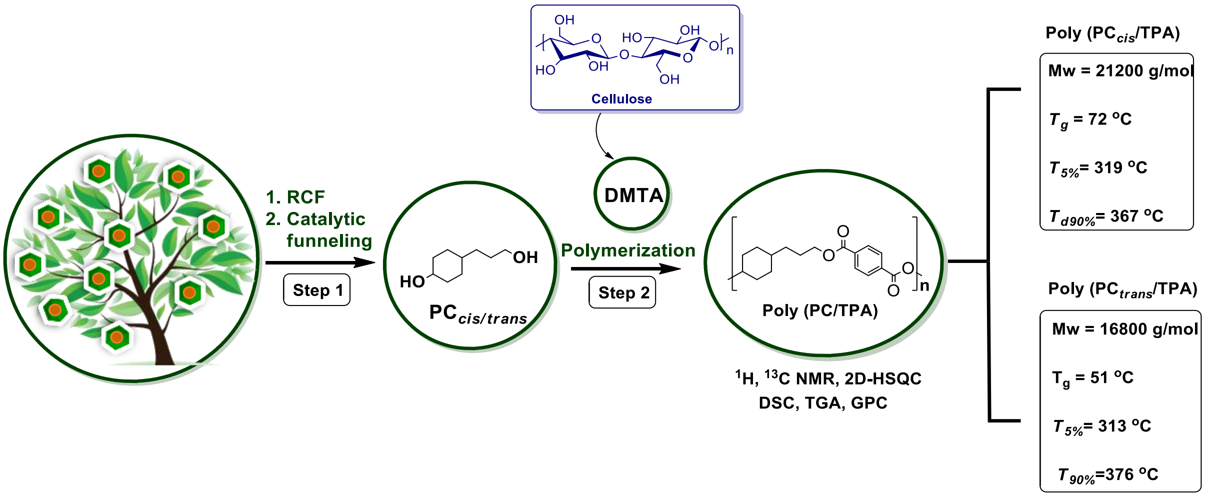

Supplementary Figure 35. The copolymerization of PC_{cis} or with PC_{trans} DMTA for synthesis of poly (PC_{cis}/TPA) or poly (PC_{trans}/ TPA); Reaction conditions: 0.53 g (3.35 mmol) PC diol, 0.66 g (3.35 mmol) DMTA, 1 mol % (0.0057 g) $Zn(OAc)_2$, 190 °C/N_2 for 1 h, 230 °C under vacuum for 1 h or 3 h.

^{1}H NMR, ^{13}C NMR and 2D HSQC spectra of poly (PC_{cis}/TPA), poly (PC_{trans}/TPA) and poly (PC/TPA)

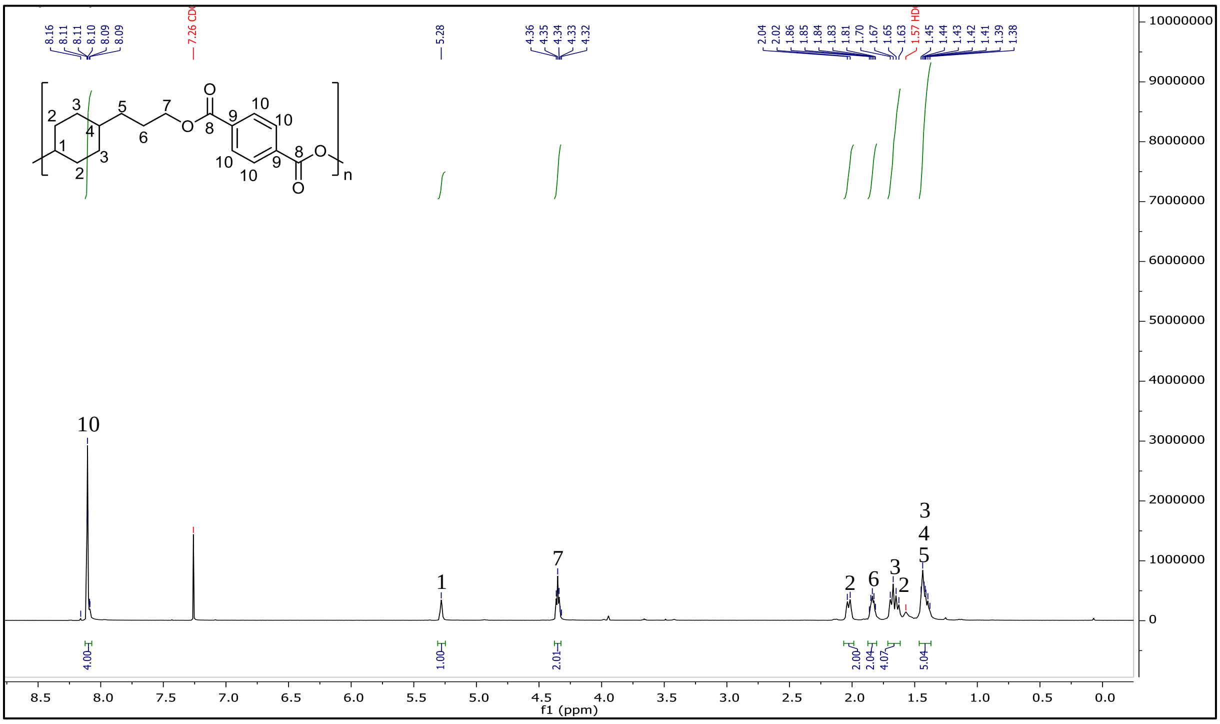

Supplementary Figure 36. ^{1}H NMR spectrum of poly (PC/TPA).

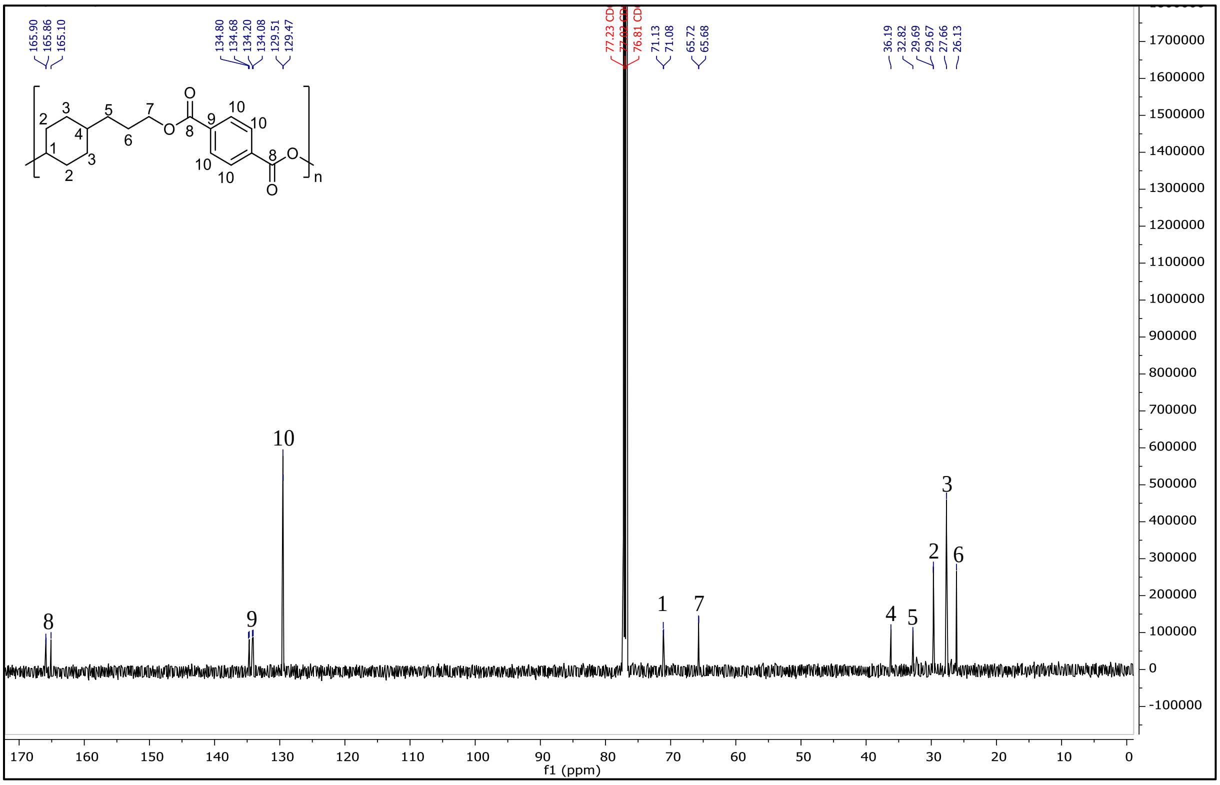

Supplementary Figure 37. ^{13}C NMR spectrum of poly (PC_{cis}/TPA).

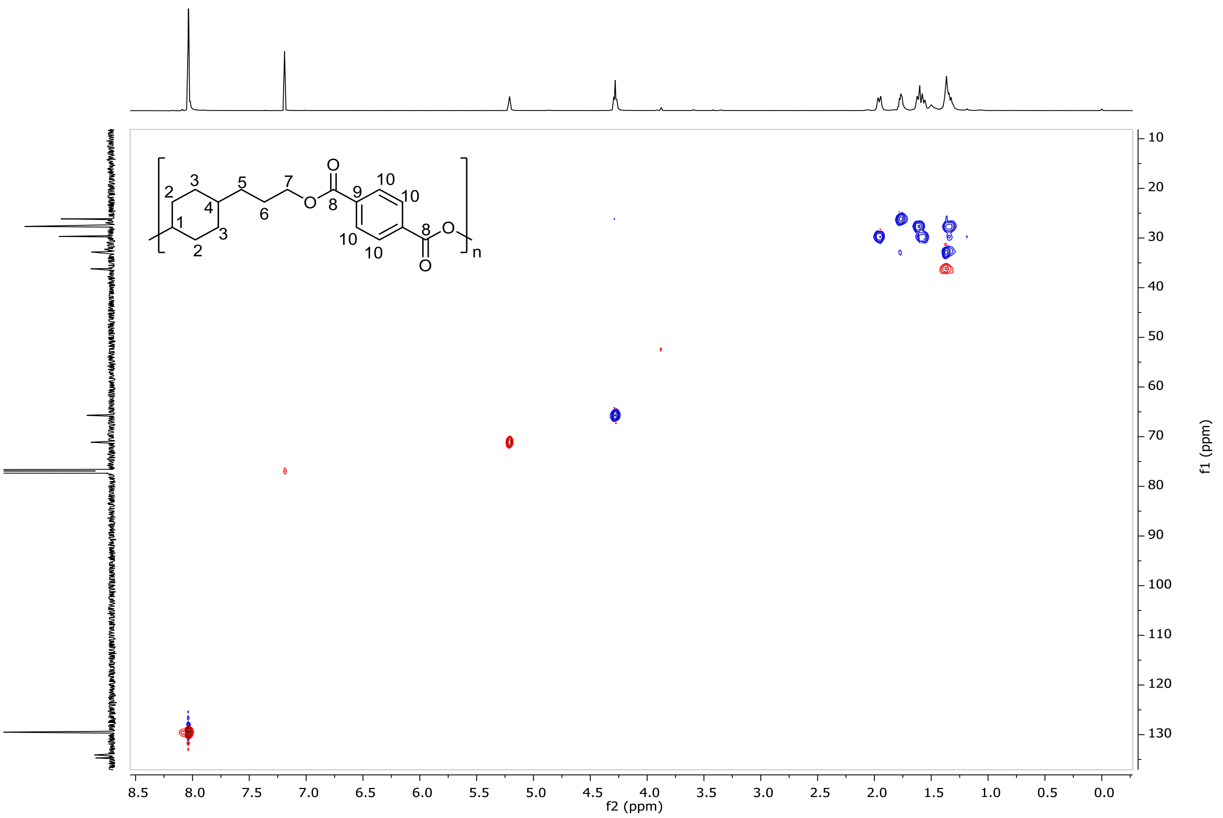

Supplementary Figure 38. 2D HSQC spectrum of poly (PC_{cis}/TPA).

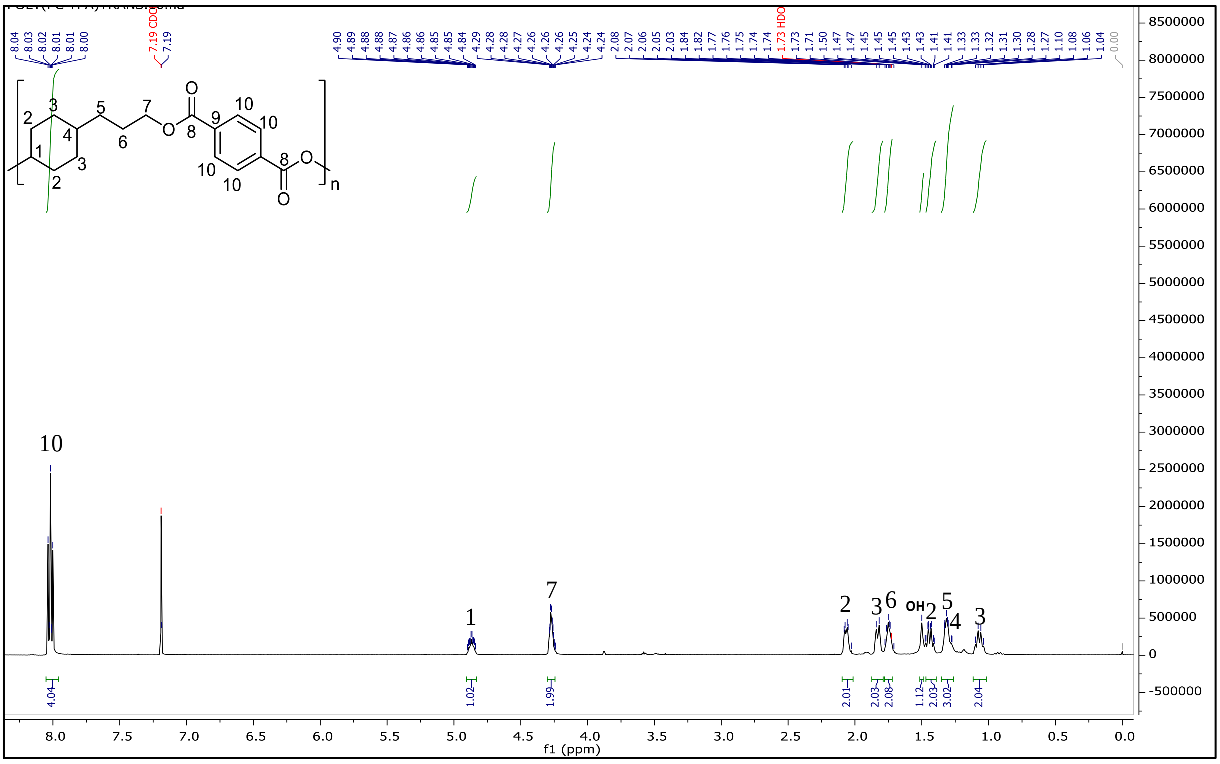

Supplementary Figure 39. ^1H NMR spectrum of poly (PC_{trans}/TPA).

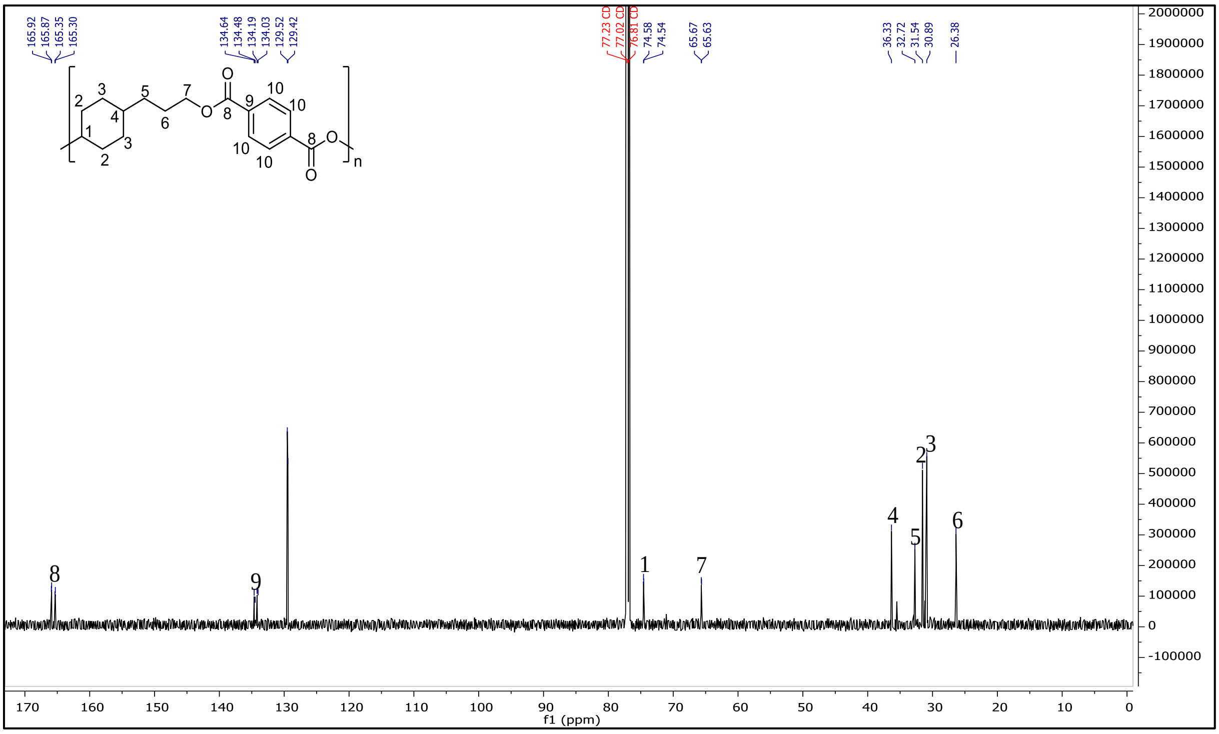

Supplementary Figure 40. ^{13}C NMR spectrum of poly (PC_{trans}/TPA).

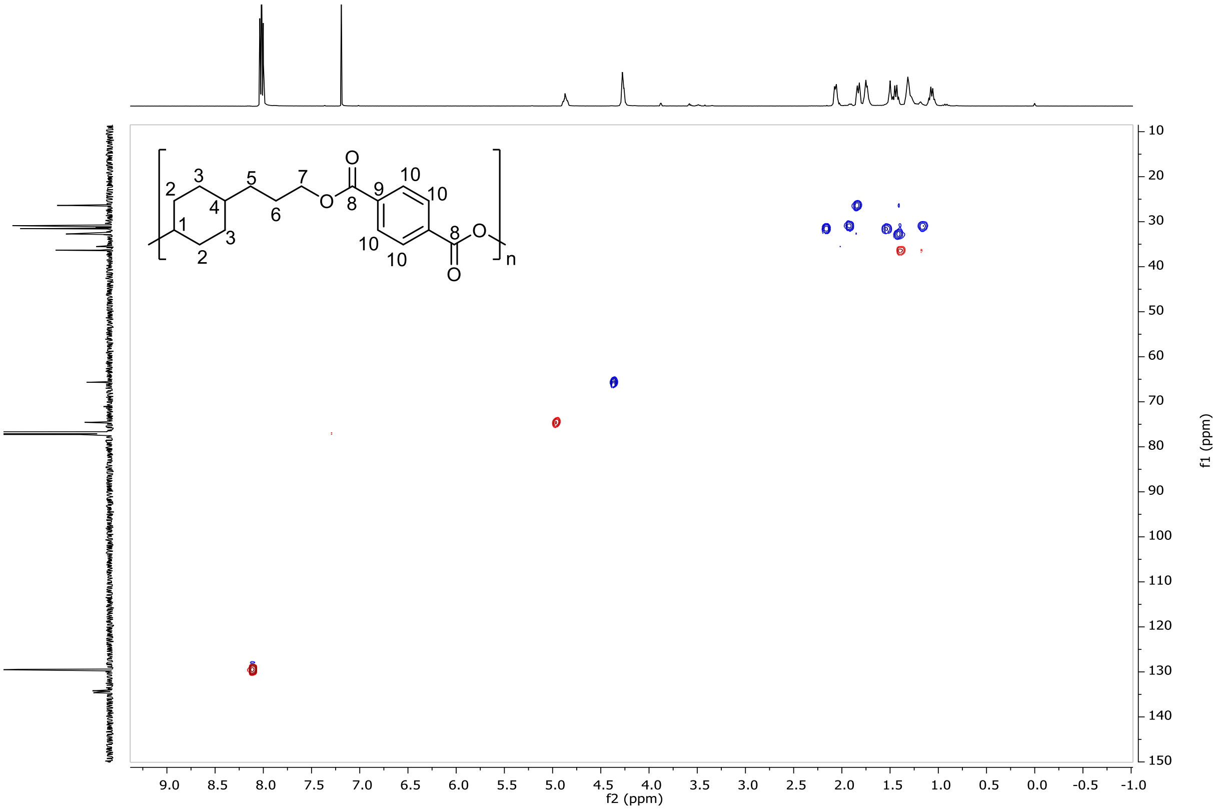

Supplementary Figure 41. 2D HSQC spectrum of poly (PC_{trans}/TPA).

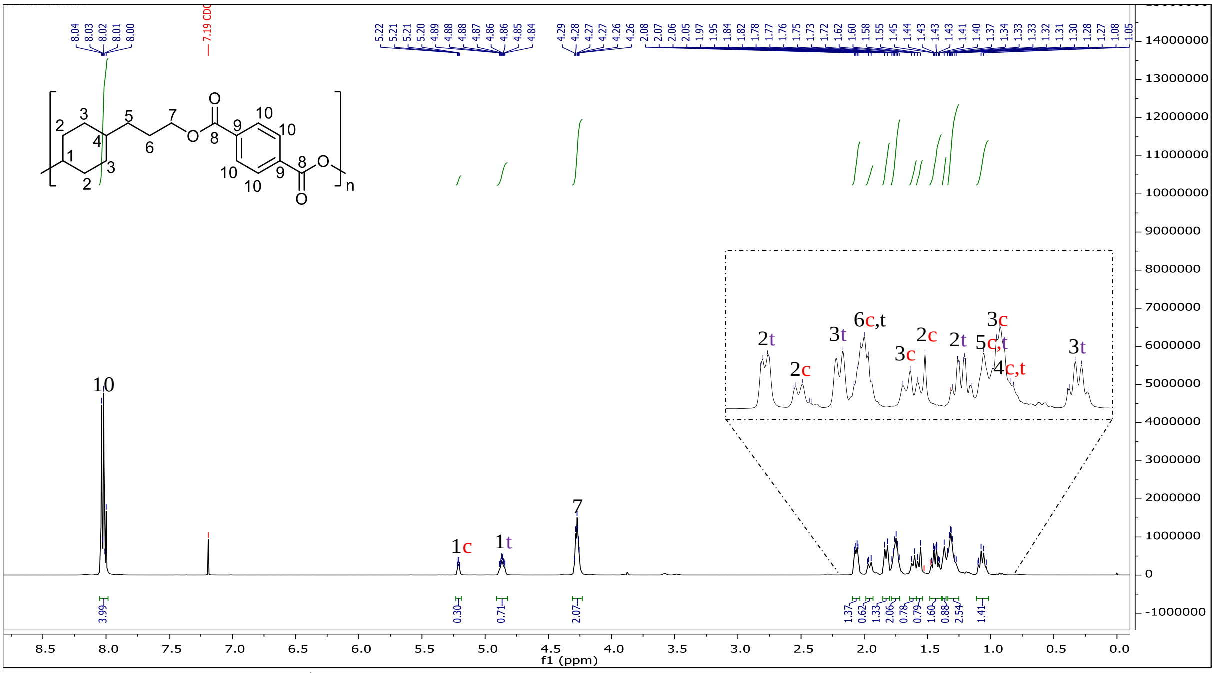

Supplementary Figure 42. ^{1}H NMR spectrum of poly (PC/TPA).

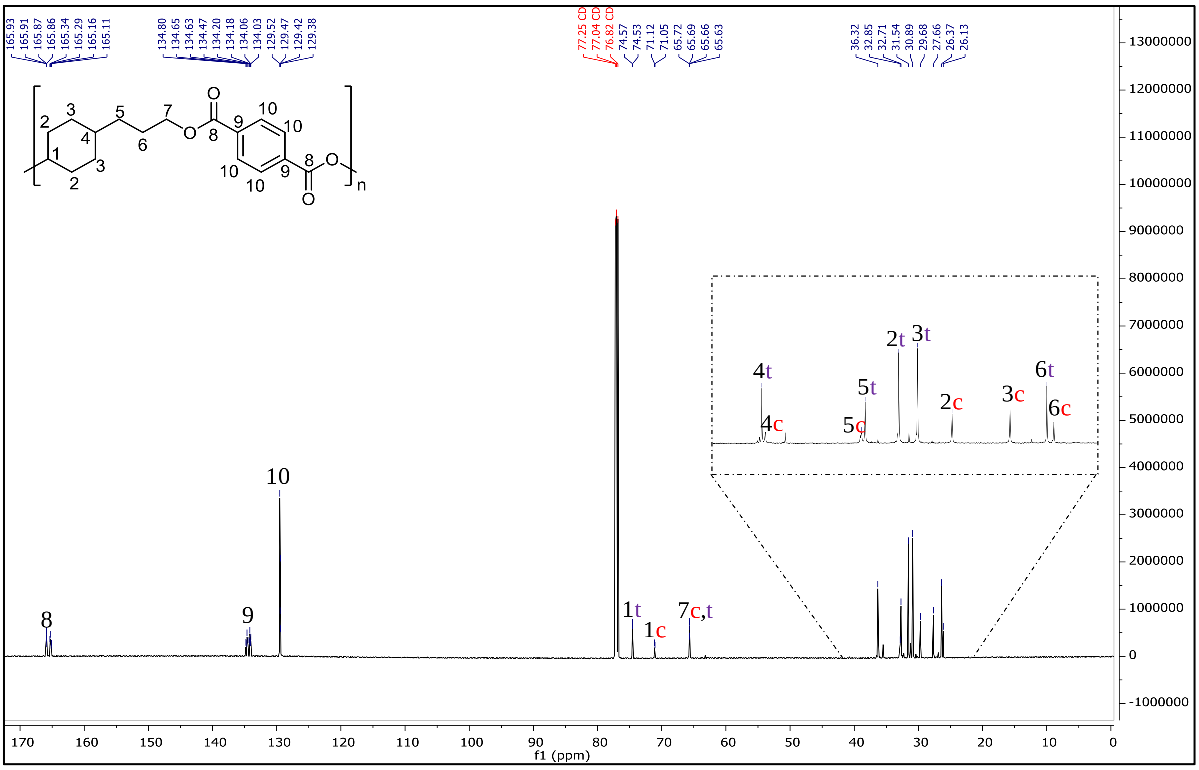

Supplementary Figure 43. ^{13}C NMR spectrum of poly (PC/TPA).

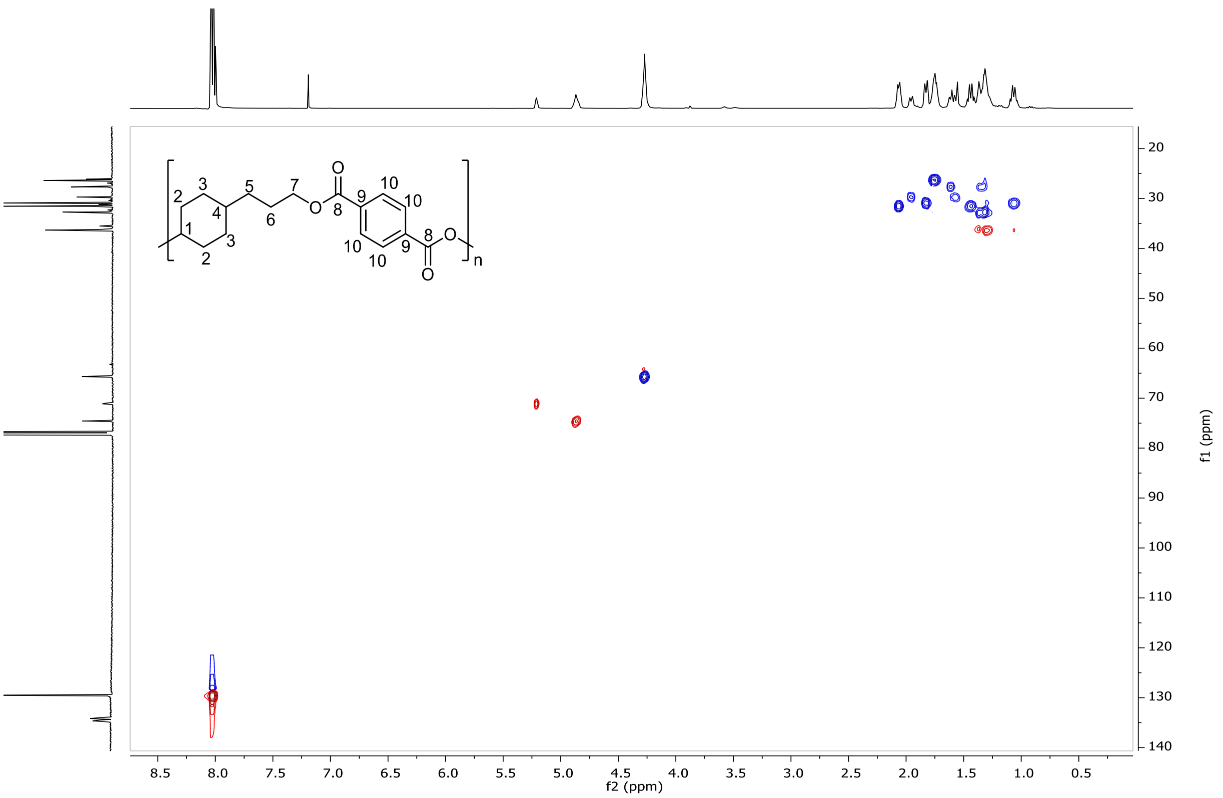

Supplementary Figure 44. 2D HSQC spectrum of poly (PC/TPA).

Detailed structural analysis of poly (PC/TPA)

Structural characterization of poly (PC/TPA) was performed by ^{1}H, ^{13}C NMR, 2D NMR (Supplementary Figures 42-44), FT-IR (Supplementary Figure 68) spectroscopy, as discussed in detail below. Featuring both an aliphatic primary and secondary alcohol moiety, diol PC is asymmetric in nature and this leads to different reactivity and unit connectivity for the prepared polymers. As illustrated in Supplementary Figure 45, the PC unit can form an ester linkage at each end of the molecule, hence three distinct dyads, namely head-to-tail (H-T), head-to-head (H-H), and tail-to-tail (T-T) are formed. Furthermore, PC is obtained upon catalytic treatment as a 1:2 mixture of cis: trans isomers, and this may further influence the properties of the obtained polymers and renders structural characterization non-trivial. Therefore, to assess these influences, and to further aid assignment of the NMR signals, we have separately synthesized poly (PC/TPA) starting from pure trans and pure cis PC as well as a mixture (1:2 of cis: trans) of PC isomers and compared these polymers at the end of the characterization for verification.

Considering the characterization of poly (PC/TPA), prepared by polycondensation of PC with DMTA, the following method was adopted: We start with the analysis of poly (PC/TPA), where PC is a mixture of cis and trans isomers (ratio of 1: 2). We first assigned the main chain-sequence of the polymer by ^{1}H and ^{13}C NMR spectra. Combining information obtained from 2D HSQC, and 2D HMBC experiments performed on poly (PC/TPA) allowed to assign signals where obtained spectral data was in accord with the proposed structure of the polymer. To understand the polymer dyad structure, we first focused on the

analysis of ^{13}C NMR (Supplementary Figure 45) in which three groups of carbonyl carbon (C8) signals were distinguishable. According to literature data, the high field signals of C8 at 165.24 ppm (C8 cis) and 165.42 ppm (C8 trans) were assigned to H-H type[19], the low field C8 signals (cis and trans overlapped) at 166.05 ppm to T-T type structure[20], and the signals in-between (165.28, 165.48, and 165.97 ppm) to mixed (H-T, T-H) type connectivity units. The signal assignment was further confirmed by 2D ^{1}H-^{13}C HMBC (Supplementary Figure 46), where protons H1, correlating with C8 at 165.2 – 165.5 ppm and at 165.95-166.05 ppm, respectively displayed a H type bonding, while proton H7 correlating with C8 at 165.9 – 166.1 ppm was assigned to a T type bonding.

The proposed structure assignment was further supported by comparing the spectral data of poly (PC/TPA) with poly (PC_{trans}/TPA) and poly (PC_{cis}/TPA) separately made from pure trans or cis PC, respectively (Supplementary Figure 47). Overall, the C8 of these three polymers demonstrate the subdivision of each bonding type into two types of signals. For example, the group of signals assigned as T type bonding of poly (PC/TPA) at 165.9 – 166.1 ppm display two subgroups: 165.9–166.0 ppm and 166.0–166.1 ppm. These differences in chemical shifts were assigned to different chemical surrounding of the other ester bond. For example, the higher field group of signals at 165.9 – 166.0 ppm is T type bonding with H type bonding on the other end of the terephthalate. The lower field group of signals at 166.0–166.1 ppm originates from terephthalate with the same T and T type of bonding on both sides. The signals of C8 were assigned accordingly to T-T, T-H and H-H type of dyads, which displayed a random distribution: [H-H] = [T-T] = 0.25 and [H-T] = 0.50, as quantified by integration of quantitative ^{13}C NMR spectra (Supplementary Figure 45).

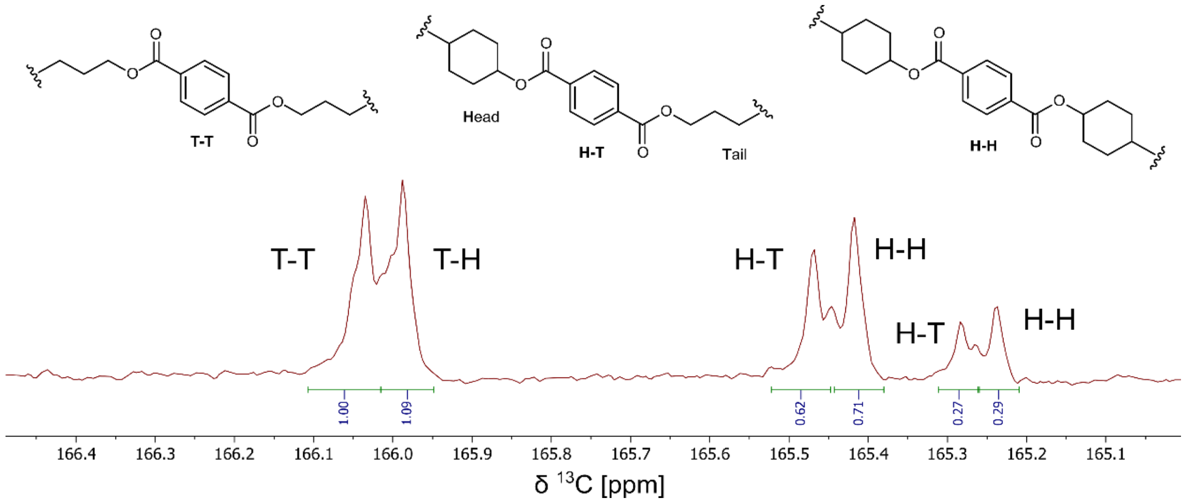

Supplementary Figure 45. The identification spectrum of carbonyl carbon signals (C8) of poly (PC/TPA).

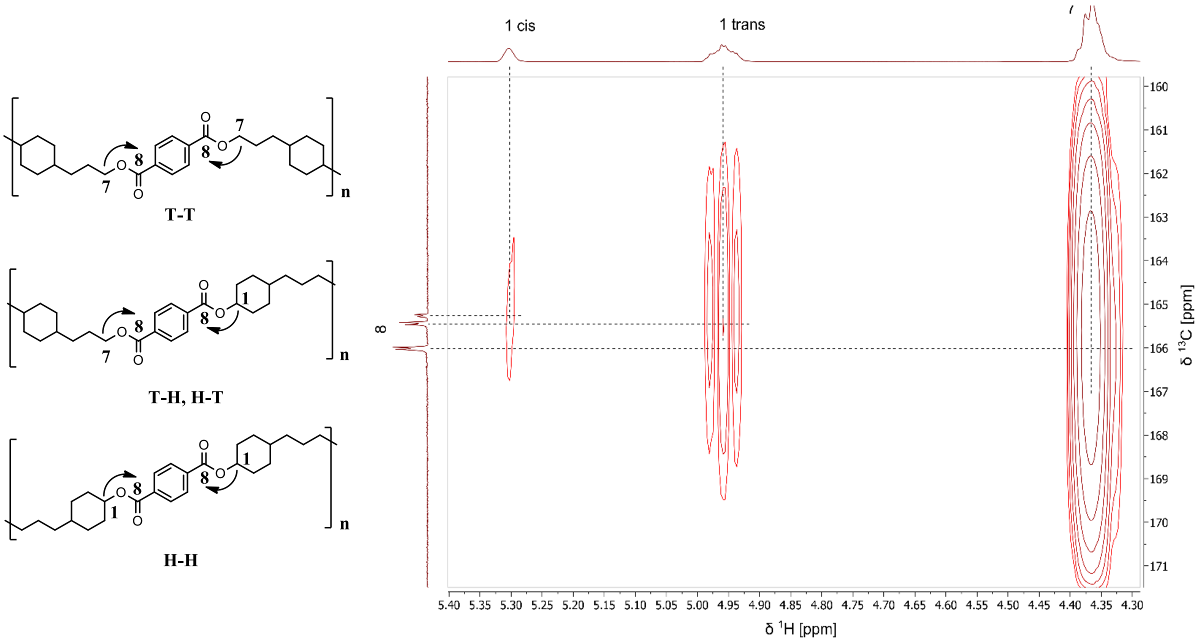

Supplementary Figure 46. Selected area of 2D HMBC spectrum of poly (PC/TPA).

$$\sum_{T-T} = 1.00 \qquad \text{Eq24}$$

$$\sum_{H-T} = 1.09 + 0.62 + 0.27 = 1.98 \qquad \text{Eq25}$$

$$\sum_{H-H} = 0.71 + 0.29 = 1.00 \qquad \text{Eq26}$$

$$\sum_{all} = 1.00 + 1.00 + 1.98 = 3.98 \qquad \text{Eq27}$$

$$\chi_{T-T} = \frac{1.00}{3.98} \times 100\% = 25\% \qquad \text{Eq28}$$

$$\chi_{H-H} = \frac{1.00}{3.98} \times 100\% = 25\% \qquad \text{Eq29}$$

$$\chi_{H-T} = \frac{1.98}{3.98} \times 100\% = 50\% \qquad \text{Eq30}$$

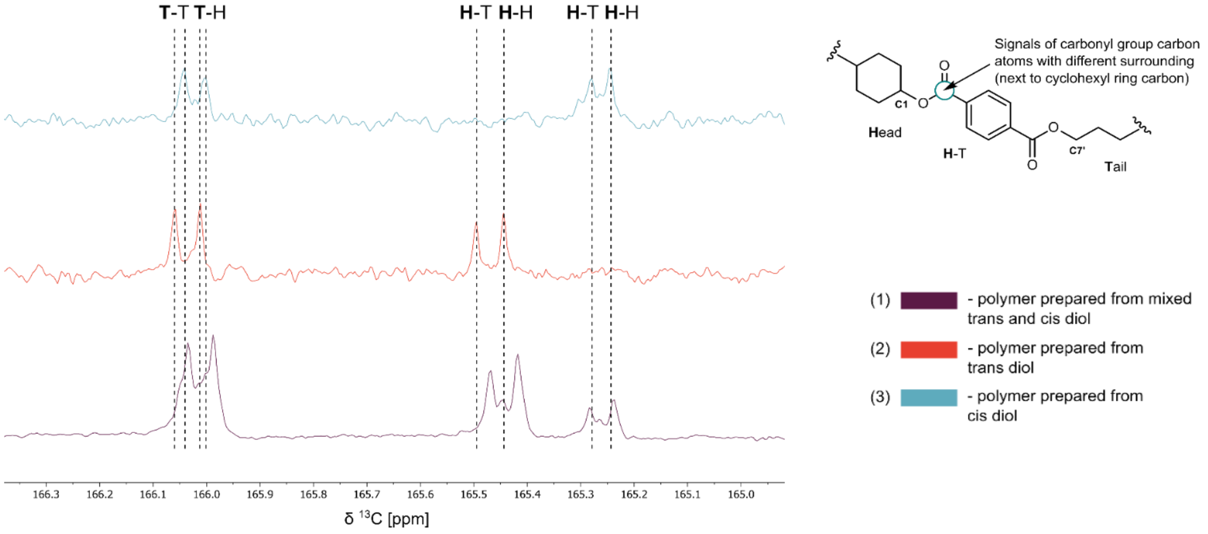

Supplementary Figure 47 The identification spectrum of carbonyl carbon signals (C8) of poly (PC/TPA), poly (PC_{trans}/TPA) and poly (PC_{cis}/TPA).

3.1.1 DSC, TGA and GPC of poly (PC_{cis}/TPA), poly (PC_{trans}/ TPA) and poly (PC/TPA)

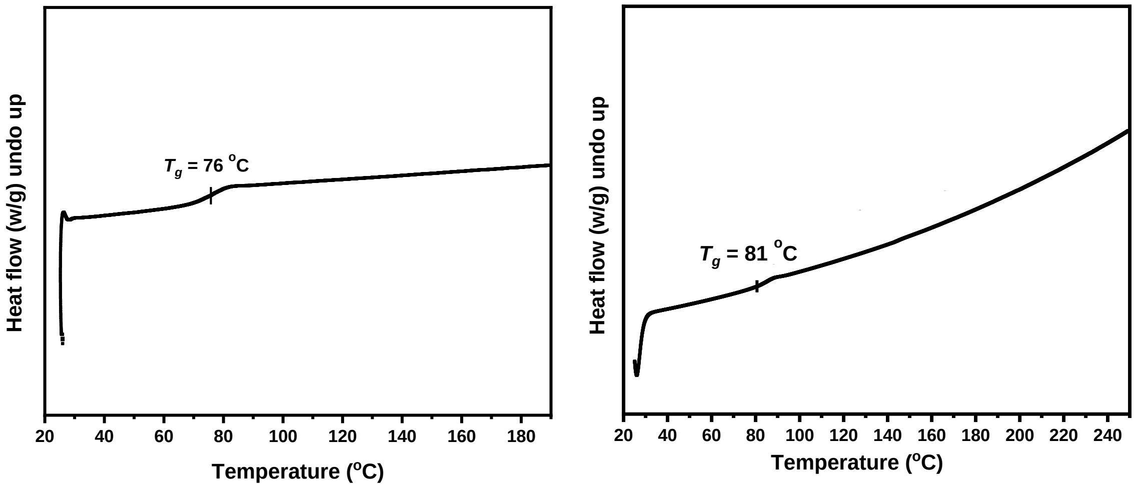

Supplementary Figure 48. DSC thermograms of poly (PC/TPA) at 230 °C under vacuum for 1h (left) and for 3h (right).

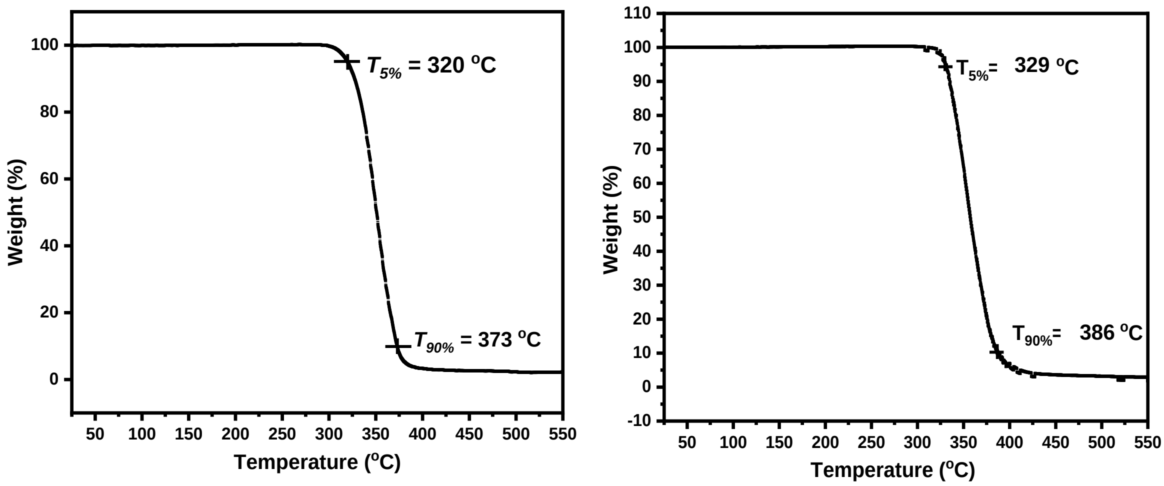

Supplementary Figure 49. TGA plots of poly (**PC/TPA**) at 230 °C under vacuum for 1h (left) and for 3h (right).

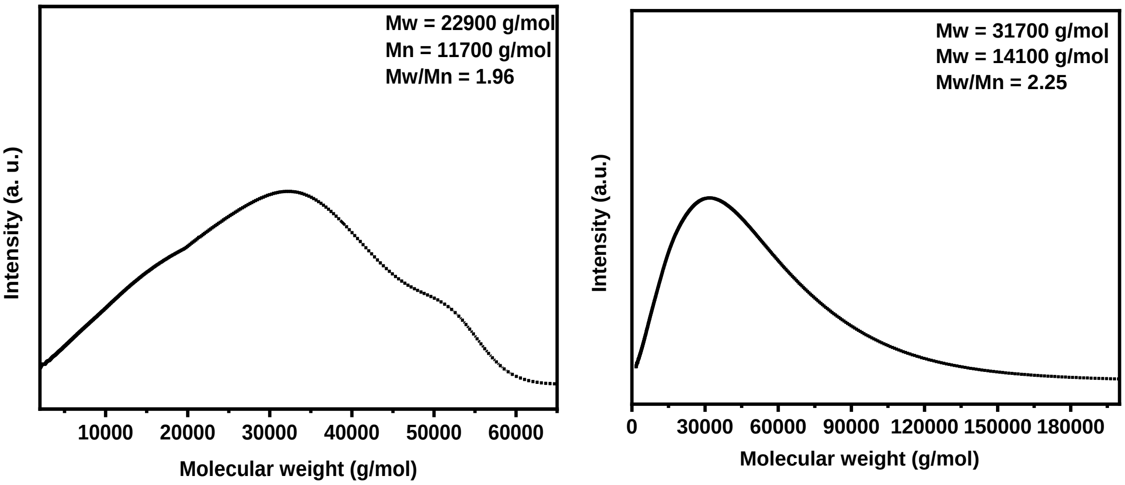

Supplementary Figure 50. GPC traces of poly (PC/TPA) at 230 °C under vacuum for 1h (left) and for 3h (right).

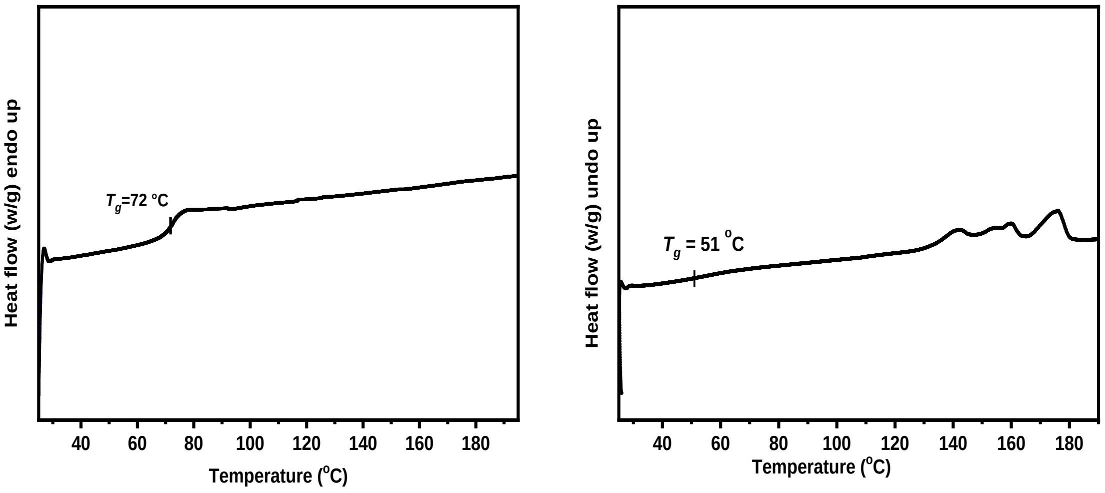

Supplementary Figure 51. DSC thermograms of poly (PC_{cis}/TPA) (left) and poly (PC_{trans}/TPA) (right)

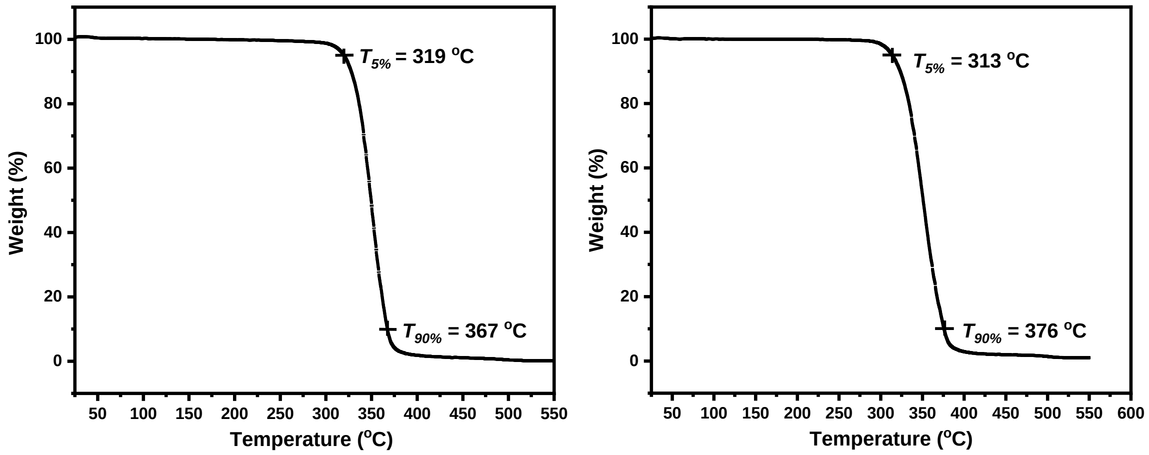

Supplementary Figure 52. TGA plots of poly poly (PC_{cis}/TPA) (left) and poly (PC_{trans}/TPA) (right).

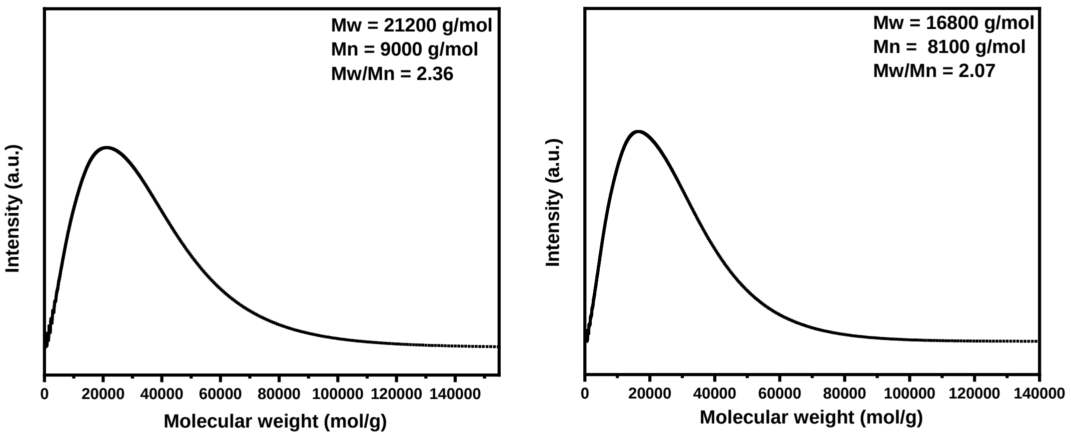

Supplementary Figure 53. GPC traces of poly (PC_{cis}/TPA) (left) and poly (PC_{trans}/TPA) (right).

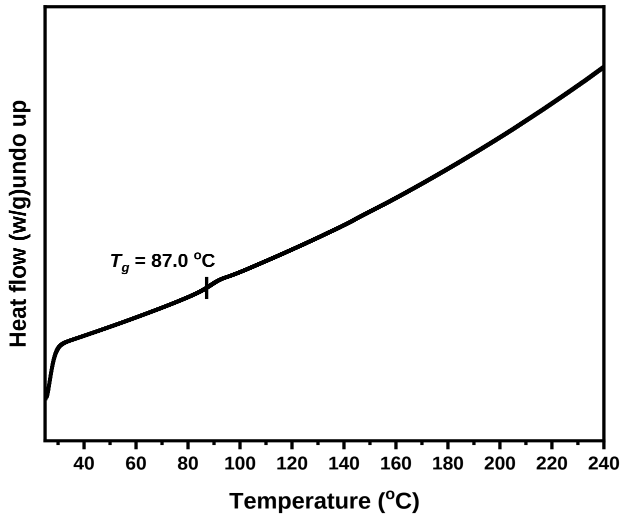

Supplementary Figure 54. DSC thermogram of purified poly (PC/TPA)-3h

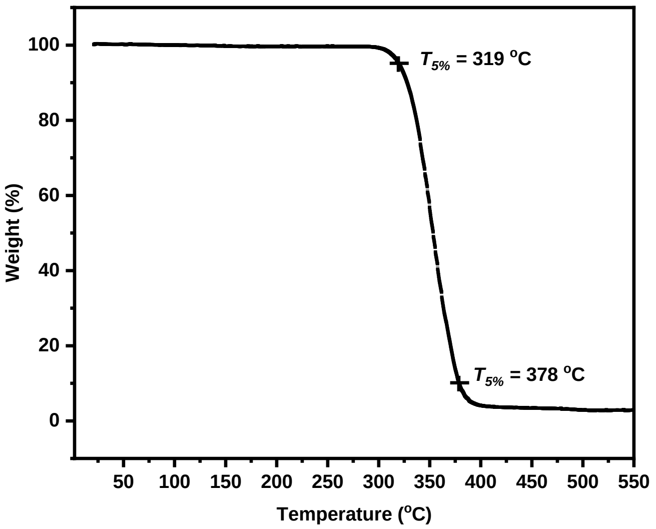

Supplementary Figure 55. TGA plot of purified poly (PC/TPA)-3h

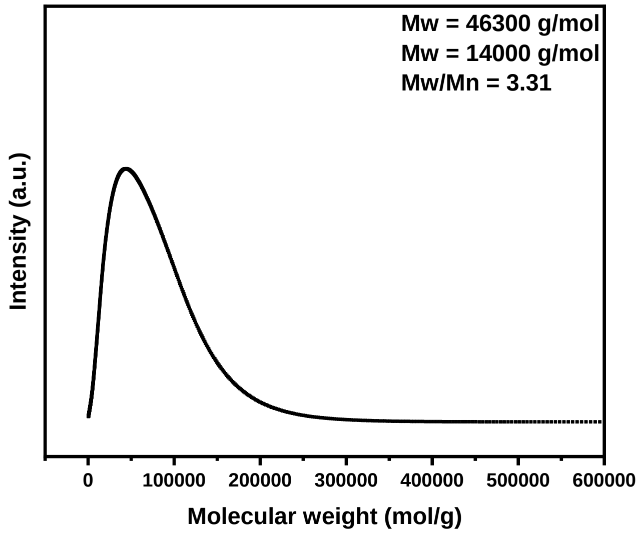

Supplementary Figure 56. GPC trace of purified poly (PC/TPA)-3h

Supplementary Table 11. Molecular-weight distributions and thermal properties data for synthesized poly (PC/TPA) [a]

Entry	Substrate	Catalyst	Yield[b] [%]	Mw[c] [g·mol^{-1}]	Mn[c] [g·mol^{-1}]	Đ[c]	$T_{5\%}$ [d] [°C]	$T_{90\%}$[d] [°C]	T_g[e] [°C]
1	Poly (PC/TPA)	$Zn(OAC)_2$	72.1	31700	14100	2.24	329	381	81
2	Poly (PC/TPA)	Sb_2O_3	-	-	-	-	-	-	-

[a] 3.35 mmol diol, 3.35 mmol DMTA, 1 mol % catalyst, 190 °C N_2/1h, 230 °C vacuum 1 mPa/3h; [b] Yield (%) = weight of collected product /weight of theoretical product × 100%; [c] Molecular weight distribution was determined by GPC; [d] $T_{5\%}$ and $T_{90\%}$ were determined by TGA characterization; [e] T_g was determined by DSC characterization.

3.2 Synthesis, analysis and characterization of poly (PC/FDCA)

Synthesis of poly (PC/FDCA): The two-step melt polymerizations (esterification and polycondensation) were performed according to General procedure C using equal molar ratio of PC and cellulose-derived DMFD over Titanium (IV) butoxide catalyst. Briefly, a 100 mL three-neck flask, equipped with a magnetic stirrer and reflux condenser, was charged with 0.53 g (3.35 mmol) of PC diol, 0.616 g (3.35 mmol) of DMFD and 1 mol % Titanium (IV) butoxide (TBT) catalyst. The esterification reaction was performed at 190 °C for 1 h under nitrogen flow. Then, the reaction temperature was increased to 230 °C, the pressure was slightly reduced to 1 mPa using an oil pulp for 1 h and 3 h. After that, the reaction mixture was cooled down to room temperature and the pressure was returned to atmospheric pressure by introducing of nitrogen. The copolymerization of PC with DMFD for the synthesis of poly (PC/FDCA) was shown in Supplementary Figure 57. Finally, 0.86 g and 0.82 g poly (PC/FDCA) was obtained as a yellow solid in 92.3 % yield (at vacuum for 3h) and 88 % (at vacuum for 1 h), respectively. The synthesized poly (PC/FDCA) was structurally characterized by ^{1}H NMR (Supplementary Figure 58), ^{13}C NMR (Supplementary Figure 59), 2D HSQC (Supplementary Figure 60), FTIR (Supplementary Figure 68) and GPC (Supplementary Figure 63). Thermal properties of the synthesized poly (PC/FDCA) were characterized by TGA (Supplementary Figure 62) and DSC (Supplementary Figure 61). Then poly (PC/FDCA)-3h was dissolved in chloroform (5 mL) and precipitated with methanol (50 mL) to obtain purified poly (PC/FDCA) solid (0.49 g) in a yield of 50.8 %. The purified poly (PC/FDCA)-3h was characterized by GPC (Supplementary Figure 66), TGA (Supplementary Figure 65) and DSC (Supplementary Figure 64).

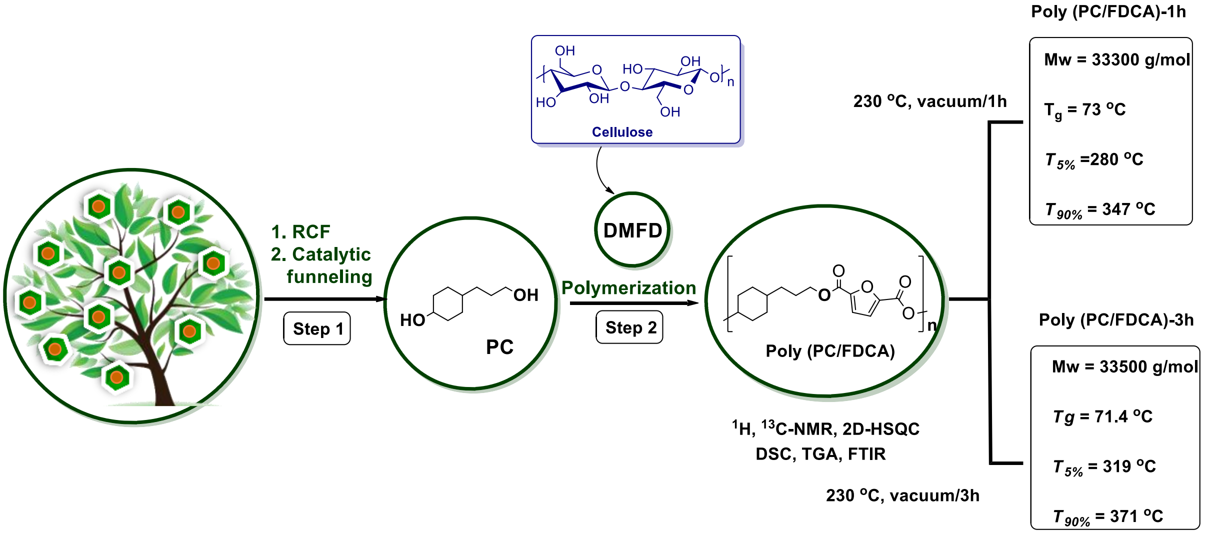

Supplementary Figure 57. The copolymerization of PC with DMFD for the synthesis of poly (PC/FDCA). Reaction conditions: 0.53 g (3.35 mmol) PC diol, 0.616 g (3.35 mmol) DMFD, 1 mol % TBT, 190 °C/N_2 for 1 h, 230 °C under vacuum for 1 h or 3 h.

^{1}H NMR, ^{13}C NMR and 2D HSQC spectra of poly (PC/FDCA)

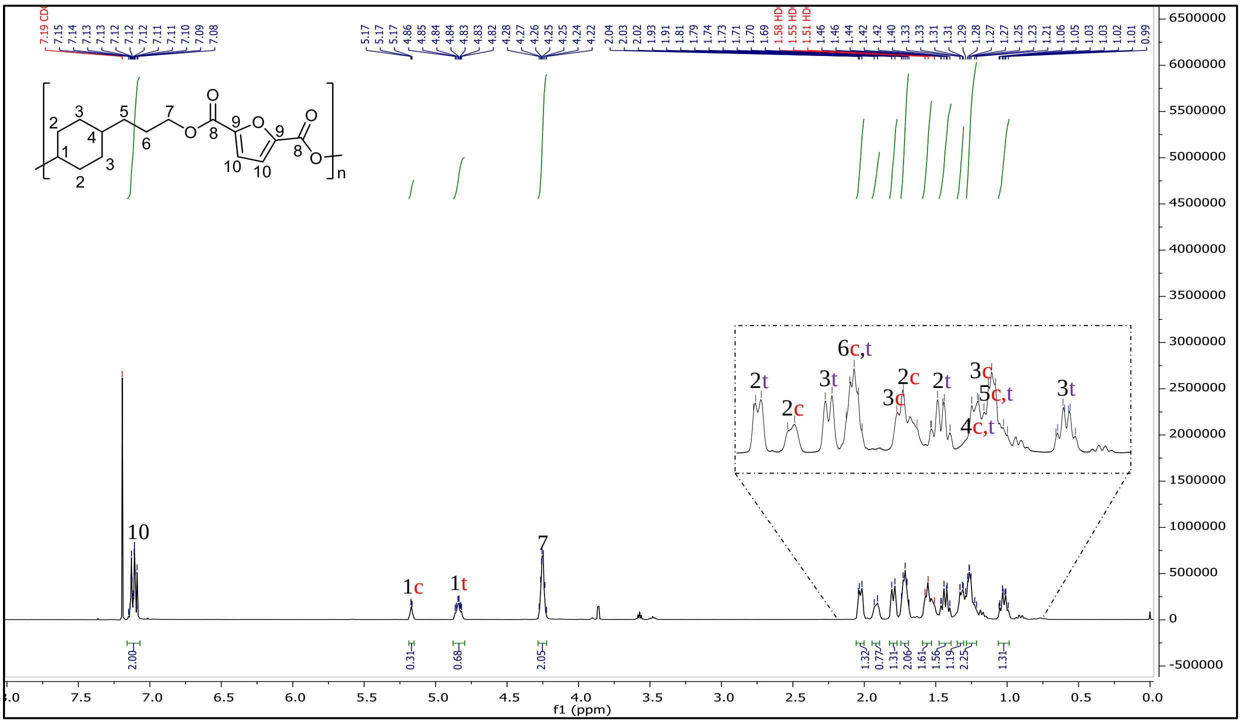

Supplementary Figure 58. ^{1}H NMR spectrum of poly (PC/FDCA).

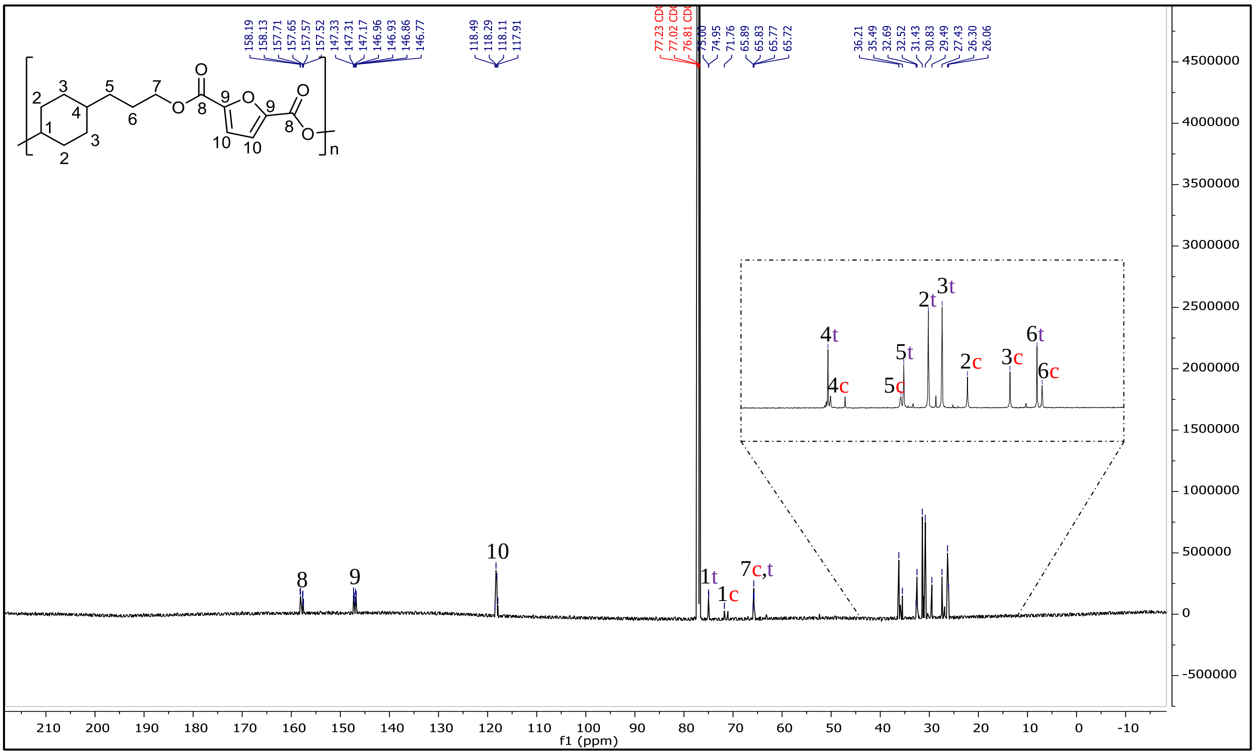

Supplementary Figure 59. ^{13}C NMR spectrum of poly (PC/FDCA).

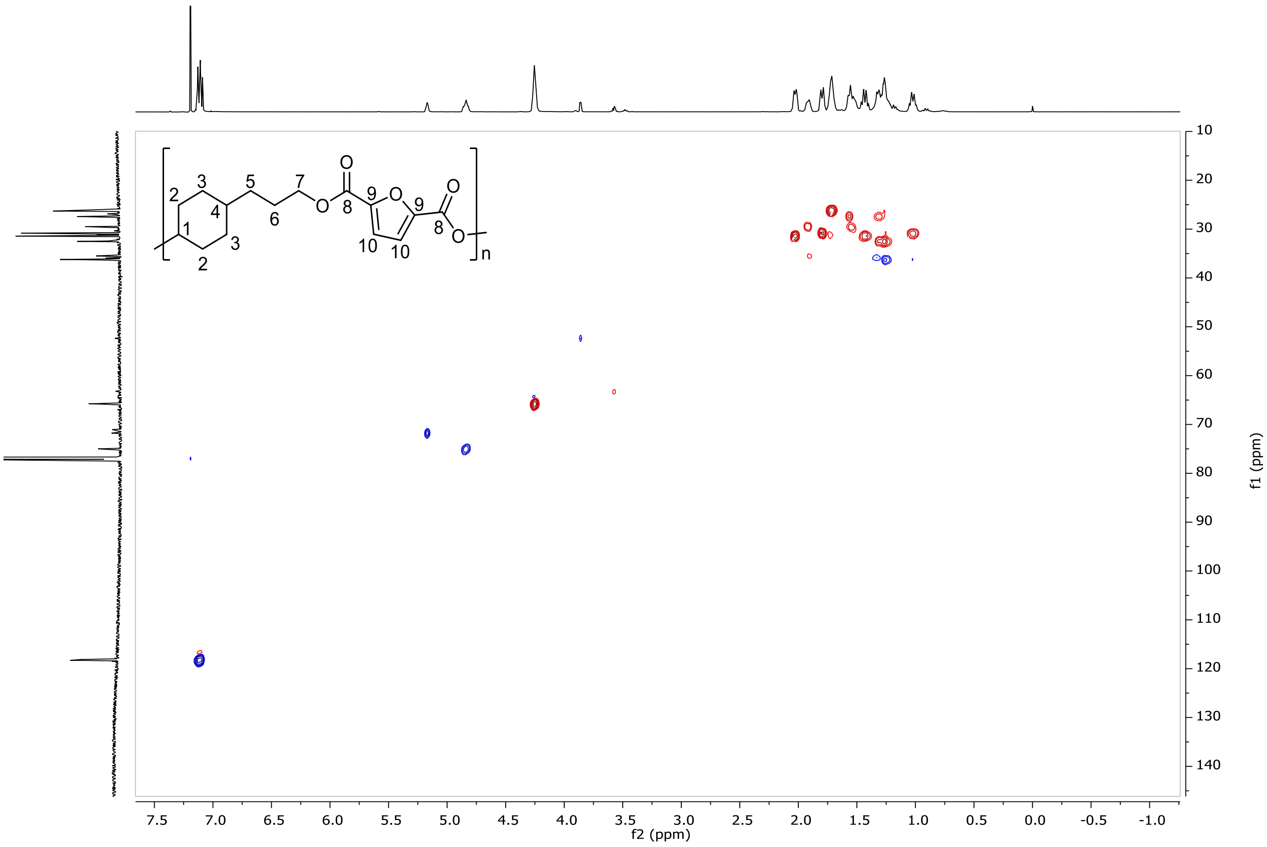

Supplementary Figure 60. 2D HSQC spectrum of poly (PC/FDCA).

3.2.1 DSC, TGA and GPC of poly (PC/FDCA)

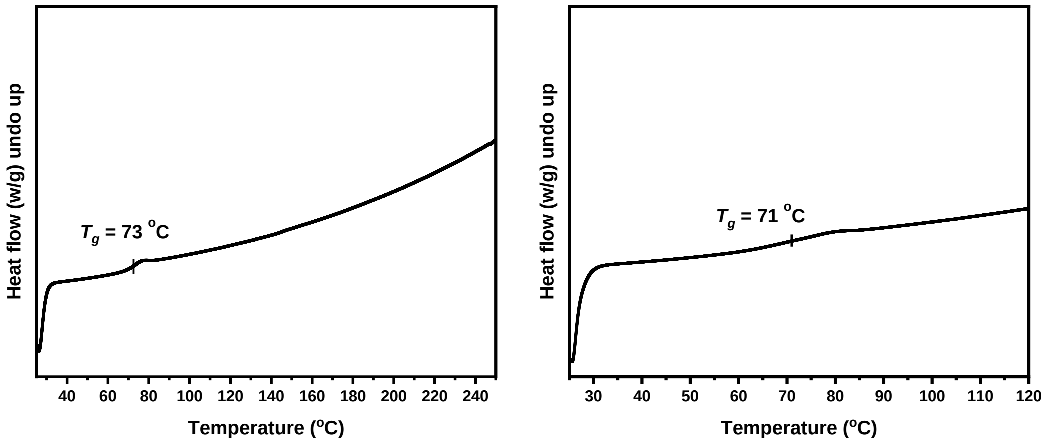

Supplementary Figure 61. The DSC thermograms of poly (PC/FDCA) at 230 °C under vacuum for 1h (left) and for 3h (right).

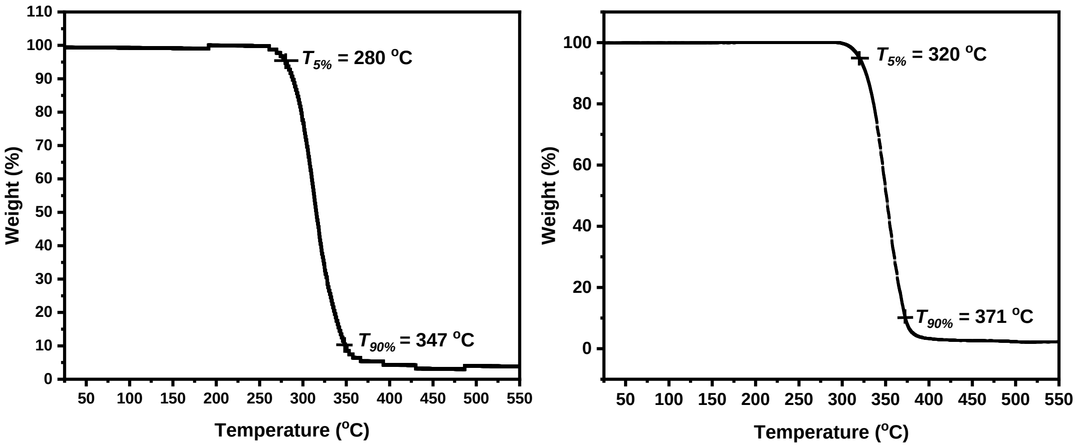

Supplementary Figure 62. The TGA plots of poly (PC/FDCA) at 230 °C under vacuum for 1h (left) and for 3h (right).

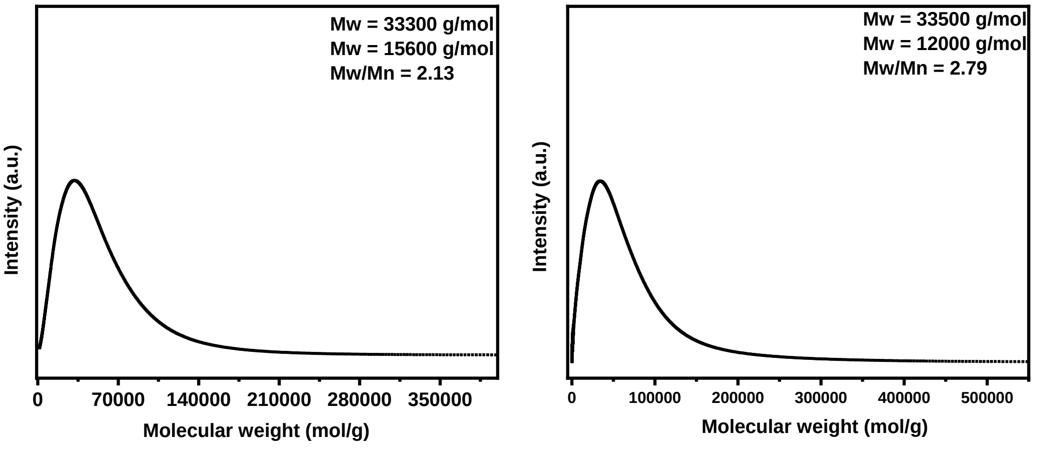

Supplementary Figure 63. The GPC traces of poly (PC/FDCA) at 230 °C under vacuum for 1h (left) and for 3h (right).

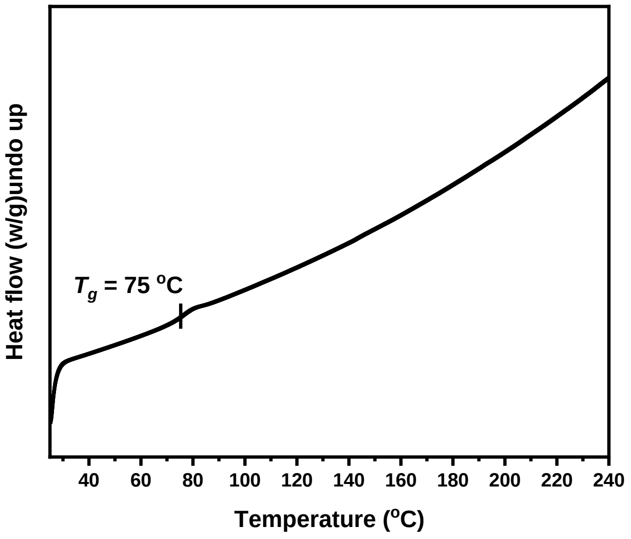

Supplementary Figure 64. DSC thermogram of purified poly (PC/FDCA)-3h

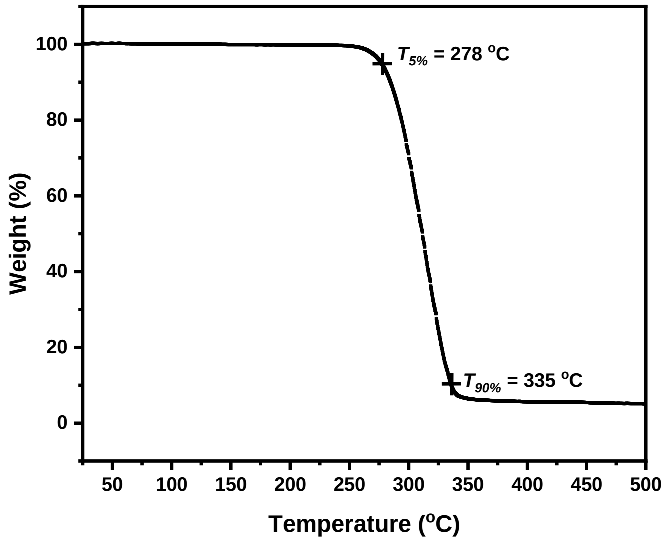

Supplementary Figure 65. TGA plot of purified poly (PC/FDCA)-3h

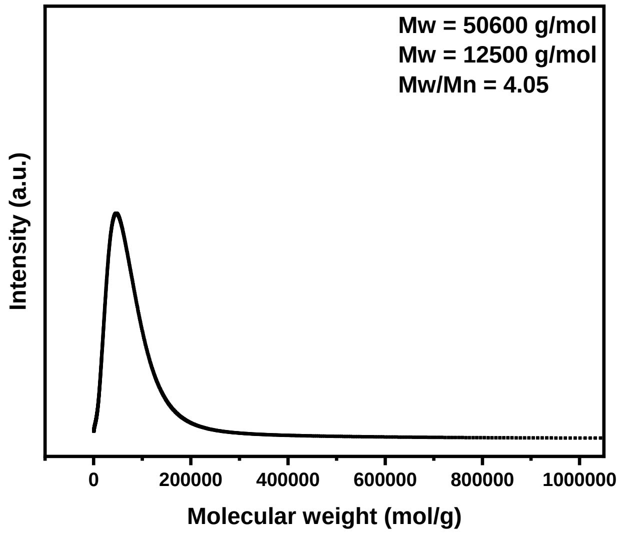

Supplementary Figure 66. GPC trace of purified poly (PC/FDCA)-3h

Detailed structural analysis of poly (PC/FDCA)

To understand poly (PC/FDCA) dyad structure, similar experiments and characterizations were carried out as those described in the case of poly (PC/FDCA). According to literature data, the C8 in dicyclohexyl and dipropyl furandicarboxylate display a chemical shift at 157.6 ppm[21] and 158.2 ppm[22]. Therefore, we proposed that the high field signals at 157.55 ppm (C8 cis) and 157.72 ppm (C8 trans) belong to H-H type esters, low field signal at 158.2 ppm (C8 cis and C8 trans overlapped) to T-T type and the signals in-between (158.15 ppm, 157.7 ppm, and 157.58 ppm) to mixed (H-T, T-H) type connectivity. Based on the integrated value of dyads obtained from the quantitative ^{13}C NMR spectrum (Supplementary Figure 67), the signals of C8 were assigned accordingly to T-T, T-H and H-H type of dyads, which displayed a random distribution: [H-H] = [T-T] = 0.25 and [H-T] = 0.50.

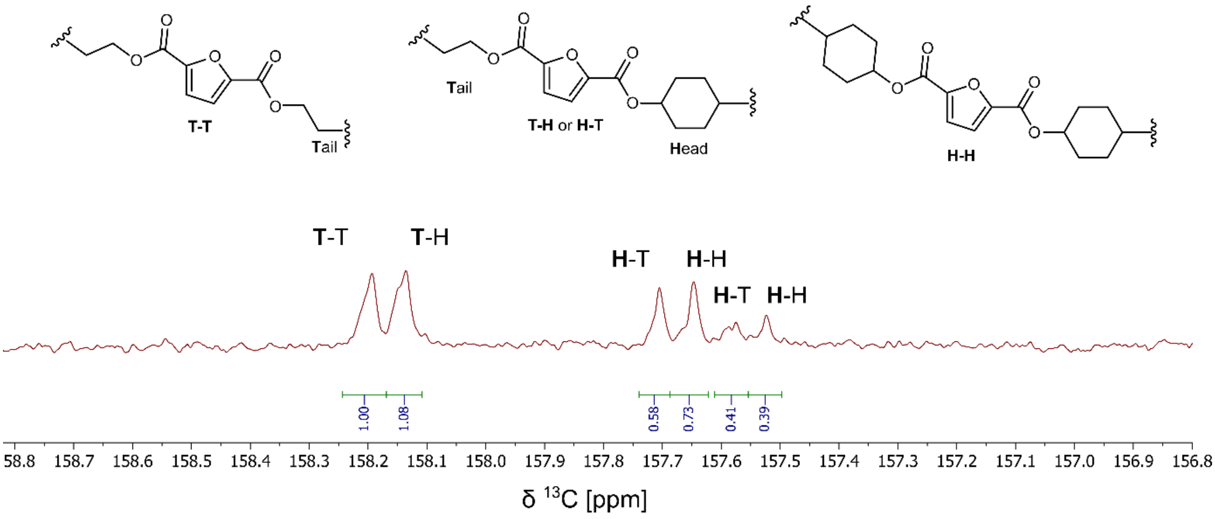

Supplementary Figure 67. The identification spectrum of carbonyl carbon signals (C8) of poly (PC/FDCA).

$$\Sigma_{T-T} = 1.00 \quad \text{Eq31}$$

$$\Sigma_{H-T} = 1.08 + 0.58 + 0.41 = 2.07 \quad \text{Eq32}$$

$$\Sigma_{H-H} = 0.73 + 0.39 = 1.12 \quad \text{Eq33}$$

$$\Sigma_{all} = 1.00 + 2.07 + 1.00 = 4.07 \quad \text{Eq34}$$

$$\chi_{T-T} = \frac{1.00}{4.07} \times 100\% = 24\% \quad \text{Eq35}$$

$$\chi_{H-H} = \frac{1.12}{4.07} \times 100\% = 26\% \quad \text{Eq36}$$

$$\chi_{H-T} = \frac{2.07}{4.07} \times 100\% = 50\% \quad \text{Eq37}$$

3.3 The FTIR spectra of poly (PC/TPA) and poly (PC/FDCA)

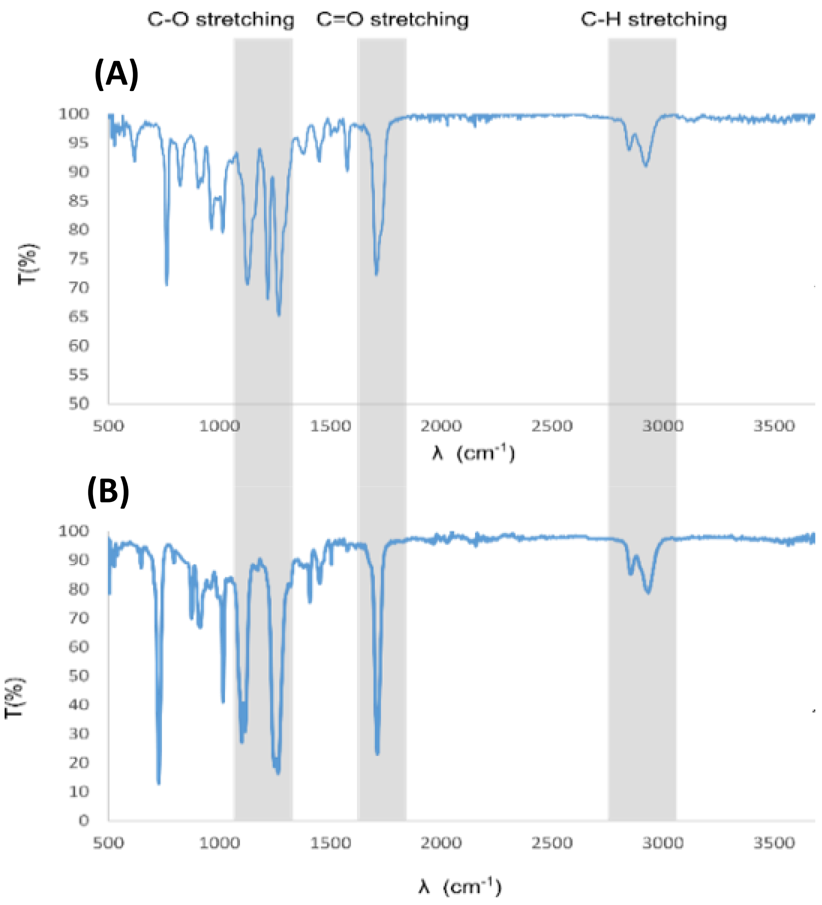

Supplementary Figure 68. FTIR spectra of A) poly (PC/TPA) and B) poly (PC/FDCA)

In all cases, nearly no OH absorption band at around 3500 cm^{-1} is observable, which indicated successful copolymerization of PC with DMTA and DMFD by the formation of ester bonds. According to the literature reported FTIR data for poly (EG/TPA)[23] and poly (EG/FDCA)[24], a clear signals assignments can be made. Poly (PC/TPA): The absorption peak at 1711 cm^{-1} represents C=O stretching (ester). The peaks at 1265, 1247, and 1117, 1101 cm^{-1} represent C-O stretching (ester) and -C-O stretching (ester), respectively. Absorption peaks at 2934 and 2855 cm^{-1} are due to the asymmetric C-H stretching. Poly (PC/TPA) IR (ATR): 2934, 2855, 1711, 1265, 1247, 1117, 1101, 727 cm^{-1}. Poly (PC/FDCA): The absorption peak at 1711 cm^{-1}, represent C=O stretching (ester). The peaks at 1271 and 1219 cm^{-1} represent C-O stretching (ester), where the peak at 1128 cm^{-1} represents -C-O stretching (ester). The peaks at 1016 cm^{-1} represents C-O stretching (ring), and absorption at 2932 and 2855 cm^{-1} is due to the asymmetric C-H stretching. C-H ring binding vibrations lead to absorption peak at 966 cm^{-1}. Poly (PC/FDCA) IR (ATR): 2932, 2855, 1711, 1271, 1219, 1128, 1016, 966, 764 cm^{-1}.

3.4 Copolymerization with TPA and FDCA using crude aliphatic alcohols mixture from defunctionalization of the model 1G/1S mixture

Step a: The demethoxylation and hydrogenation of a mixture of model 1G/1S was carried out according to General procedure C in a 100 mL high pressure Parr autoclave equipped with an overhead stirrer. Typically, the autoclave was charged with 2 g Raney Ni catalyst, 0.546 g (3 mmol) of 1G, 0.636 g (3 mmol) of 1S, 20 mL isopropanol. The reactor was sealed and pressurized with H_2 (20 bar). The reactor was heated and stirred at 130 °C for 4 h. After reaction, the reactor was cooled to room temperature and the solvent was removed to give aliphatic alcohol mixtures (0.945 g) rich in PC (82 %) and **1** (13 %) diol (GC selectivity: 95 % for PC/**1**). The obtained mixture was divided into average two portions. **Step b**: To one portion (0.472 g) was added 0.578 g (2.98 mmol) of DMTA (the specified DMTA amount was calculated based on crude mixture containing 100 % of PC diol) and 1 mol % $Zn(OAc)_2$ in a 100 mL three-neck round bottom flask. Then the crude mixture was heated to 190 °C for 2 h under nitrogen flow. The pressure was slightly reduced to 1 mPa using an oil pulp. The reaction was considered to be complete under vacuum for 1 h. The pathway for the synthesis of poly (PC/**1**/TPA) and poly (PC/**1**/FDCA) was shown in Supplementary Figure 69. Finally, 0.53 g (61.8 % yield) of white and transparent solid poly (PC/**1**/TPA) was obtained. The obtained poly (PC/**1**/TPA) was characterized by GPC (Supplementary Figure 74), TGA (Supplementary Figure 73), DSC (Supplementary Figure 72) and ^{1}H NMR (Supplementary Figure 70), respectively. To the other portion (0.472 g) was added 0.548 g (2.98 mmol) of DMFD and 1 mol % TBT catalyst in a 100 mL three-neck round bottom flask. Then the crude mixture was heated to 190 °C for 2 h under nitrogen protection. The pressure was slightly reduced to 1 mPa using an oil pulp. The reaction was considered to be complete under vacuum for 3 h. Finally, 0.68 g (79.1 % yield) of brown-yellow solid poly (PC/**1**/FDCA) was obtained. The obtained poly (PC/**1**/FDCA) was characterized by GPC (Supplementary Figure 74), TGA (Supplementary Figure 73), DSC (Supplementary Figure 72), and ^{1}H NMR (Supplementary Figure 71), respectively.

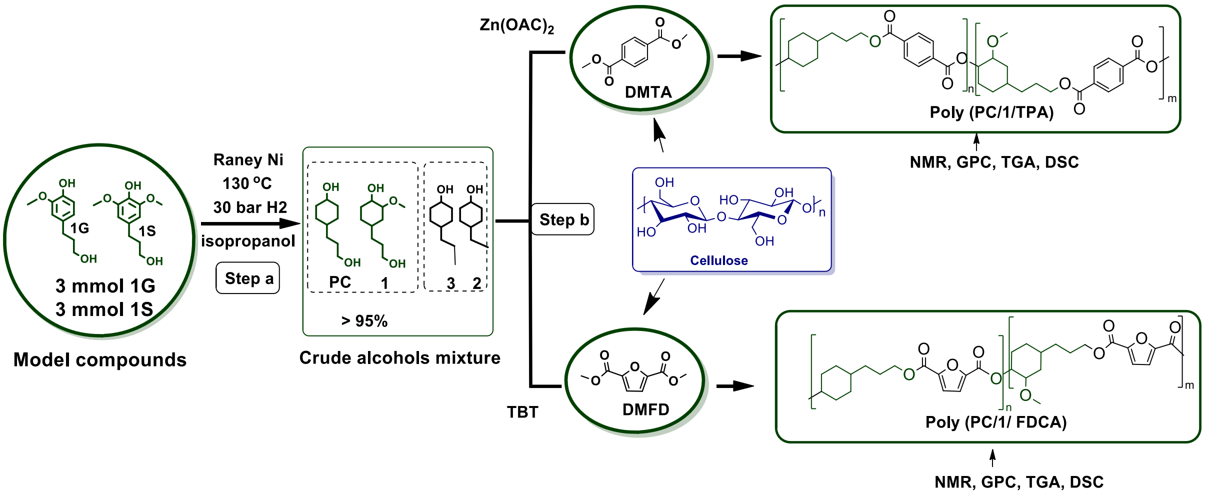

Supplementary Figure 69. The catalytic synthesis procedure of biomass-derived poly (PC/**1**/TPA) and poly (PC/**1**/FDCA) from crude alcohols mixture starting from defunctionalization of 1G and 1S mixture.

^{1}H NMR, ^{13}C NMR and 2D HSQC spectra of poly (PC/1/TPA) and poly (PC/FDCA)

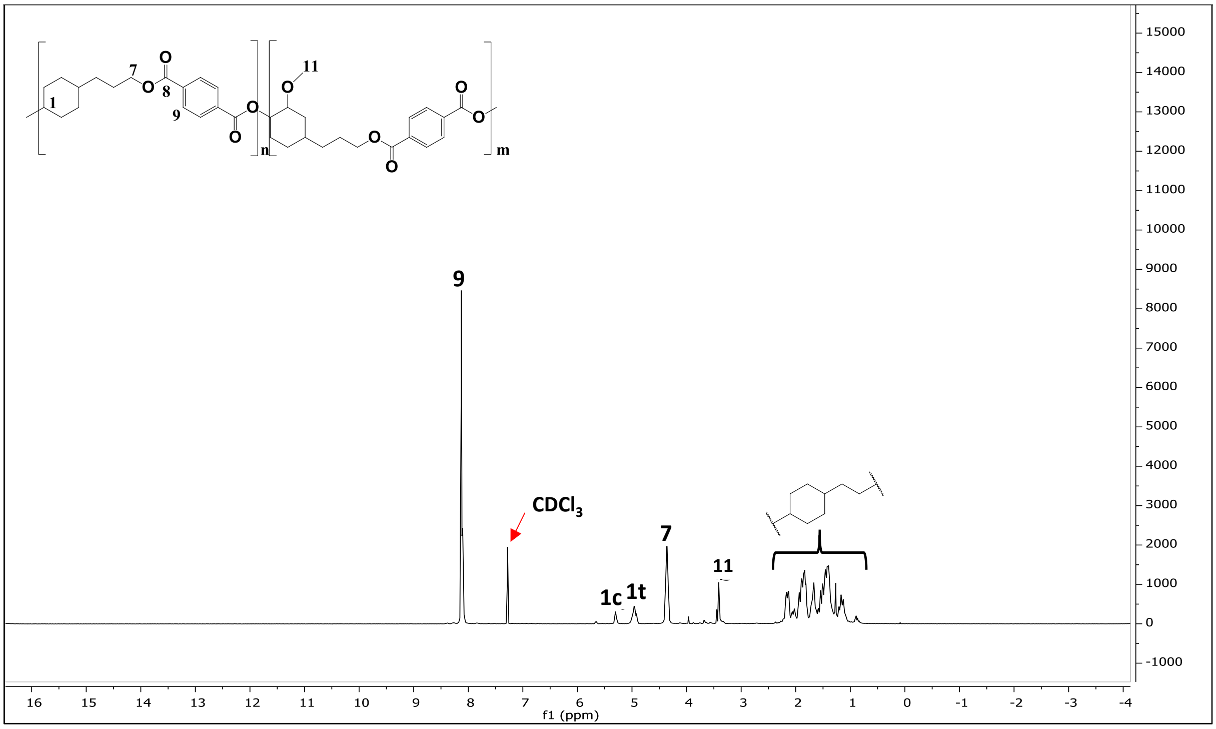

Supplementary Figure 70. ^{1}H NMR spectrum of poly (PC/**1**/TPA).

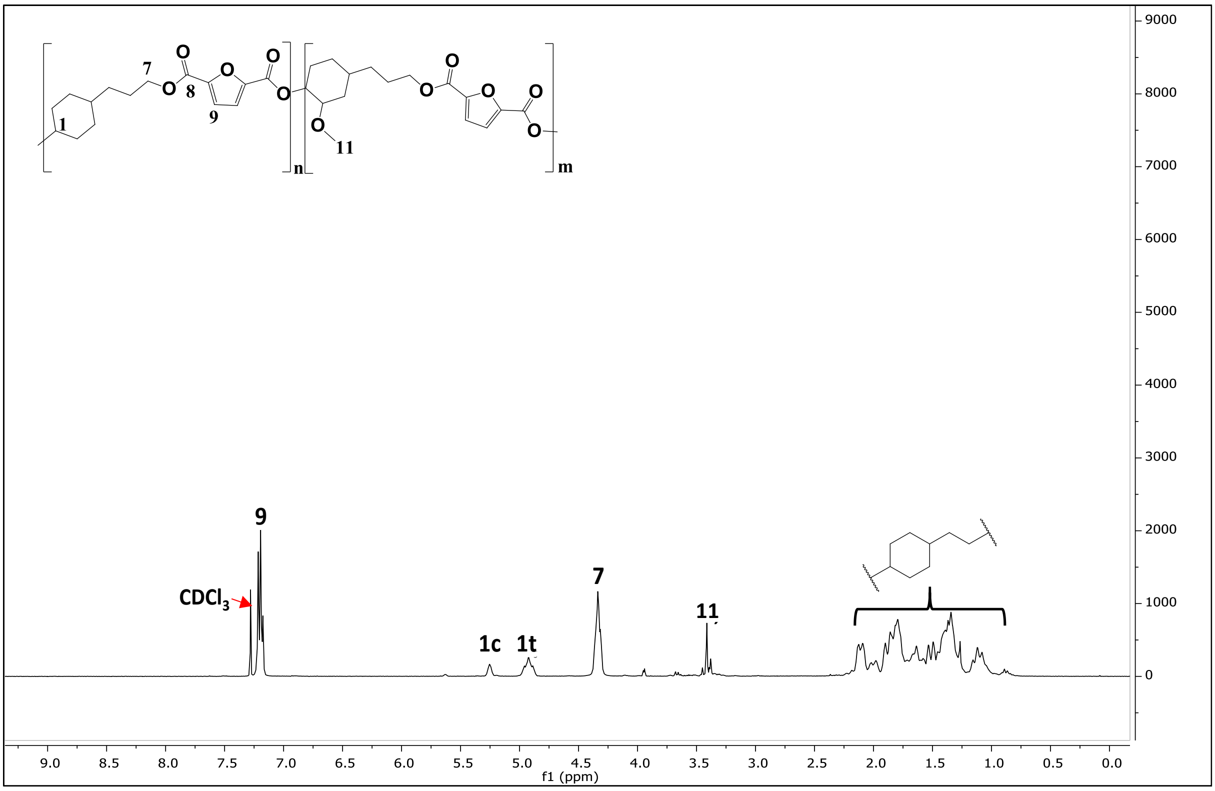

Supplementary Figure 71. ^{1}H NMR spectrum of poly (PC/**1**/FDCA).

3.4.1 DSC, TGA and GPC of poly (PC/1/TPA) and poly (PC/1/FDCA)

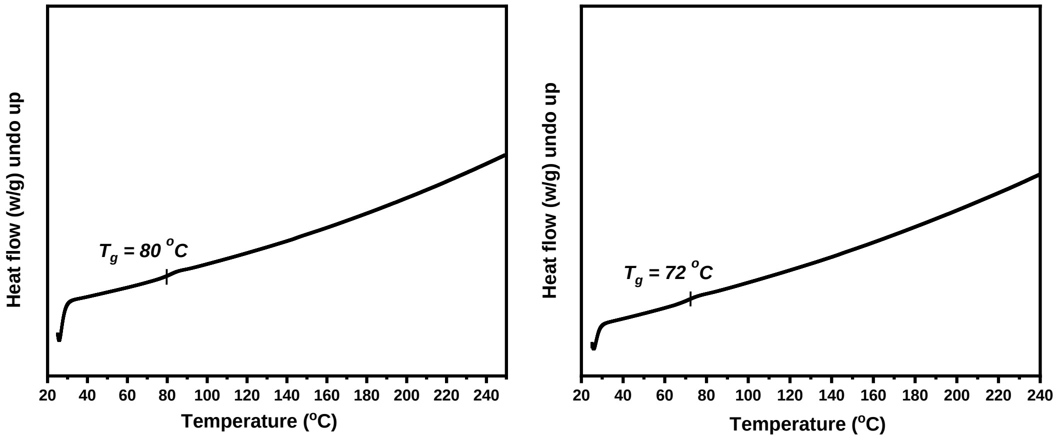

Supplementary Figure 72. DSC thermograms for poly (PC/**1**/TPA) (left) and poly (PC/**1**/FDCA) (right).

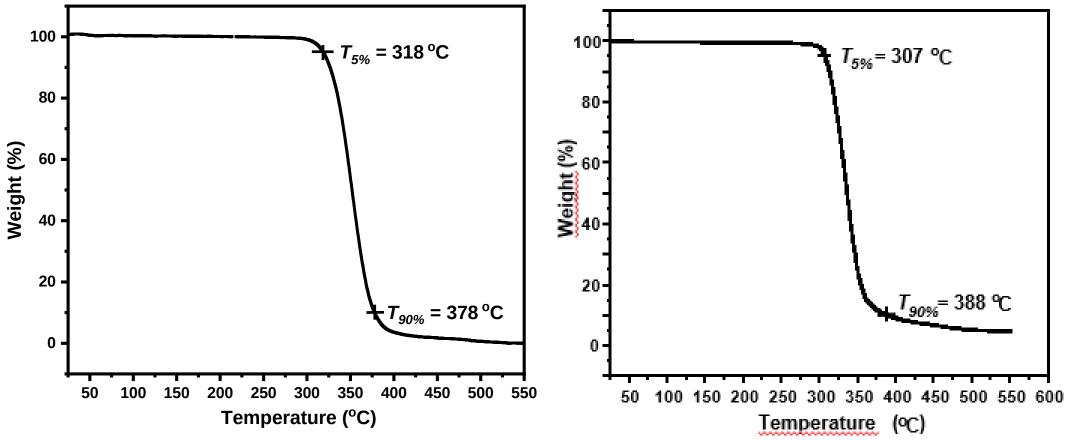

Supplementary Figure 73. TGA plots for poly (PC/**1**/TPA) and poly (PC/**1**/ FDCA).

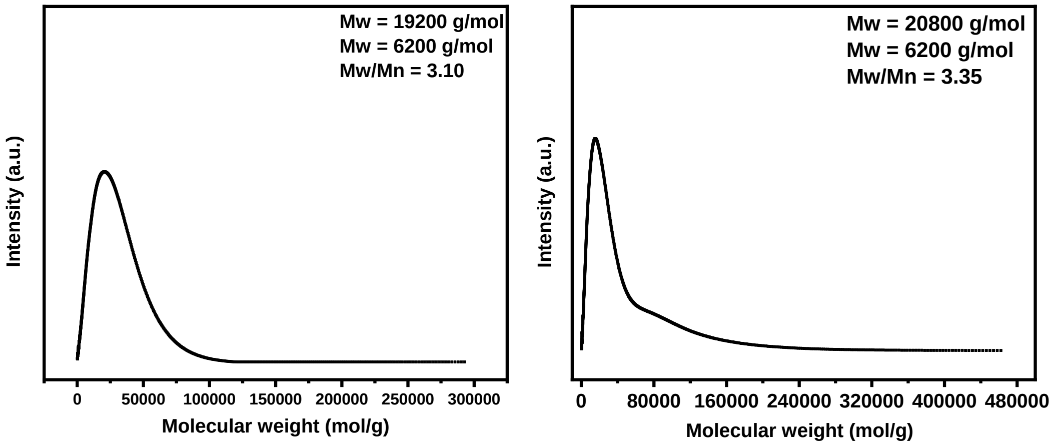

Supplementary Figure 74. GPC traces for poly (PC/**1**/TPA) and poly (PC/**1**/ FDCA).

3.5 A comprehensive biorefinery strategy for the production of gasoline, PET analogues and jet fuels from beech wood (10 g)

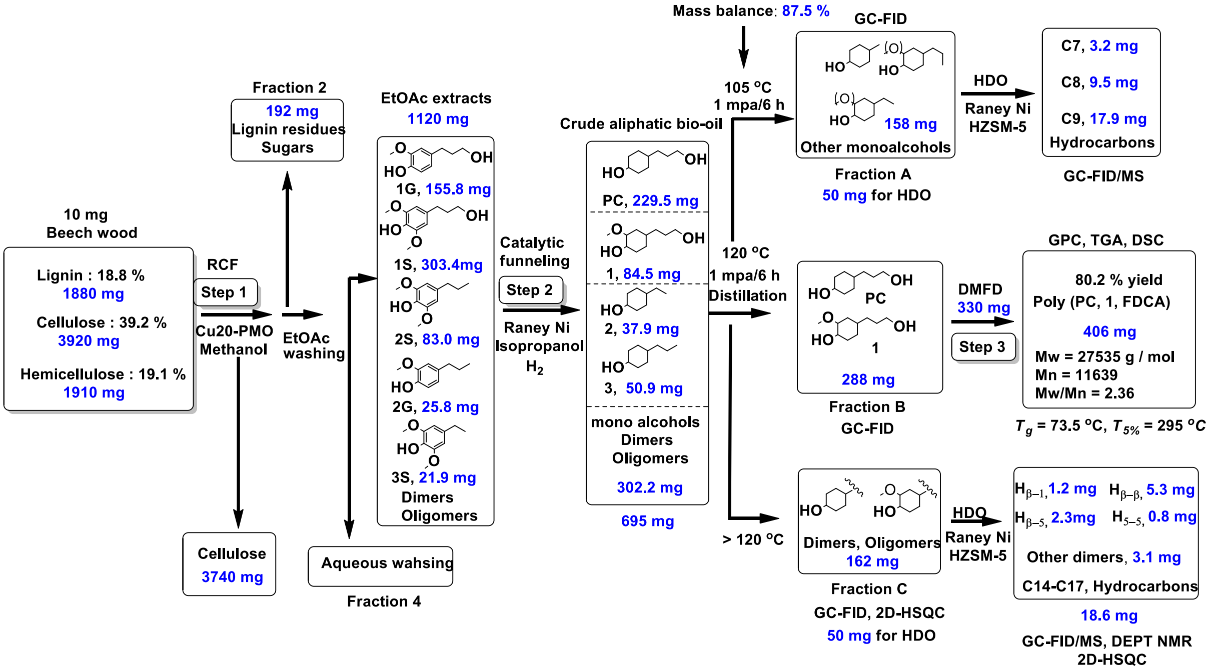

Supplementary Figure 75. A comprehensive and proposed catalytic protocol for complete utilization of beech wood. Step 1: RCF of beech wood gave crude aromatic bio-oil over Cu20-PMO catalyst; Step 2: Catalytic funneling of EtOAc extracts gave crude aliphatic bio-oil. The crude aliphatic bio-oil was purified by distillation under 1mpa at (100 – 120 °C) to deliver three Fractions A , B and C. HDO of Fraction A and Fraction C gave C7-C9 and C14-C17 cyclic alkanes, respectively.The hydrocarbons were quantified using the response of the flame-ionization detector (FID) and the response factors was estimated by Effective Carbon Number method (ECN); Step 3: Copolymerization of Fraction B with cellulose-derived DMFD to yield PET analogue poly (PC/**1**/FDCA).

Step 1: A large scale reductive catalytic fractionation (RCF) setup using beech wood was carried out according to General procedure A over our previously developed Cu20-PMO catalyst under the following reaction conditions: 10 g beech wood, 2 g catalyst, 180 °C, 120 mL methanol, 40 bar H_2, 18 h. RCF of beech wood gave crude aromatic bio-oil (1531 mg), which consisted of monomers (1G, 164.6 mg, 1S, 310.3 mg, 2S, 88.1 mg, 2G, 28.9 mg and 3S, 26.4 mg), dimers, oligomers, lignin residues, and sugars, as discussed above (Supplementary Note 2.5). To the crude aromatic bio-oil (1531 mg) 100 mL of EtOAc was added and the suspension was stirred overnight. Lignin residues and small amounts of sugars were precipitated as (192 mg) brownish colored solid and separated by centrifugation, decantation and washed with EtOAc (20 mL). The EtOAc soluble fraction was washed immediately with small amount of saturated $NaHCO_3$ (1 × 10 mL) and brine (2 × 20 mL) to remove small possible organic acids and carbohydrate residues, that may be detrimental to the activity of the Raney Ni catalyst, to deliver EtOAc extracts (1120 mg) (1G, 155.8 mg, 1S, 303.4 mg, 2S, 83.0 mg, 2G, 25.8 mg and 3S, 21.9 mg). Then the EtOAc extracts were transferred in a round bottom flask and the solvent was removed in vacuo. The crude product was dried in a desiccator in vacuo overnight and was further used as specified below. **Step 2:** The catalytic funneling of EtOAc extracts was carried out according to General procedure B in 100 mL high pressure Parr autoclave with an overhead stirrer. Typically, the autoclave

was charged with 2 g Raney Ni catalyst, 1120 mg of EtOAc extracts, 20 mL isopropanol. The reactor was sealed and pressurized with H_2 (30 bar). The reactor was heated and stirred at 150 °C for 10 h. After reaction, the reactor was cooled to room temperature and the solvent was removed in vacuo to deliver crude aliphatic bio-oil (PC, 229.5 mg, **1**, 84.5 mg, **2**, 37.9 mg, **3**, 50.9 mg). Crude aliphatic bio-oil was subjected to distillation at temperature range between 100-120 °C using a Kugelrohr apparatus under vacuum 1 mPa, to provide 158 mg of Fraction A, 288 mg of Fraction B and 162 mg of Fraction C (Supplementary Figure 75). Fraction C was then characterized by ^{1}H NMR (Supplementary Figure 83), GC-FID (Supplementary Figure 82) and 2D HSQC (Supplementary Figure 85), which confirmed the presence of dimers comprising cyclohexanol structural moiety with methoxy group or free in meta-position. **Step 3:** To the Fraction B (PC, 215.6 mg and **1**, 72.4 mg. Amount was determined based on GC selectivity (Supplementary Figure 77) (78 % of PC and 22 % of **1**) was added 322 mg (1.75 mmol) of DMFD and 1 mol % TBT catalyst in a 100 mL three-neck round bottom flask. Then the crude mixture was heated to 190 °C for 1 h under nitrogen flow. The pressure was slightly reduced to 1 mPa using an oil pulp and the mixture was heated to 230 °C. The reaction was considered to be complete under vacuum for 3 h. Finally, 406 mg (81.5 % yield) of poly (PC/**1**/FDCA) was obtained. The obtained biomass-derived poly (PC/**1**/FDCA) were characterized by ^{1}H NMR (Supplementary Figure 78), GPC (Supplementary Figure 79), TGA (Supplementary Figure 80), DSC (Supplementary Figure 81) respectively. The Fraction A constitutes cyclohexanol derivatives, mainly 4-ethyl and propyl cyclohexanol (Supplementary Figure 76), which were selectively hydrodeoxygenated (200 mg wet Raney Ni, 100 mg H-ZSM-5, 20 mL cyclohexane, 220 °C, 30 bar H_2, 4 h), to give predominately 4-ethyl cyclohexane (C7) (9.5 mg) and 4-propyl cyclohexane (C8) (17.9 mg), as well as a small amount of 4-methyl cyclohexane (C6) (3.2 mg). These compounds are gasoline range alkanes. The Fraction C was subjected to hydrodeoxygenation (HDO), over Raney Ni and HZSM-5 co-catalyst (200 mg wet Raney Ni, 100 mg HZSM-5, 20 mL cyclohexane, 220 °C, 30 bar H_2, 6 h) to give predominately dicyclohexane derivatives (18.6 mg) ($H_{β-1}$, 1.2 mg, $H_{β-5}$, 2.3 mg, $H_{β-β}$, 5.3 mg, H_{5-5}, 0.8 mg and unrecogonised dimers, 3.1 mg. The amount of the respective hydrocarbons was determined by ECN method where the response factor was assumed as 1.0. The product mixture was also characterized by GC-FID (Supplementary Figure 82), ^{1}H NMR (Supplementary Figure 83), DEPT NMR (Supplementary Figure 84) and 2D HSQC (Supplementary Figure 86). These measurements revealed the disappearance of the hydroxyl- and methoxy- moieties (signals in the range of 3.0 – 4.0 ppm) to deliver predominately cyclic and bicyclic alkane dimers and small amounts of oligomers (~ 5 mg) (only -CH, manily -CH_2 and -CH_3 signals at 0.5 - 2.0 ppm were observed). The GC-MS results further confirmed these alkanes originated from β-1, β-5, β-β and 5-5 linked aromatic dimer units in native lignin, which were also observed after RCF of hardwood in literature[25, 26].

3.5.1 Fraction A: analysis, characterization, and hydrodeoxygenation

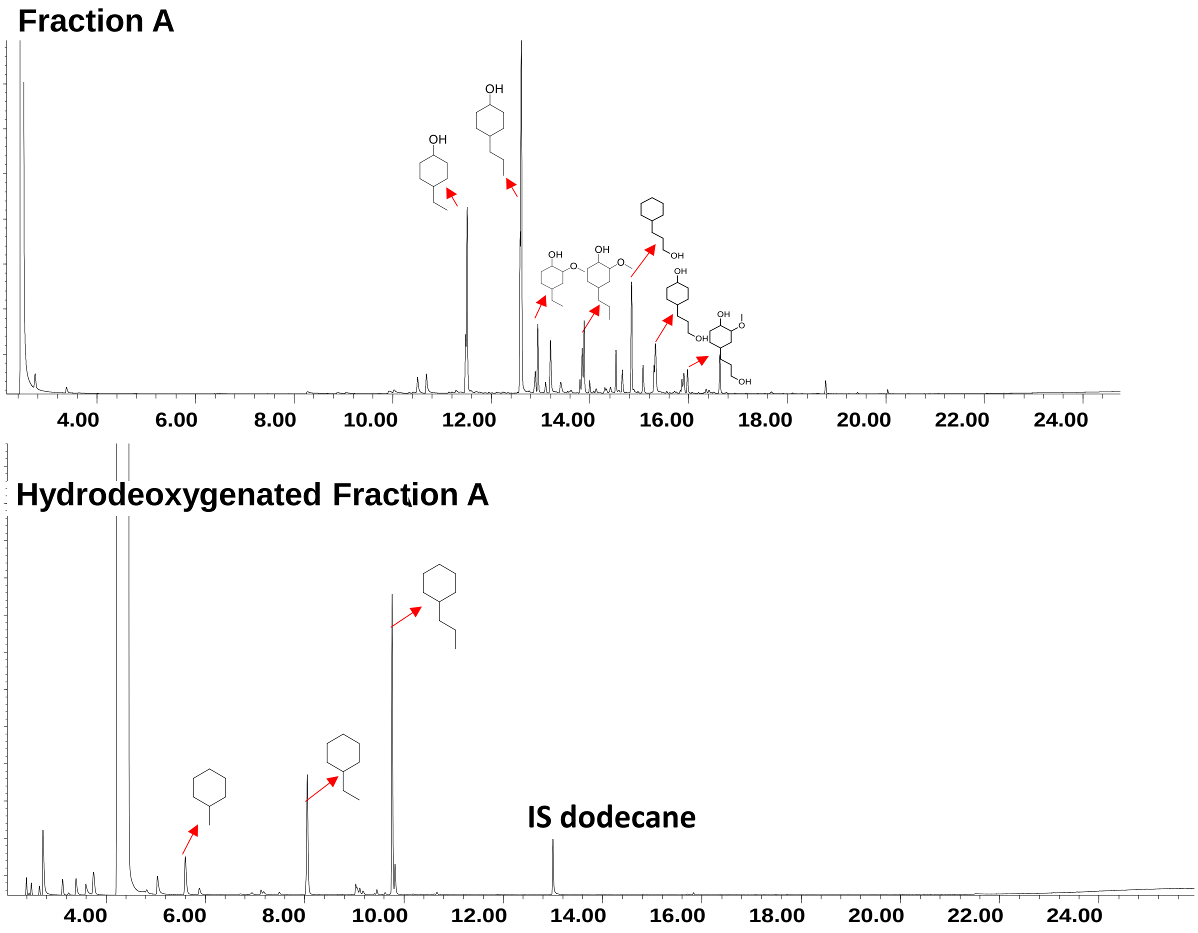

Supplementary Figure 76. GC-FID traces of Fraction A and hydrodeoxygenated Fraction A

3.5.2 Fraction B: analysis, characterization, and polymerization

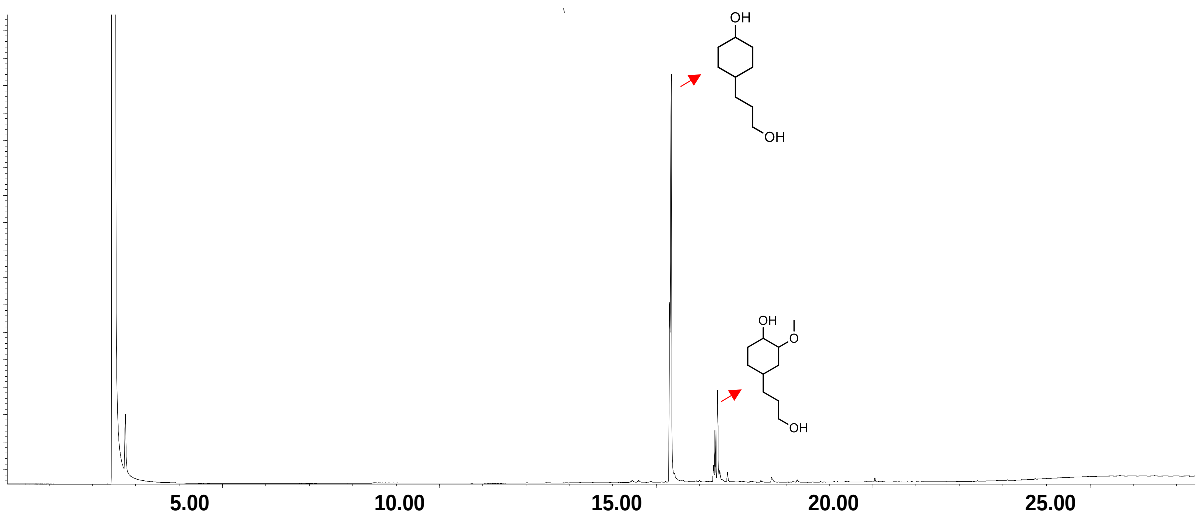

Supplementary Figure 77. GC-FID trace of Fraction B

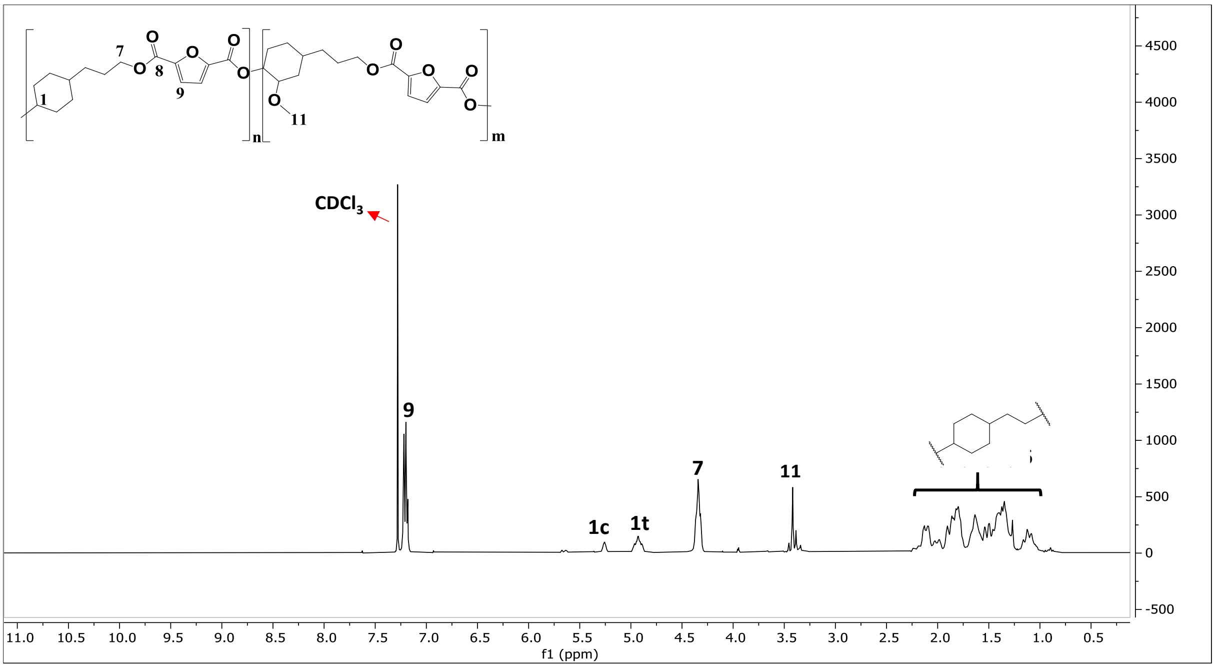

Supplementary Figure 78. ^{1}H NMR of biomass-derived poly (PC/**1**/FDCA)

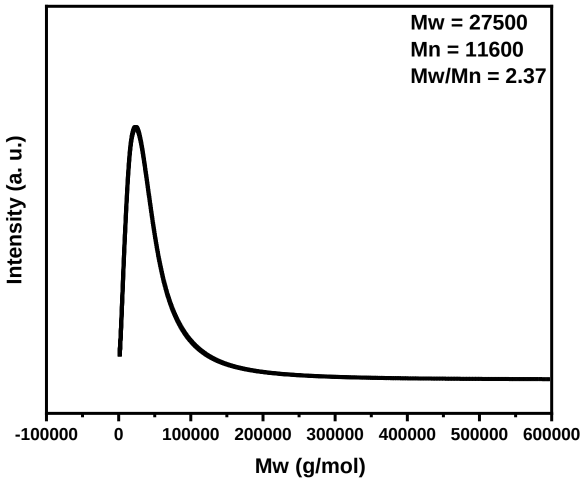

Supplementary Figure 79. GPC trace for synthesis of biomass-derived poly (PC/**1**/FDCA)

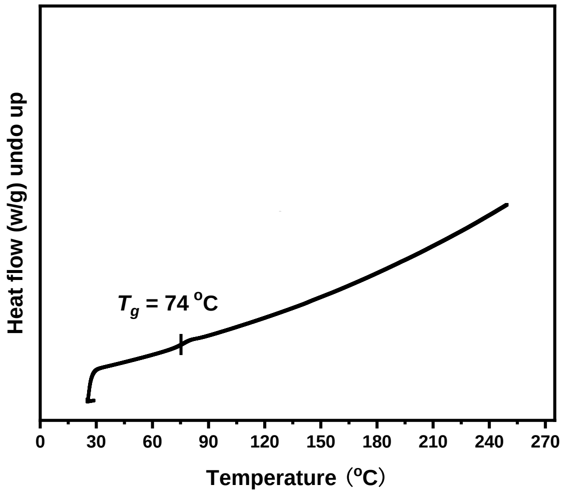

Supplementary Figure 80. DSC thermogram for synthesis of biomass-derived poly (PC/**1**/FDCA)

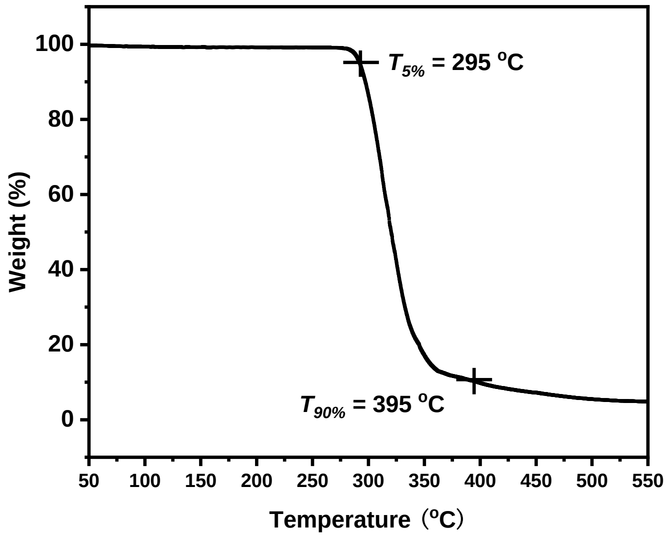

Supplementary Figure 81. TGA plot for synthesis of biomass derived poly (PC/**1**/FDCA)

3.5.3 Fraction C: analysis, characterization, and hydrodeoxygenation

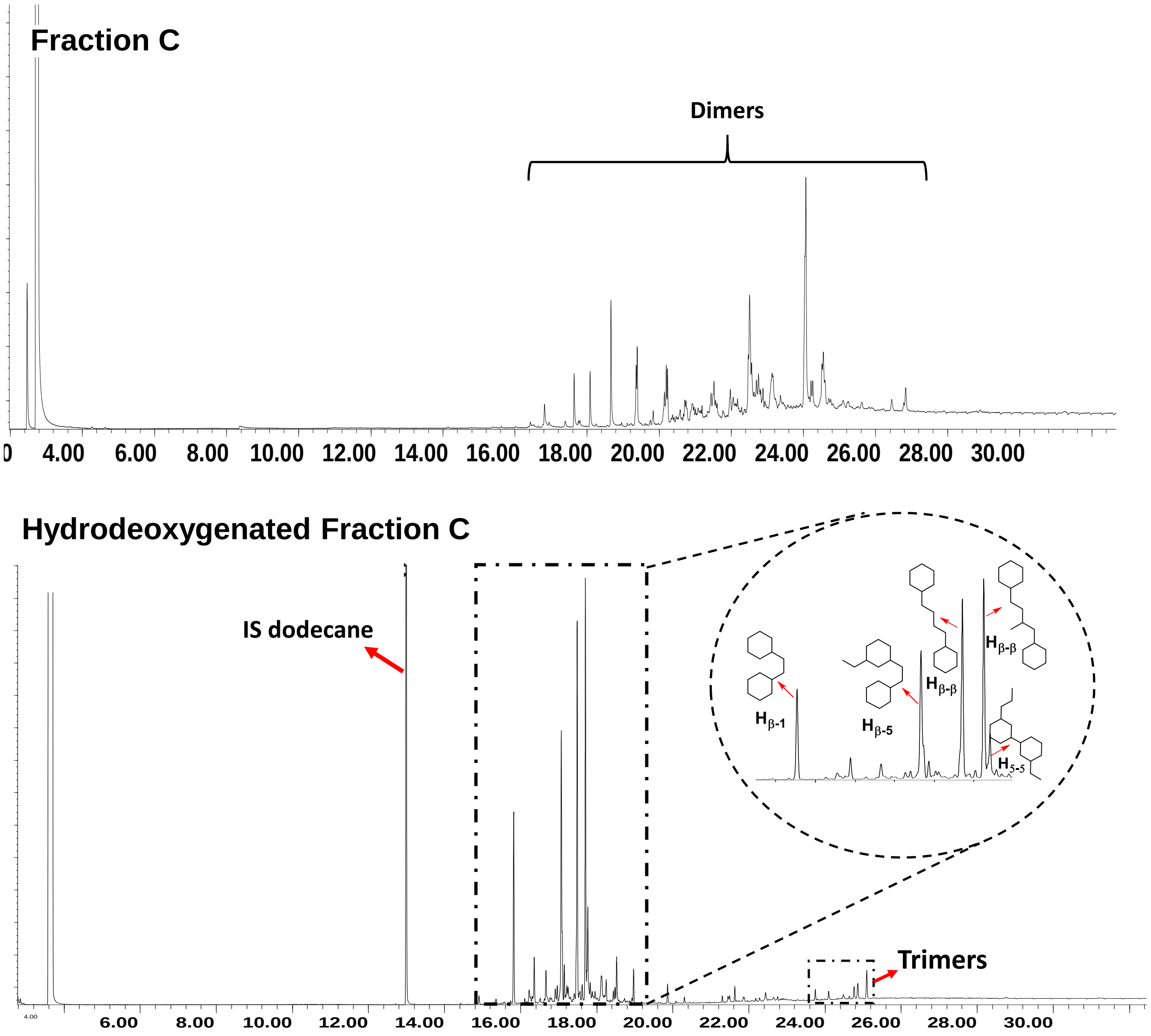

Supplementary Figure 82. GC-FID chromatograms of Fraction C and hydrodeoxygenated Fraction C

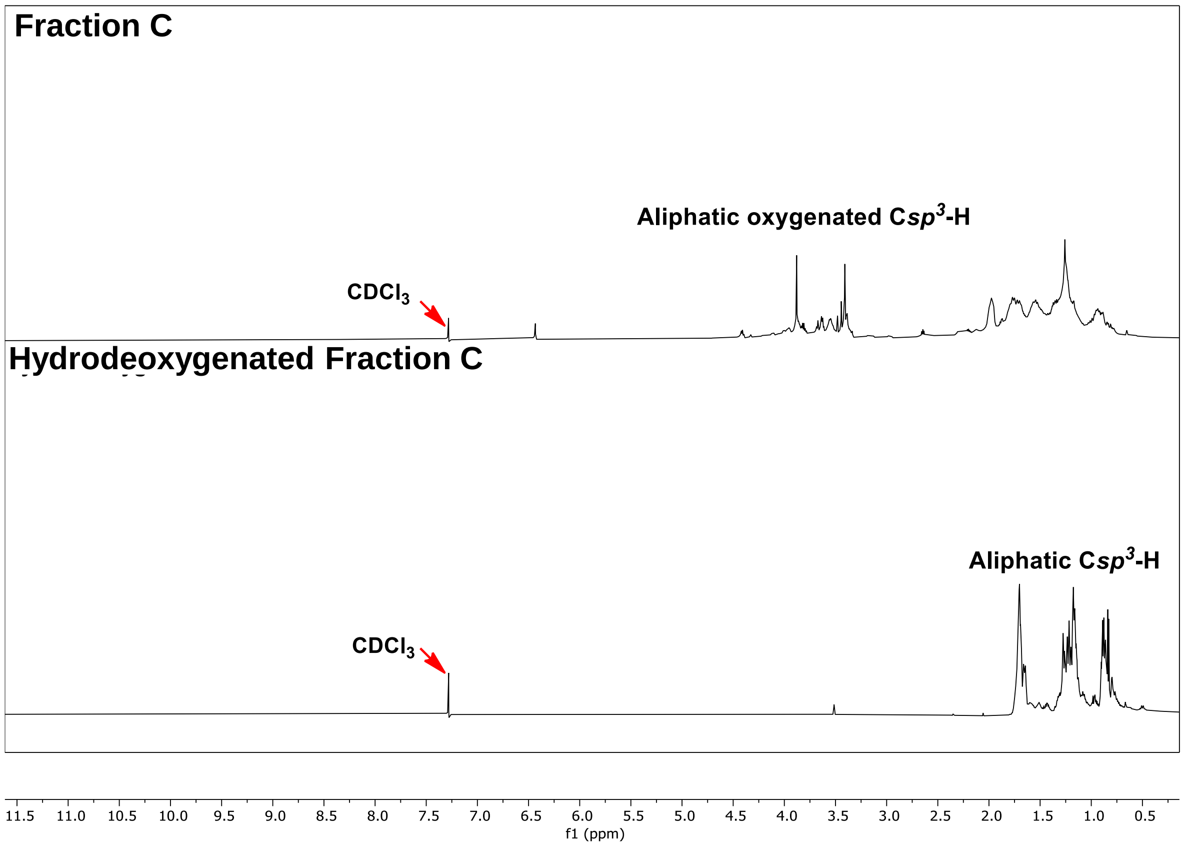

Supplementary Figure 83. ^{1}H NMR spectra of Fraction C and hydrodeoxygenated Fraction C

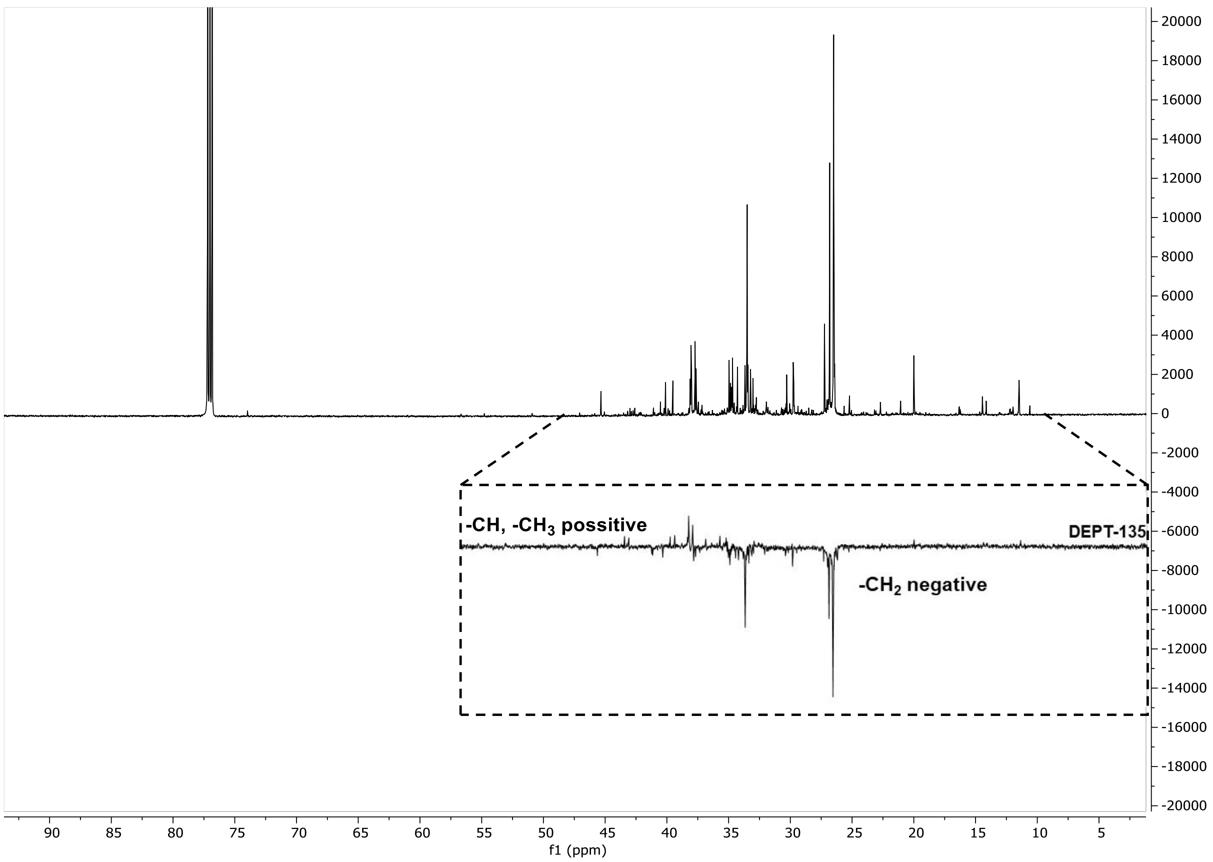

Supplementary Figure 84. ^{13}C NMR and DEPT 135 NMR spectra of hydrodeoxygenated Fraction C

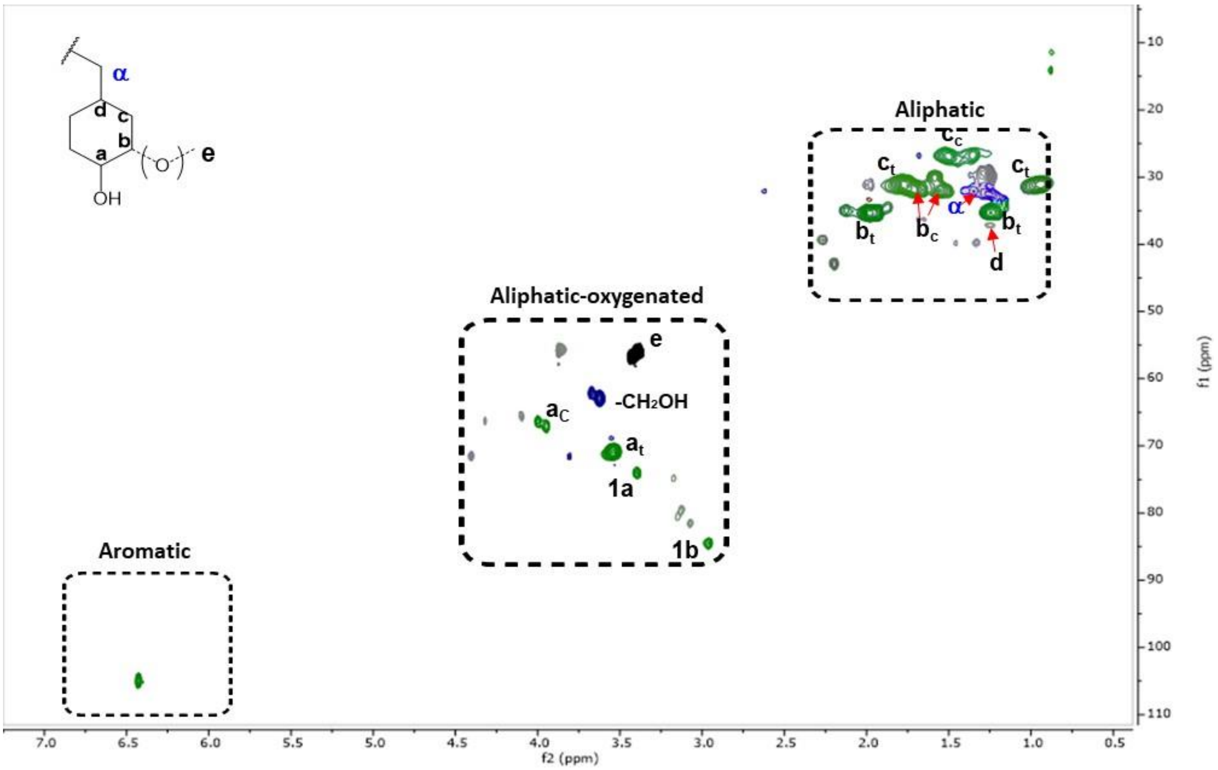

Supplementary Figure 85. 2D HSQC spectrum of Fraction C

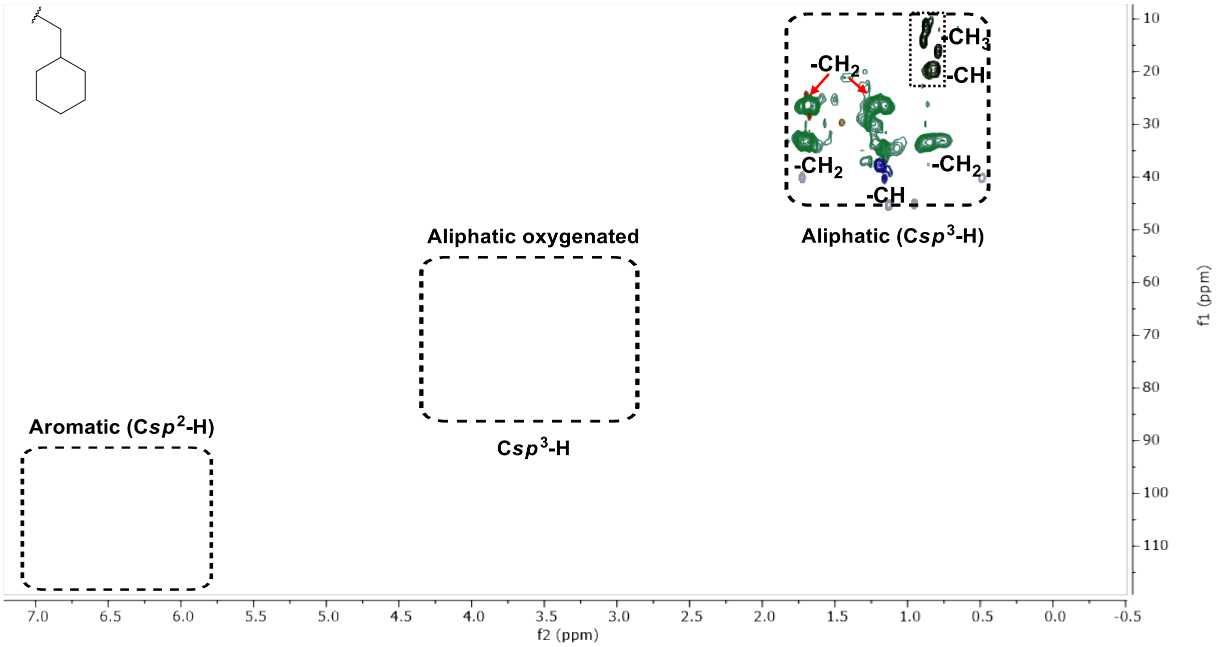

Supplementary Figure 86. 2D-HSQC spectrum of hydrodeoxygenated Fraction C[27]

3.6 Proposed catalytic downstream conversion pathways

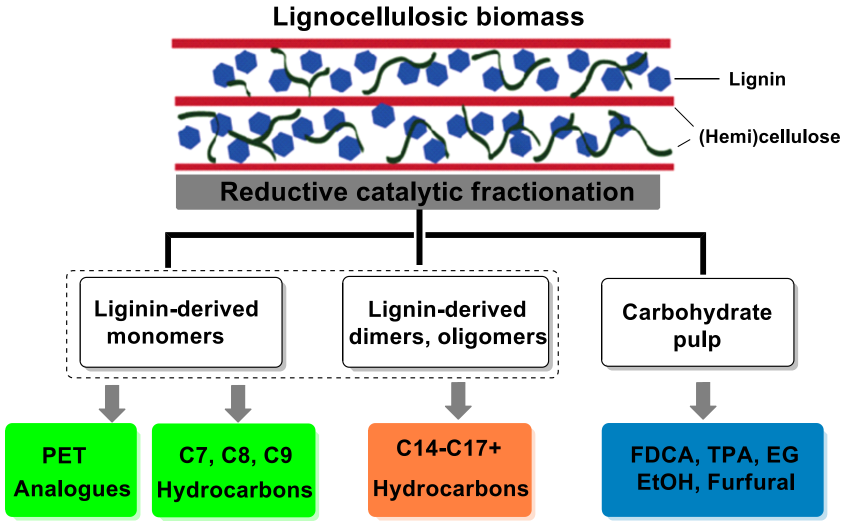

Supplementary Figure 87. Proposed downstream processing of lignocellulose to **PET** analogues. Native lignin is converted to **C7-C9** gasoline range hydrocarbons, **C14-C17** jet fuel range hydrocarbons and aliphatic diol **PC**. The carbohydrate pulp (mainly cellulose) can be transformed into **FDCA**, **TPA**, while the surplus can find applications towards the production of ethanol, ethylene glycol (**EG**) and furfural.

Supplementary Note 4. Recycling studies

4.1 Establishing of reaction conditions for alcoholysis of poly (PC/TPA)

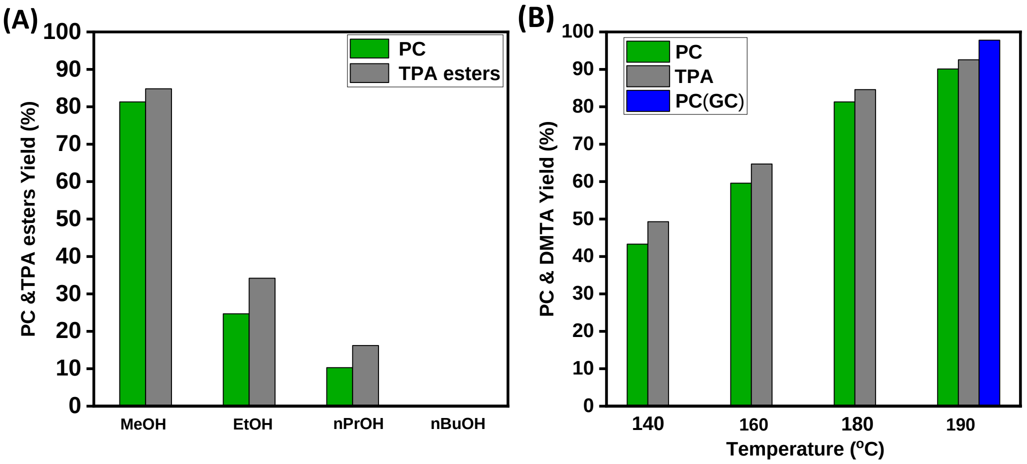

Supplementary Figure 88. Evaluating of reaction conditions for alcoholysis of poly (PC/TPA). a) 0.2 g poly (PC/TPA), 30 mL alcohols, 4h, 180 °C, isolated yield. b) GC yield in blue using dodecane as internal standand

4.2 A circular economy for production, recycling, reproduction of poly (PC/TPA)

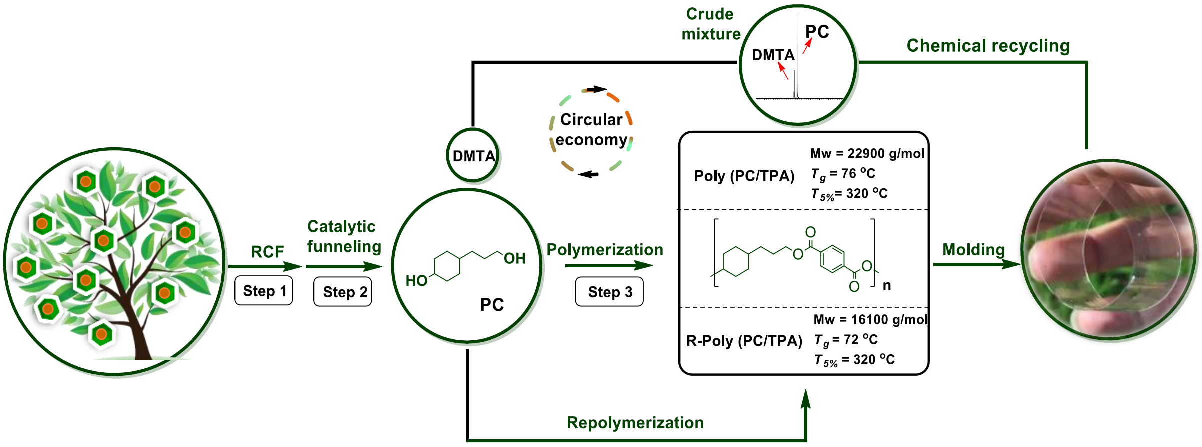

Supplementary Figure 89. A recycling strategy for poly (PC/TPA) recycling. Step 1: RCF of lignocellulose over previously developed Cu20-PMO catalyst; Step 2: Catalytic funneling of RCF crude mixture over Raney Ni catalyst using isopropanol as solvent.; Step 3: The co-polymerization of PC with DMTA to give the PET analogue poly (PC/TPA) over $Zn(OAc)_2$ catalyst; The recycling of monomers PC and DMTA via one-pot methanolysis of the poly (PC/TPA); The crude mixture from depolymerized poly (PC/TPA) was repolymerized over $Zn(OAc)_2$ catalyst to give R-poly (PC/TPA).

Depolymerization: The methanolysis depolymerization[27,28] reaction was carried out according to the General procedure D using previously obtained poly (PC/TPA) with Mw of 22910 g/mol, T_g of 76 °C and T_d at 5% of 320 °C. Typically, 0.7 g of poly (PC/TPA) and 30 mL methanol were placed in a 100 mL high pressure Parr autoclave and then the reactor was sealed. The reactor was heated to 190 °C for 4 h. After reaction was completed, the reactor was cooled to room temperature and the solvent was removed in vacuo. The obtained crude mixture was then analyzed by GC-FID (Supplementary Figure

90) and ^{1}H NMR (Supplementary Figure 91), which confirmed the presence of PC and DMTA. **Repolymerization:** The repolymerization of the crude mixture containing PC and DMTA was carried out according to General procedure D with extra 1 mol % $Zn(OAc)_2$ catalyst added. Finally, 0.32 g (32.8 % of yield) of poly (PC/TPA)-R was obtained. The GPC (Supplementary Figure 94), TGA (Supplementary Figure 93) and DSC (Supplementary Figure 92) characterizations showed that Mw of poly (PC/TPA)-R is 16120 mol/g and T_g is 72 °C and T_d at 5% mass loss is 320 °C.

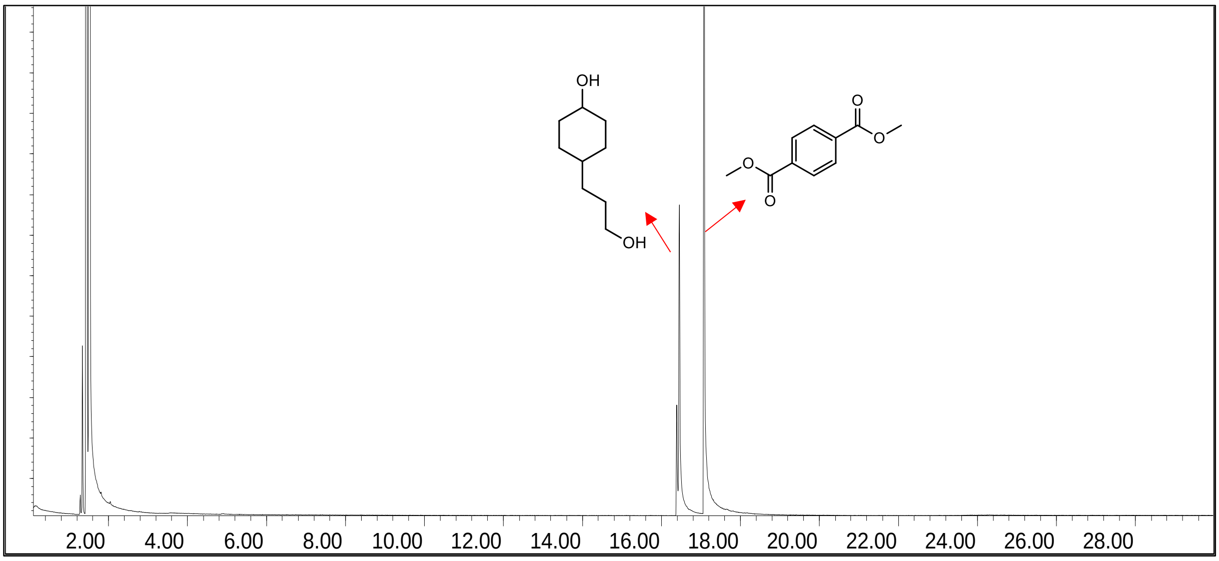

Supplementary Figure 90. GC-FID chromatogram of the crude mixture of poly (PC/TPA) depolymerization in methanol.

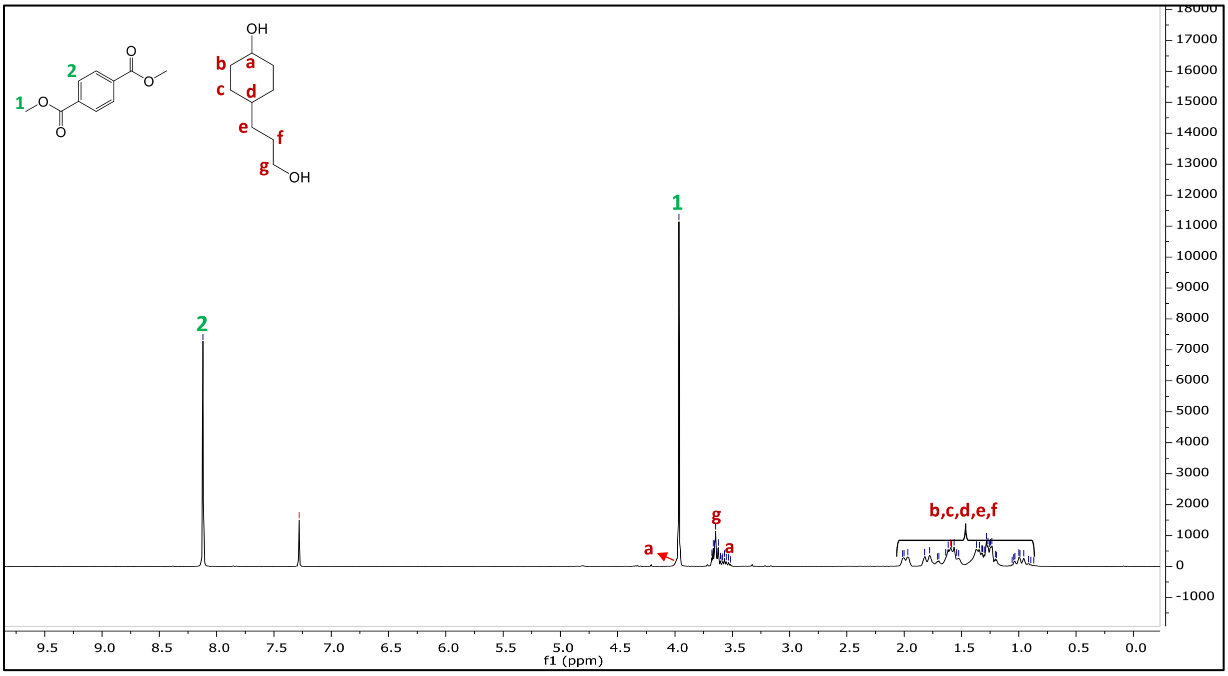

Supplementary Figure 91. ^{1}H NMR spectrum of the crude mixture of poly (PC/TPA) depolymerization.

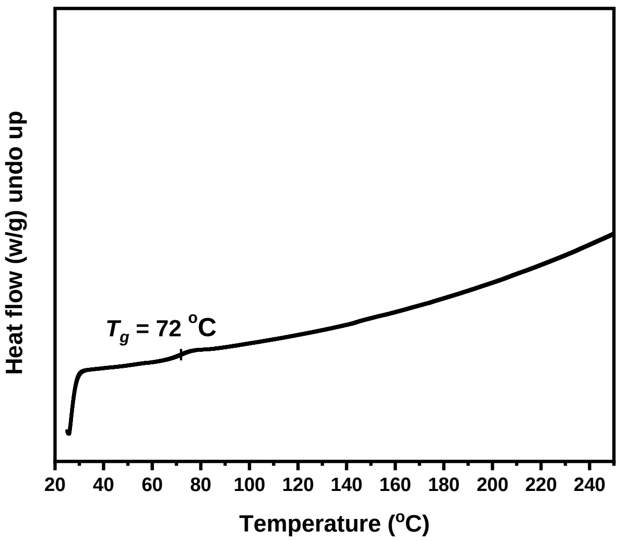

Supplementary Figure 92. DSC thermogram of repolymerized poly (PC/TPA)-R.

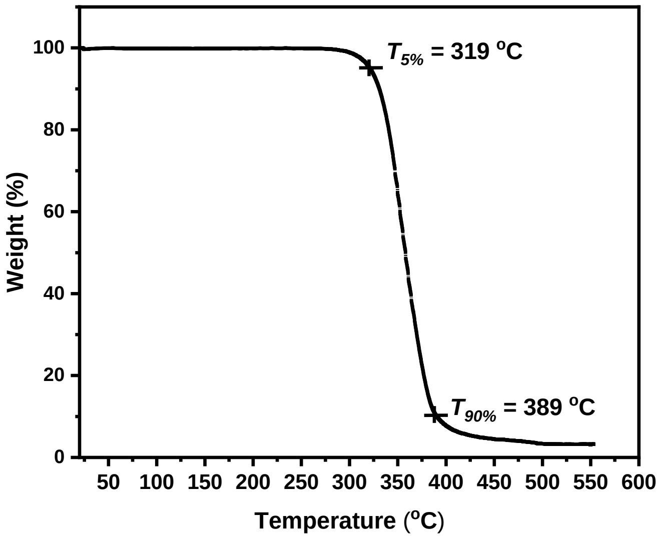

Supplementary Figure 93. TGA plot of repolymerized poly (PC/TPA)-R.

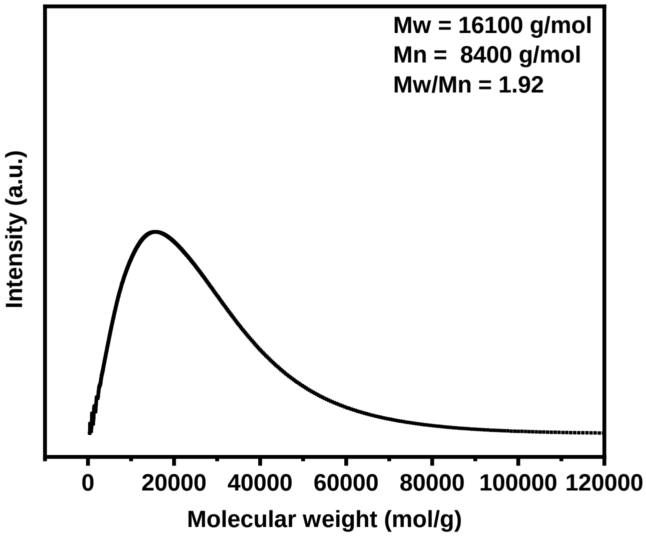

Supplementary Figure 94. GPC trace of repolymerized poly (PC/TPA)-R.

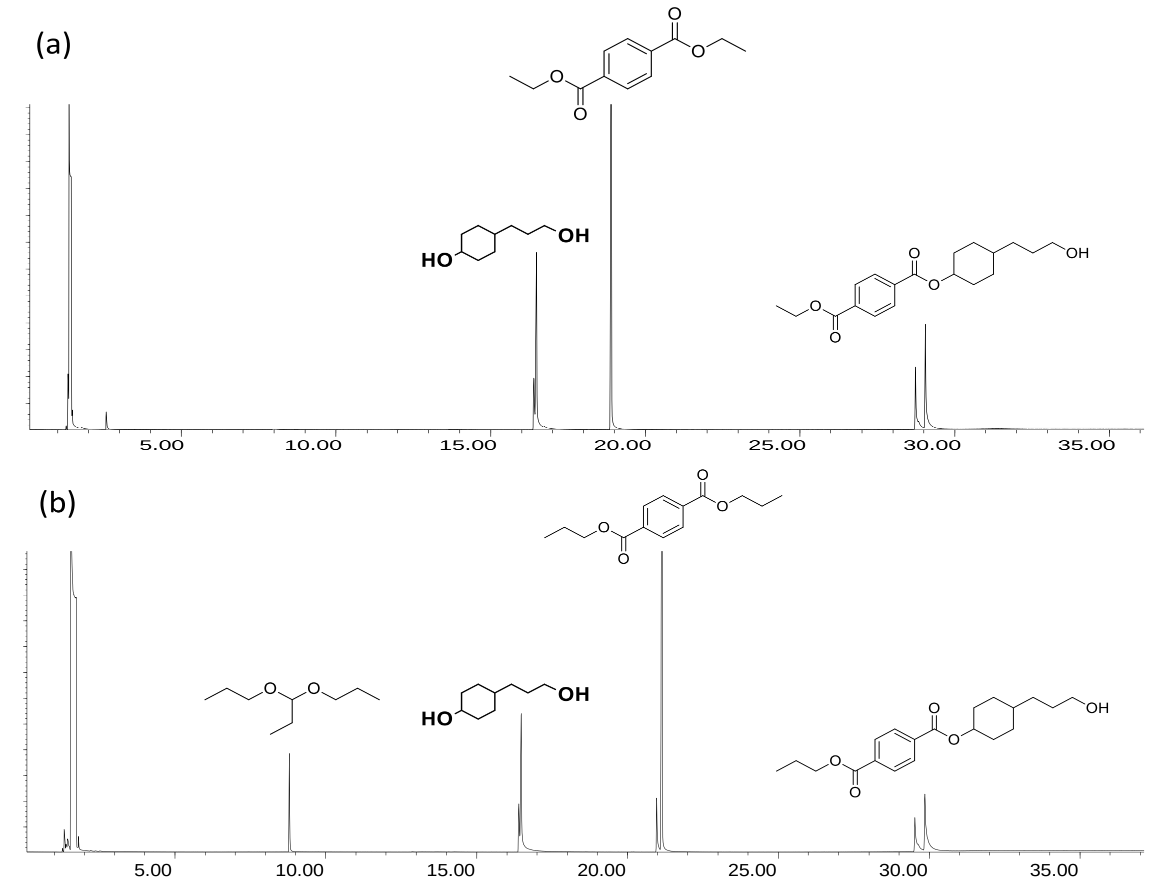

Supplementary Figure 95. GC traces of the crude depolymerization mixture of poly (PC/TPA) in a) ethanol; b) propanol.

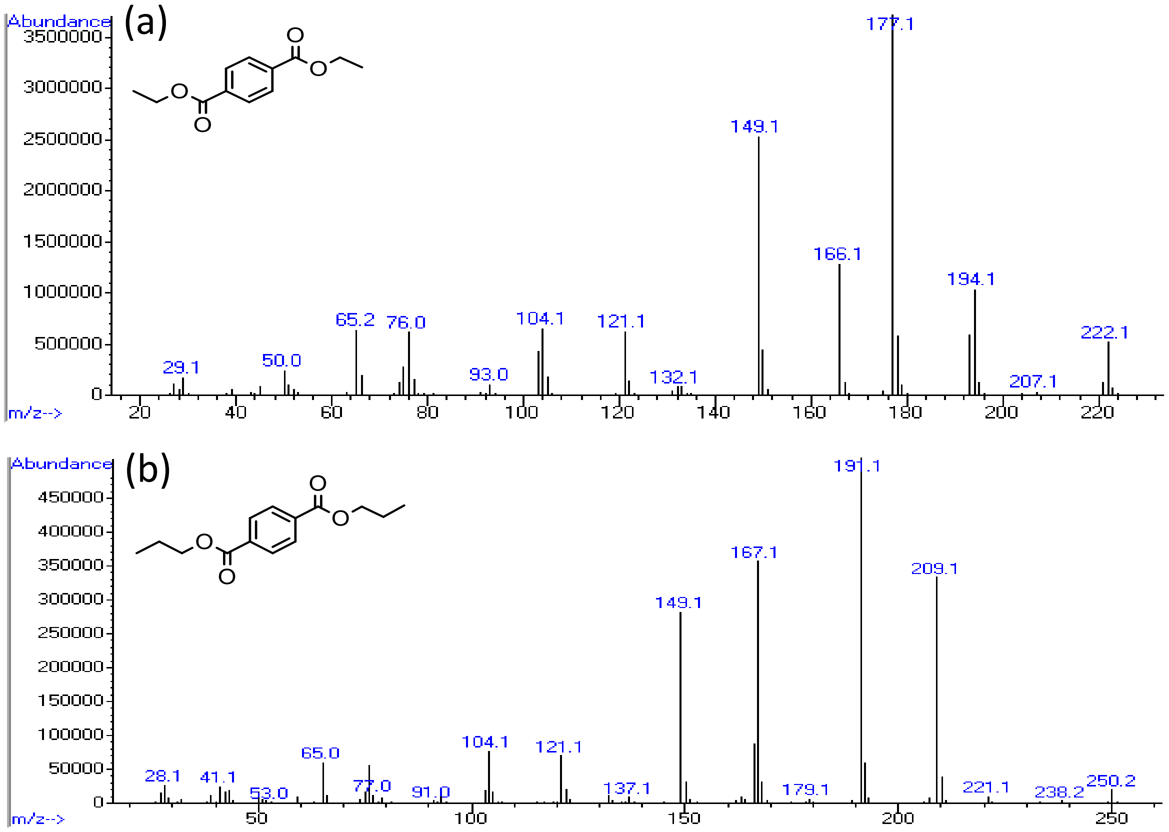

Supplementary Figure 96. Mass spectra of c) diethyl terephthalate and d) dipropyl terephthalate.

ICP analysis

To quantify Zn or Ti content in poly (PC/TPA) and poly (PC/TPA) after polymerization, elemental analysis was performed by ICP characterization. The results show that Zn concentration in poly (PC/TPA) is 2850 ppm (2850 mg/kg), while Ti concentration in poly (PC/TPA) is 1110 ppm (1110 mg/kg).

Supplementary Note 5. Considerations for the catalytic activity of Raney Ni catalyst in the demethoxylation /hydrogenation of 1G

Raney Ni is a highly active transfer hydrogenation catalyst in combination with isopropanol solvent for the demethoxylation prior to hydrogenation of guaiayl/syringyl phenolics, as summarized in excellent works of Rinaldi, Sun and our previous work[29,30,31]. The other noble metal catalysts possess a higher affinity for aromatic ring hydrogenation, able to produce methoxy-substituted cyclohexane ring. Further demethoxylation from the aliphatic moiety was much slower as supported by the mechanistic analysis has demonstrated in Fig. 2E,F

Supplementary Note 6. Determination of the lignin utilization efficiency based on a per carbon basis

According to the elementary analysis of lignin in beech wood, the elemental compositions for beech lignin is C (59.99 %), H (5.537 %), O (33.717 %) N (0.22 %) S (0.536 %). Thus, the total input mass of carbon is 1880×0.599 = 1128 mg.

To facilitate the analysis and calculation, we first assume that the formulae unindentified alkanes is C_nH_{2n} (7)

For Fraction A (158 mg): 50 mg Fraction A gave 3.2 mg C7, 9.5 mg C8 and 17.9 mg C9 and 1.8 mg other alkanes. The whole Fraction A should give 8.6 mg C7, 25.7 mg C8, 48.4 mg C9 and 4.9 mg other alkanes. Thus, the total mass of carbon is 87.6 mg = 8.6 + 25.7 + 48.4 + 4.9 mg.

For Fraction B (288 mg): PC (216 mg) and 1 (72 mg) were obtained. Thus, the total mass of carbon is 193.4 mg = 147.4 + 46.0 mg.

For Fraction C (162 mg): 50 mg Fraction C gave 1.2 mg $H_{\beta\text{-}1}$, 2.3 mg, $H_{\beta\text{-}5}$, 5.3 mg $H_{\beta\text{-}\beta}$, 0.8 mg $H_{5\text{-}5}$, 3.1 mg unrecogonised dimers and 5.9 mg oligomeric alkanes. The whole Fraction C should give 3.9 mg $H_{\beta\text{-}1}$, 7.5 mg, $H_{\beta\text{-}5}$, 17.1 mg $H_{\beta\text{-}\beta}$, 2.6 mg $H_{5\text{-}5}$, 10.0 mg unrecogonised dimers and 19.2 mg oligomeric alkanes. The total mass of carbon is 52 mg = 3.4 + 6.5 + 14.8 + 2.2 + 8.6 + 16.5 mg.

Thus, the total mass of carbon obtained from three Fractions is 333 mg = 87.6 + 193.4 + 52 mg

Thus, the total yield of carbon is: 29.5 wt % = 333/1128×100 %.

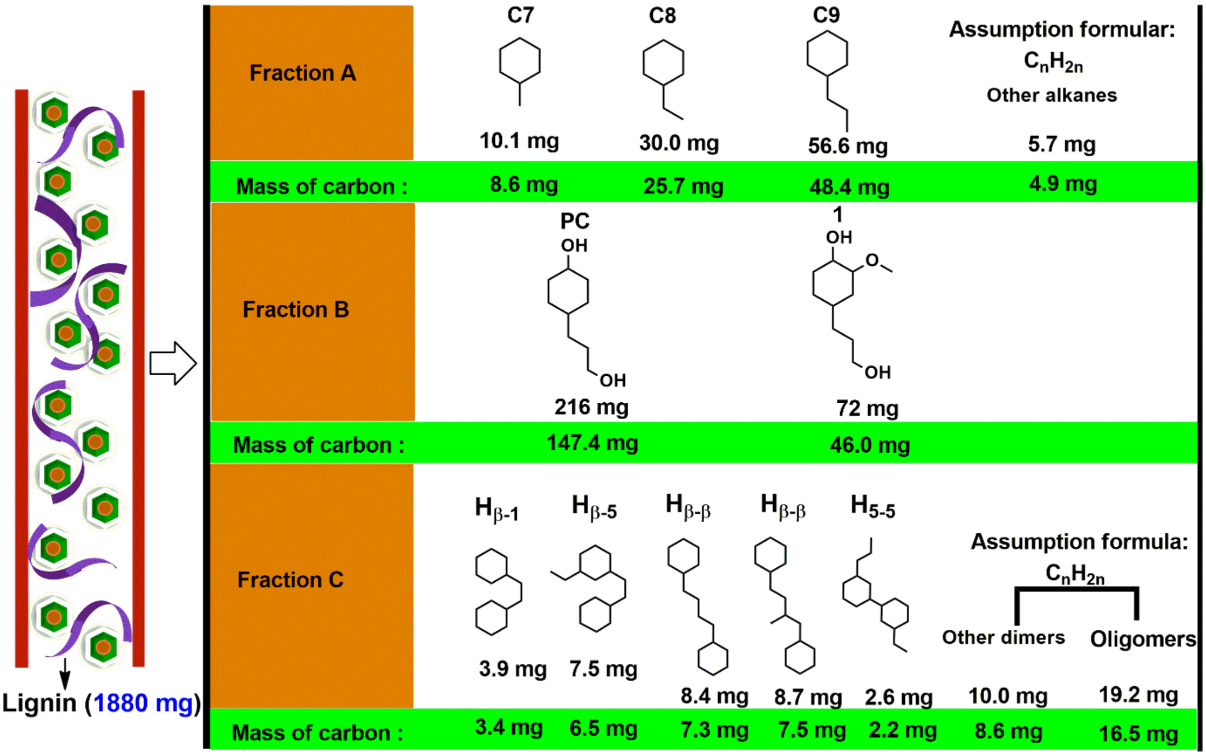

Supplementary Figure 97. Determination of the lignin utilization efficiency based on a per carbon basis

Supplementary Note 7. Establishment of pathway toward separation and conversion of carbohydrate to FDCA

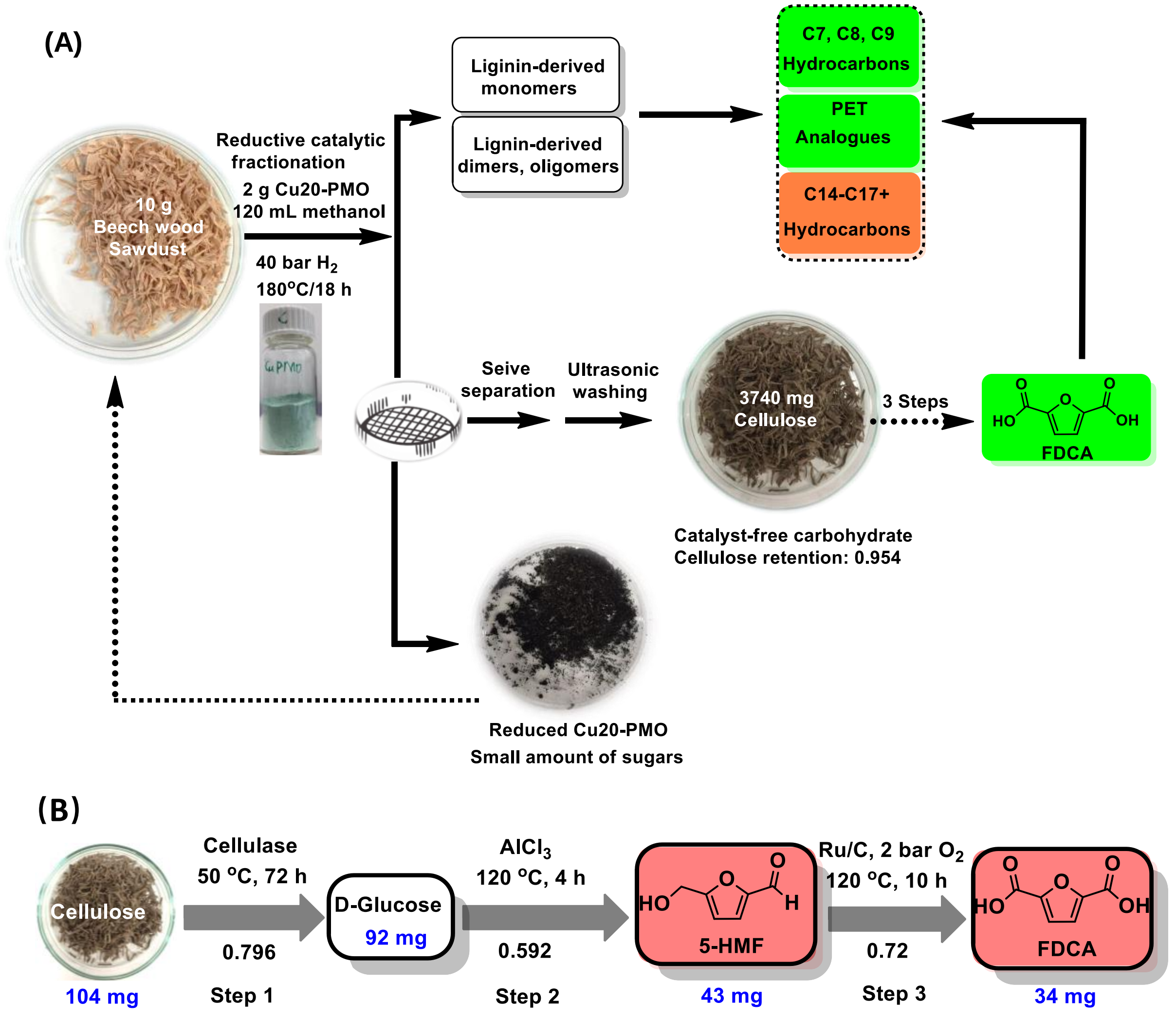

Supplementary Figure 98. A straightforward and effective protocol using sieve fractionation methodology to liberate the spent catalyst from carbohydrate pulp after **RCF** through a mesh screening; B) A catalytic reaction route for catalytic conversion of cellulose to **FDCA.**

In this work, we have first developed a straightforward protocol using beech wood sawdust with a size of more than 1 mm, taking advantage of sieve fractionation to liberate the spent Cu20-PMO catalyst from the carbohydrate pulp after RCF (Supplementary Figure 98). Typically, after RCF of beech wood, the spent Cu20-PMO catalyst was separated from the carbohydrate pulp through a mesh screening. Then, the solids were subjected to further ultrasonic treatment in water to get rid of the catalyst residues. After applying this method, the isolated reaction solids (mainly cellulose) were subjected to ICP analysis which showed minimal Cu contamination (1.45 mg Cu/ g carbohydrate) that confirmed the removal of Cu20-PMO catalyst. Next, the isolated carbohydrate fraction was subjected to three independent reaction steps (Step 1: Mild enzymatic hydrolysis of cellulose pulp to D-glucose; Step 2: Isomerization and dehydration of D-glucose to 5-HMF; Step 3: Catalytic oxidation of 5-HMF to FDCA) to result in FDCA in a mass yield of 32.7 wt% on a cellulose basis. The experimental details are listed as follow:

Step 1: Mild enzymatic hydrolysis of solid residue

The mild enzymatic hydrolysis of solid residue (0.2 g) (104 mg cellulose) was performed using cellulase (Cellic CTec2) (3 FPU) at sodium acetate buffer (10 mL, 5 mM, pH 4.8) in a glass vial equipped with a magnetic stirrer. The mixture was heated to 50 °C under 150 rpm for 72 h. After reaction, the solid residue was separated by filtration, the filtrate was collected and then analyzed by HPLC. Finally, 92 mg D-glucose was obtained in a mol yield of 79.6 %.

Step 2: Catalytic conversion of glucose to 5-HMF

The obtained filtrate was first evaporated and then 10 mL DMSO and $AlCl_3$ (0.02 g) was added. The reaction was proceeded in a round bottom flask at 120 °C for 4h. After reaction, $AlCl_3$ catalyst was removed by filtration and the filtrate was subjected to HPLC analysis, confirming 43 mg HMF was achieved in a mol yield of 59.2 %. HPLC equipped with C18 column 3.5 μm, 4.6 x 100 mm and UV detector was used for analysis and it was done in a mixture of methanol in water (5/95, v/v) as a solvent gradient at elution flow rate of 1 mL/min. The column temperature was 303 K and the injection volume of the sample (filtered through 0.22 μm membrane) was 20 mL.

Step 3: Catalytic oxidation of 5-HMF to FDCA

Catalytic conversion of 5-HMF to FDCA was carried out according to the reported literature[32], typically, a 50 ml Parr reactor was charged with 5-HMF (43 mg), H_2O (10 mL), Ru/C catalyst (5 wt %, 34 mg). The reactor was purged with O_2 thrice and pressurized with 2 bar O_2. The reactor was then heated to 120 °C in heating block, with stirring speed of 500 rpm. After 10 h, the reaction was cooled down to room temperature and the white solid FDCA and Ru/C were separated by filtration. Then, the FDCA was resolublized in methanol (10 mL) under sonication. After filtration and wash twice with methanol, the white solid was obtained after removing the solvent under reduced pressure. Isolated yield is 72 %.

Supplementary Note 8. Preliminary techno-economic analysis (TEA)

On the basis of the experimental data, we performed the techno-economic assessment (TEA) of the process. The comprehensive evaluation includes the catalytic processing of beech lignocellulose by RCF, followed by the fractionation of the obtained bio-oil and the catalytic processing of the respective fractions to final products. This includes the Raney Ni mediated catalytic funneling of the monomers to PC diol, which is then converted to the respective fully bio-based polyesters; as well as the separation and conversion of the carbohydrate rich residues to FDCA, as well as furfural (based on best literature).

Due to the lab-scale level of the current process development the assessment was based on the experimental inputs and outputs. The required raw material inputs and catalysts were normalized to one dry ton of beech wood processed. Specific cost unit values were taken from other papers and industrial sources. Solvent recovery and catalyst recycling were estimated based on other papers. Basic assumptions have been made in line with the literatures[33,34]. Thus, we estimated fixed operating costs, utility costs and annualized capital cost as a relative share based on raw material costs[35]. Supplementary Table 12 provides an overview on the most important raw material costs. Beech wood as the main feedstock of the process is also the costliest raw material in process.

Supplementary Table 12, Raw materials cost assumptions

Entry	Inputs	cost per ton (Euro)	Normalized cost per ton of processed dry-wood (Euro)	Ref.
1	Beech wood	59	73.78	36
2	EtOAc	977	35.27	37
3	Cyclohexane	860	26.81	38
4	Isopropanol	1422	22.36	39
5	Methanol	233	6.42	40
6	Other imputs	-	19.27	-
7	Raney Ni	31.85	9.83	40
8	HZSM-5	230.49	13.88	41
9	Ru/C	81.6	72.25	42
10	Cu20-PMO[a]	-	12.30	-
11	Other catalysts	-	0.04	-

[a]. Due to a lack of commercial availability, the cost of the Cu20-PMO catalyst was estimated based on the raw material costs. All catalysts were assumed to be sufficiently recyclable or maintainable (50% of the cost to 70% of the performance).

Supplementary Table 13, Products revenue assumptions

Entry	Outputs	price per ton (Euro)	Normalized revenue per ton of processed dry wood (Euro)	Ref.
1	Gasoline	62	0.60	43
2	PET	1275	51.76	44
3	Furfural	1190	125.42	45
4	FDCA	2635	256.38	45-47
5	Jet fuel	613	3.70	48
6	Methanol	233	9.96	40

Supplementary Table 14, Predicted CO_2 emissions

Entry	Outputs	CO_2-emissions based on ECOINVENT data (kg/kg)
1	Gasoline	0.602
2	PET	2.938
3	Jet fuel	0.447
4	Methanol	0.361

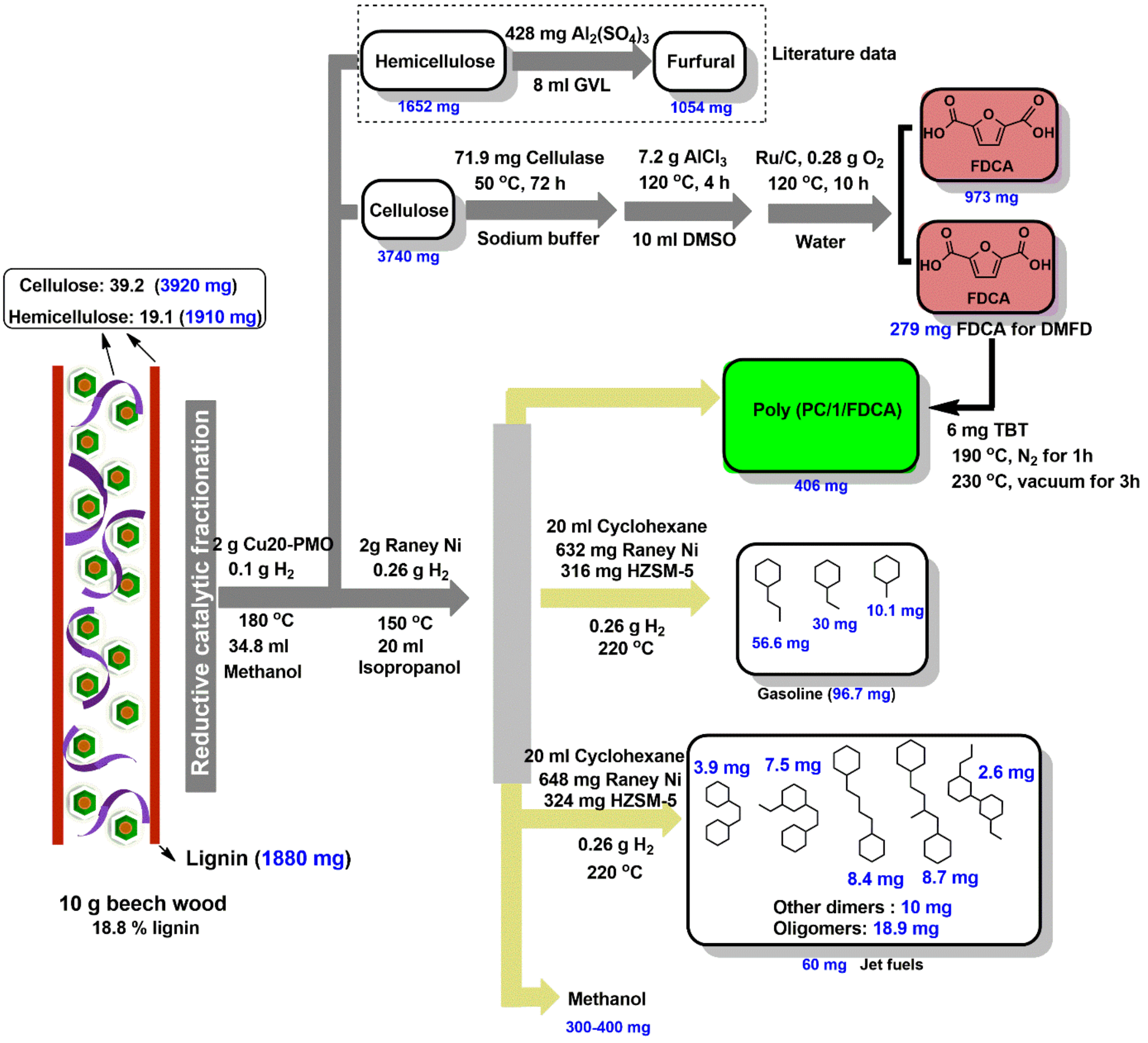

Supplementary Figure 99. Inputs and outputs for catalytic conversion of beech wood to chemicals, PET and jet fuels. The substrate to solvent ratios have to be optimized at lab-scale reaction.

Overall the process (shown in Supplementary Figure 99) converts beech wood into 1% of gasoline, 1% of jet fuel, 4% of PET and 4 % methanol, 11% of furfural and 10 % of FDCA on a mass basis (this being good efficiency with 80% of lignocellulose converted and deoxygenation taken place).

It is very encouraging, that with the *currently achieved product yields*, and with the assumptions made in line with literature data, the techno–economic evaluation shows a positive balance. More specifically, a 6.4 % rate of return can be achieved at 99 % solvent recovery. Returns are sensitive to methanol and

isopropanol recovery at 96 % and 98 % respectively. Overall, our analysis indicates that catalyst and solvent costs are the main drivers of operating costs, which is not surprising considering the lab-scale development stage of the process.

FDCA and furfural form the most important revenue streams (see Supplementary Table 13) while fuels are neglectable in both volume and value. Hence, the profitability of the process is particularly depending on future FDCA price assumptions. Since no mature FDCA market is yet existing. Thus the revenue values estimated in previous papers have been used[45,46,47].

It is clear that up-scaling would include a significant reduction of the solvent demand and consumption while optimizing its recovery. Another factor that future optimization may improve, is the total process yield, which is currently at 30 wt %. While this yield is already high considering the well-defined product streams obtained, we still see possibilities for improvement. For example, while the 12 wt % yield of PC is among the best in available literature for a lignin-based polymer building block, this value can be improved by optimizing the catalyst type and flow/vs batch operation of the RCF to maximize 1G/1S yield. For example, one of the highest yields to 1G/1S mixture in the literature is 44.8 wt %[49], compared to 24.2 wt % in this work. Consequently, the PC yield (22.2 wt %) could achieve about double the amount (12 wt %) currently observed. In addition, the yield value to **FDCA** can also be dramatically optimized by selecting the best catalytic system. For example, starting from the cellulose, a combined yield of 82 % to **FDCA** can be obtained based on two-step metal catalytic sequence [$RuCl_3$ catalyzed hydrolysis, dehydration and isomerization [50] and Pd/HT mediated catalytic oxidation [51] The amount of **FDCA** produced by this methodology can not only cover the required amount for the production of DMFD in a best yield of 99 %[52], but also provides the huge surplus **FDCA**. In fact, such an increase in yields would enable a profitable (7% return) operation of the process even under the assumption of the lowest possible FDCA prices discussed in literature[43].

Another important aspect is to carefully assess other benefits of bio-based products compared to fossil-based ones, especially in relation to carbon-neutrality and climate benefits. Our process is utilizing a relatively cheap raw material (Beech wood) and targets well defined and already existing products. However, current prices of the substituted fossil-based products are too low considering they are made from rather cheap bulk petrochemicals. However, when assuming emission pricing in the range of 50-100 Euros per ton CO_2 released would add between 2 and 4 % to the overall profitability.

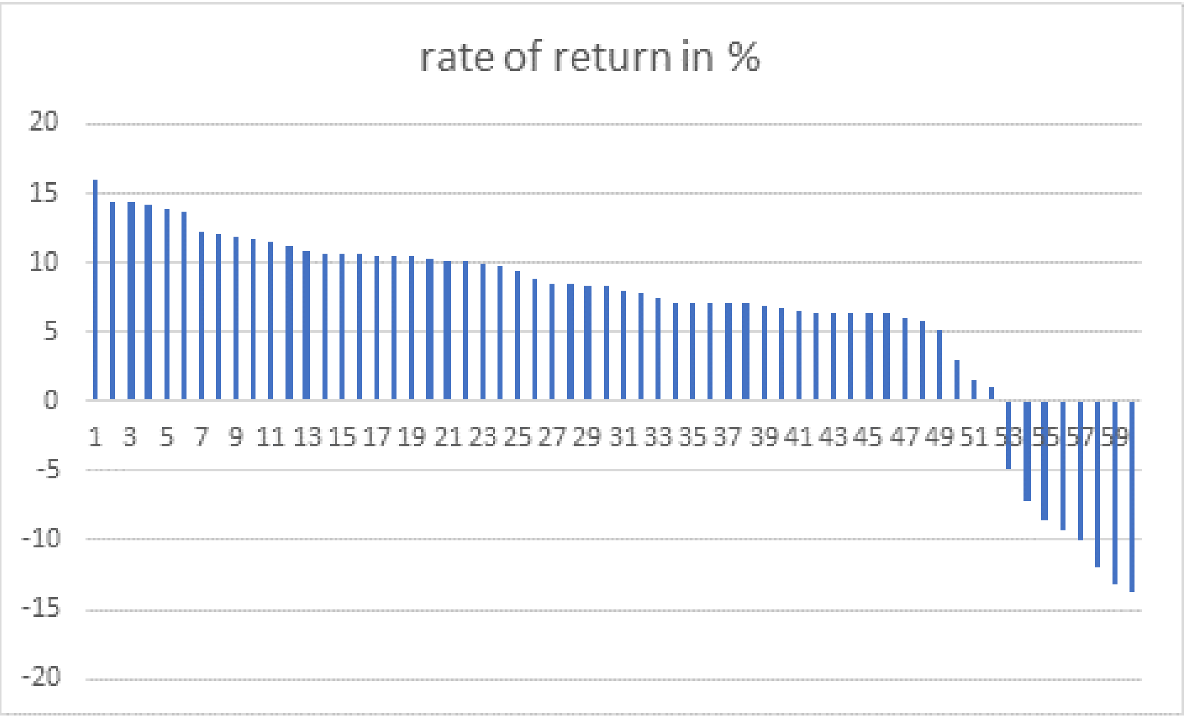

Supplementary Figure 100. Rate of return as result of 60 different scenarios when varying raw material costs, chemical costs, recovery rates, CO_2 prices, product prices, catalyst costs, always considering two different yield levels and with and without CO_2- taxation..

It has to be noted, that we performed the process economics analysis based on currently obtainable experimental data and available product price data cited from the literature and commercial webpages. At the current stage of development this assessment is considered as a prospective, order of magnitude estimate, typical for TRL 3 to 4[53]. The calculations were performed in Excel, and this approach has been proven successful in recent literature[54-56]. The **Supplementary Figure 100** above shows the results of 60 different scenarios when varying raw material costs, chemical costs, recovery rates, CO_2 prices, product prices, catalyst costs, always considering two different yield levels and with and without CO_2-taxation. The results turn negative in case of lower recovery and lower furfural prices. If these scenarios can be avoided we can see that most runs (scenarios) provide rates of return between 5 and 13 %.

Supplementary Note 9. Spectral data of isolated compounds

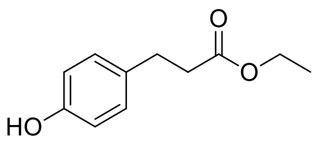

Para-coumaric acid ethyl ester, yield: 95 %. ^{1}H NMR (600 MHz, MeOD): δ 7.62 (d, *J* = 15.9 Hz, 1H), 7.50 – 7.44 (m, 2H), 6.85 – 6.79 (m, 2H), 6.33 (d, *J* = 15.9 Hz, 1H), 4.24 (q, *J* = 7.1 Hz, 2H), 1.33 (t, *J* = 7.1 Hz, 3H). ^{13}C NMR (151 MHz, MeOD): δ 167.92, 159.83, 144.94, 129.71, 125.79, 115.43, 113.97, 60.01, 48.04, 47.90, 47.76, 47.62, 47.47, 47.33, 47.19, 13.24.

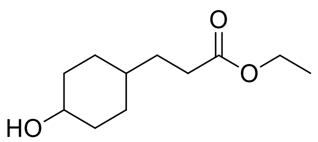

Ethyl 3-(4-hydroxycyclohexyl)propanoate, yield: 93 %. Cis-isomer: ^{1}H NMR (600 MHz, $CDCl_3$): δ 4.12 (q, J = 7.1 Hz, 2H), 3.98 – 3.94 (m, 1H), 2.34 – 2.27 (m, 2H), 1.74 – 1.67 (m, 2H), 1.62 – 1.47 (m, 6H), 1.43 – 1.29 (m, 4H), 1.25 (t, J = 7.1 Hz, 3H). ^{13}C NMR (151 MHz, $CDCl_3$): δ 174.02, 66.89, 60.23, 35.89, 32.19, 32.04, 31.08, 26.63, 14.25. Trans-isomer: ^{1}H NMR (600 MHz, $CDCl_3$): δ 4.06 (q, J = 7.1 Hz, 2H), 3.48 (tt, J = 10.9, 4.3 Hz, 1H), 2.26 – 2.21 (m, 2H), 1.94 – 1.87 (m, 2H), 1.74 – 1.67 (m, 2H), 1.50 – 1.35 (m, 4H), 1.22 – 1.10 (m, 6H), 0.91 (m, J = 13.3, 11.7, 3.3 Hz, 2H). ^{13}C NMR (151 MHz, $CDCl_3$): δ 174.1, 71.1, 60.4, 36.4, 35.6, 32.3, 31.7, 31.0, 14.4.

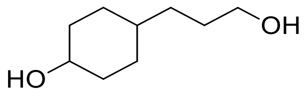

4-(3-Hydroxypropyl)cyclohexan-1-ol (**PC**): Cis-isomer, yield: 62.6 %. ^{1}H NMR (600 MHz, $CDCl_3$): δ 3.96 (dq, J = 5.0, 3.2, 2.7 Hz, 1H), 3.64 (td, J = 6.7, 1.3 Hz, 2H), 1.74 – 1.67 (m, 2H), 1.64 – 1.49 (m, 6H), 1.43 – 1.27 (m, 7H). ^{13}C NMR (151 MHz, $CDCl_3$): δ 67.3, 63.5, 36.3, 32.4, 32.2, 30.4, 27.1. *Trans*-isomer, yield: 67.6 %. ^{1}H NMR (600 MHz, $CDCl_3$): δ 3.62 (t, *J* = 6.6 Hz, 2H), 3.55 (m, *J* = 10.9, 4.3 Hz, 1H), 2.00 – 1.93 (m, 2H), 1.82 – 1.74 (m, 2H), 1.57 (m, *J* = 15.1, 6.8 Hz, 2H), 1.44 (s, 2H), 1.29 – 1.15 (m, 5H), 0.96 (m, *J* = 13.5, 11.1, 3.3 Hz, 2H). ^{13}C NMR (151 MHz, $CDCl_3$): δ 71.3, 63.4, 36.7, 35.7, 32.8, 31.4, 30.6.

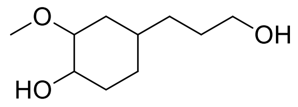

4-(3-hydroxypropyl)-2-methoxycyclohexanol, ^{1}H NMR (300 MHz, $CDCl_3$) δ 3.60 (t, *J* = 6.6 Hz, 2H), 3.43 – 3.29 (m, 4H), 2.95 (m, *J* = 11.2, 8.8, 4.3 Hz, 1H), 2.12 (m, *J* = 12.6, 5.1, 2.6 Hz, 1H), 2.01 – 1.93 (m, 1H), 1.71 (dp, *J* = 13.0, 3.1 Hz, 2H), 1.60 – 1.50 (m, 2H), 1.34 – 1.21 (m, 4H), 1.05 – 0.70 (m, 2H). ^{13}C NMR (75 MHz, $CDCl_3$) δ 84.61, 73.95, 62.98, 56.41, 35.82, 34.83, 32.55, 31.31, 30.43, 30.24.

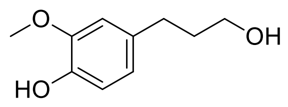

4-(3-Hydroxypropyl)-2-methoxyphenol (**1G**), yield: 68.9 %. ^{1}H NMR (600 MHz, $CDCl_3$): δ 6.83 (d, *J* = 7.8 Hz, 1H), 6.72 – 6.67 (m, 2H), 3.88 (s, 3H), 3.68 (t, *J* = 6.4 Hz, 2H), 2.65 (dd, *J* = 8.6, 6.4 Hz, 2H), 1.91 – 1.83 (m, 2H). ^{13}C NMR (151 MHz, $CDCl_3$): δ 146.5, 143.9, 133.9, 121.1, 114.4, 111.1, 62.5, 56.0, 34.7, 31.9.

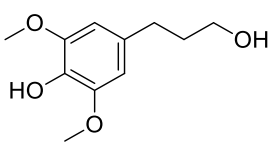

4-(3-Hydroxypropyl)-2,6-dimethoxyphenol (**1S**), yield: 63.2 %. ^{1}H NMR (600 MHz, $CDCl_3$): δ 6.43 (s, 2H), 3.88 (s, 6H), 3.69 (t, *J* = 6.4 Hz, 2H), 2.67 – 2.62 (m, 2H), 1.92 - 1.84 (m, 2H). ^{13}C NMR (151MHz, $CDCl_3$): δ 149.7, 135.6, 135.5, 107.7, 64.7, 58.9, 37.1, 34.9.

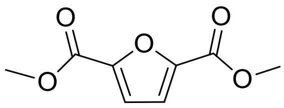

Dimethyl 2,5-furamdicarboxylate (**DMFD**), yield: 81 %. ^{1}H NMR (600 MHz, $CDCl_3$): δ 7.15 (d, *J* = 1.0 Hz, 2H), 3.87 (d, *J* = 1.1 Hz, 6H). ^{13}C NMR (151 MHz, $CDCl_3$): δ 158.44, 146.68, 118.50, 52.43.

Supplementary Note 10. DynaFit script and input data

```
[task]
task = fit
data = progress discontinuous
[mechanism]
1g ---> 1h : k1
1g ---> 1 : k3
1g ---> 2 : k5
1h ---> 2 : k6
1h ---> pc : k2
1 ---> 2 : k7
1 ---> pc : k4

[constants]
k1 = 0.1 ?
k2 = 0.1 ?
k3 = 0.1 ?
k4 = 0.1 ?
k5 = 0.1 ?
k6 = 0.1 ?
k7 = 0.1 ?
[concentrations]
1g = 100
[data]
directory ./flow/1gmin103
sheet data.csv
column 2 | response 2 = 1 | label 2
column 3 | response pc = 1 | label pc
column 4 | response 1h = 1 | label 1h
column 5 | response 1 = 1 | label 1
column 6 | response 1g = 1 | label 1g

[output]
directory ./flow/1gmin103
[settings]
{Output}
```

XAxisLabel = time, min103

YAxisLabel = concentration, %

[end]

col 01, col 02, col 03, col 04, col 05, col 06

"t, min103",   "2,%", " pc,%",   "1h,%",   "1

Supplementary references

1. Sun, Z. H., Bottari, G., Afanasenko, A., Stuart, M. C. A., Deuss, P. J., Fridrich, B. & Barta, K. Complete lignocellulose conversion with integrated catalyst recycling yielding valuable aromatics and fuels. *Nat. Catal.* **1**, 82-92 (2018).
2. Elangovan, S., Afanasenko, A., Haupenthal, J., Sun, Z. H., Liu, Y. Z., Hirsch, A. K. H. & Barta, K. From Wood to Tetrahydro-2-benzazepines in Three Waste-Free Steps: Modular Synthesis of Biologically Active Lignin-Derived Scaffolds. *ACS Cent. Sci.* **5**, 1707-1716 (2019).
3. Doyle, M. P., Protopopova, M. N., Zhou, Q.-L., Bode, J. W., Simonsen, S. H. & Lynch, V. Optimization of enantiocontrol for carbon-hydrogen insertion with chiral dirhodium (II) carboxamidates. Synthesis of natural dibenzylbutyrolactone lignans from 3-aryl-1-propyl diazoacetates in high optical purity. *J. Org. Chem.* **60**, 6654-6655 (1995).
4. Itagaki, N., Kimura, M., Sugahara, T. & Iwabuchi, Y. Organocatalytic entry to chiral bicyclo [3. n. 1] alkanones via direct asymmetric intramolecular aldolization. *Org. Lett.* **7**, 4185-4188 (2005).
5. Gubbels, E., Jasinska-Walc, L. & Koning, C. E. Synthesis and characterization of novel renewable polyesters based on 2, 5-furandicarboxylic acid and 2, 3-butanediol. *J. Polym. Sci. Poly. Chem.* **51**, 890-898 (2013).
6. Scanlon, J. T. & Willis, D. E. Calculation of flame ionization detector relative response factors using the effective carbon number concept. *J. Chromatogr. Sci.* **23**, 333-340 (1985).
7. Solanki, B. S. & Rode, C. Selective hydrogenolysis of 5-(hydroxymethyl) furfural over Pd/C catalyst to 2, 5-dimethylfuran. *J. Saudi. Chem. Soc.* **23**, 439-451 (2019).
8. Chang, C.-C., Green, S. K., Williams, C. L., Dauenhauer, P. J. & Fan, W. Ultra-selective cycloaddition of dimethylfuran for renewable p-xylene with H-BEA. *Green Chem.* **16**, 585-588 (2014).
9. Iglesias, J., Martínez-Salazar, I., Maireles-Torres, P., Alonso, D. M., Mariscal, R. & Granados, M. L. Advances in catalytic routes for the production of carboxylic acids from biomass: a step forward for sustainable polymers. *Chem. Soc. Rev.* **49**, 5704-5771 (2020).
10. Kalsi, D., Barsu, N. & Sundararaju, B. CoIII-Catalyzed Isonitrile Insertion/Acyl Group Migration Between C− H and N− H bonds of Arylamides. *Chem. Eur. J.* **24**, 2360-2364 (2018).
11. Miles-Barrett, D. M., Neal, A. R., Hand, C., Montgomery, J. R., Panovic, I., Ojo, O. S., Lancefield, C. S., Cordes, D. B., Slawin, A. M. & Lebl, T. The synthesis and analysis of lignin-bound Hibbert ketone structures in technical lignins. *Org. Biomol. Chem.* **14**, 10023-10030 (2016).
12. Sun, J. K., Li, H. L., Huang, H. Z., Wang, B., Xiao, L. P. & Song, G. Y. Integration of Enzymatic and Heterogeneous Catalysis for One-Pot Production of Fructose from Glucose. *ChemSusChem* **11**, 1157-1162 (2018).
13. Schell, D. J., Dowe, N., Chapeaux, A., Nelson, R. S. & Jennings, E. W. Accounting for all sugars produced during integrated production of ethanol from lignocellulosic biomass. *Bioresource Technol.* **205**, 153-158 (2016).

14. Wang, A. Q. & Zhang, T. One-pot conversion of cellulose to ethylene glycol with multifunctional tungsten-based catalysts. *Acc. Chem. Res.* **46**, 1377-1386 (2013).
15. Yang, T., Zhou, Y. H., Zhu, S. Z., Pan, H. & Huang, Y. B. Insight into aluminum sulfate-catalyzed xylan conversion into furfural in a γ-valerolactone/water biphasic solvent under microwave conditions. *ChemSusChem* **10**, 4066-4079 (2017).
16. Song, S., Zhang, J., Gözaydın, G. & Yan, N. Production of terephthalic acid from corn stover lignin. *Angew. Chem. Int. Ed.* **58**, 4934-4937 (2019).
17. Kuzmič, P. Program DYNAFIT for the analysis of enzyme kinetic data: application to HIV proteinase. *Ana. Biochem.* **237**, 260-273 (1996).
18. Mialon, L., Pemba, A. G. & Miller, S. A. Biorenewable polyethylene terephthalate mimics derived from lignin and acetic acid. *Green Chem.* **12**, 1704-1706 (2010).
19. Scifinder, https://scifinder.cas.org/scifinder/view/scifinder/scifinderExplore.jsf Search C-NMR for 1,4-Benzenedicarboxylic acid, 1,4-dicyclohexyl ester (CAS Registry Number 18699-51-9)
20. Nishii, Y., Hirai, T., Fernandez, S., Knochel, P. & Mashima, K. Zinc-Catalyzed Esterification of N-β-Hydroxyethylamides: Removal of Directing Groups under Mild Conditions. *Eur. J. Org. Chem.* **2017**, 5010-5014 (2017).
21. Luo, W. K., Jiang, K., Li, Y. W., Jiang, H. F. & Yin, B. L. Direct alkoxycarbonylation of heteroarenes via Cu-mediated trichloromethylation and in situ alcoholysis. *Org. Lett.* **22**, 2093-2098 (2020).
22. Taguchi, Y., Oishi, A. & Iida, H. One-step synthesis of dibutyl furandicarboxylates from galactaric acid. *Chem. Lett.* **37**, 50-51 (2008).
23. Ioakeimidis, C., Fotopoulou, K., Karapanagioti, H., Geraga, M., Zeri, C., Papathanassiou, E., Galgani, F. & Papatheodorou, G. The degradation potential of PET bottles in the marine environment: An ATR-FTIR based approach. *Sci. Rep.* **6**, 1-8 (2016).
24. Yu, Z. L., Zhou, J. D., Cao, F., Zhang, Q. G., Huang, K. X. & Wei, P. Synthesis, characterization and thermal properties of bio-based poly (ethylene 2, 5-furan dicarboxylate). *J. Macromol. Sci. B* **55**, 1135-1145 (2016).
25. Schutyser, W., Van den Bosch, S., Renders, T., De Boe, T., Koelewijn, S.-F., Dewaele, A., Ennaert, T., Verkinderen, O., Goderis, B. & Courtin, C. Influence of bio-based solvents on the catalytic reductive fractionation of birch wood. *Green Chem.* **17**, 5035-5045 (2015).
26. Van den Bosch, S., Schutyser, W., Vanholme, R., Driessen, T., Koelewijn, S.-F., Renders, T., De Meester, B., Huijgen, W., Dehaen, W. & Courtin, C. Reductive lignocellulose fractionation into soluble lignin-derived phenolic monomers and dimers and processable carbohydrate pulps. *Energ. Environ. Sci.* **8**, 1748-1763 (2015).
27. Wang, H. L., Ruan, H., Pei, H. S., Wang, H. M., Chen, X. W., Tucker, M. P., Cort, J. R. & Yang, B. Biomass-derived lignin to jet fuel range hydrocarbons via aqueous phase hydrodeoxygenation. *Green Chem.* **17**, 5131-5135 (2015).

28. Tang, H., Li, N., Li, G. Y., Wang, A. Q., Cong, Y., Xu, G. T. A., Wang, X. D. & Zhang, T. Synthesis of gasoline and jet fuel range cycloalkanes and aromatics from poly (ethylene terephthalate) waste. *Green Chem.* **21**, 2709-2719 (2019).
29. Wang, X. Y. & Rinaldi, R. Exploiting H-transfer reactions with RANEY (R) Ni for upgrade of phenolic and aromatic biorefinery feeds under unusual, low-severity conditions. *Energ. Environ. Sci*. **5**, 8244-8260 (2012).
30. Wu, X. Y., Galkin, M. V., & Barta, K. A well-defined diamine from lignin depolymerization mixtures for constructing bio-based polybenzoxazines. *Chem Catal.*, **1**, 1466-1479 (2021).
31. Sun, Z. H., Zhang, Z. H., Yuan, T. Q., Ren, X. H., Rong, Z. M., Raney Ni as a Versatile Catalyst for Biomass Conversion. *ACS Catal*. **11**, 10508-10536 (2021).
32. Yi, G. S., Teong, S. P. & Zhang, Y. G. Base-free conversion of 5-hydroxymethylfurfural to 2,5-furandicarboxylic acid over a Ru/C catalyst. *Green Chem*. **18**, 979-983 (2016).
33. Liao, Y. H., Koelewijn, S. F., Van den Bossche, G., Van Aelst, J., Van den Bosch, S., Renders, T., Navare, K., Nicolai, T., Van Aelst, K., Maesen, M., Matsushima, H., Thevelein, J. M., Van Acker, K., Lagrain, B., Verboekend, D. & Sels, B. F. A sustainable wood biorefinery for low–carbon footprint chemicals production. *Science* **367**, 1385-1390 (2020).
34. Couper, J. R., Hertz, D. W. & Smith, F. L. Process economics, Perry's Chemical Engineering's Handbook, Section 9, pp. 1-56 (1997).
35. Lee, Y., Park, H., Han, J. & Lee, J. Economically-feasible production of a nylon monomer using RANEY catalysts. *React. Chem. Eng.* **6**, 225-234 (2021).
36. https://www.lwk-niedersachsen.de/lwk/news/30508_Holzpreise_Privatwald_Niedersachsen
37. https://www.intratec.us/chemical-markets/ethyl-acetate-price
38. Li, J., Peng, Z. J., Li, C. S., Li, P. & Gani, R. Process design and economic analysis of methacrylic acid extraction for three organic solvents. *Chinese J. Chem. Eng.* **27**, 2909-2916 (2019).
39. https://www.echemi.com/productsInformation/pid_Rock3921-isopropanol.html
40. Bartling, A. W., Stone, M. L., Hanes, R. J., Bhatt, A., Zhang, Y. M., Biddy, M. J., Davis, R., Kruger, J. S., Thornburg, N. E., Luterbacher, J. S., Rinaldi, R., Samec, J. S. M., Sels, B. F., Roman-Leshkov, Y. & Beckham, G. T. Techno-economic analysis and life cycle assessment of a biorefinery utilizing reductive catalytic fractionation. *Energ. Environ. Sci.* **14**, 4147-4168 (2021).
41. Shemfe, M., Gu, S. & Fidalgo, B. Techno-economic analysis of biofuel production via bio-oil zeolite upgrading: An evaluation of two catalyst regeneration systems. *Biomass Bioenerg*. **98**, 182-193 (2017).
42. https://m.made-in-china.com/product/Ruthenium-Carbon-Ruthenium-Carbon-Catalyst-5-Ru-Can-Customize-10-Ruthenium-Carbon-Palladium-Carbon-837138905.html
43. https://www.indexmundi.com/commodities/?commodity=gasoline
44. https://www.spglobal.com/platts/en/market-insights/latest-news/petrochemicals/110421-european-virgin-pet-rises-above-rpet-as-virgin-pet-prices-hit-10-year-high

45. Dubbink, G. H. C., Geverink, T. R. J., Haar, B., Koets, H. W., Kumar, A., van den Berg, H., van der Ham, A. G. J. & Lange, J. P. Furfural to FDCA: systematic process design and techno-economic evaluation. *Biofuel Bioprod. Bior*. **15**, 1021-1030 (2021).
46. Triebl, C., Nikolakis, V., & Ierapetritou, M. Simulation and economic analysis of 5-hydroxymethyl-furfural conversion to 2, 5-furandicarboxylic acid. *Comput. Chem. Eng*. **52**, 26-34 (2013).
47. Dessbesell, L., Souzanchi, S., Rao, K. T. V., Carrillo, A. A., Bekker, D., Hall, K. A., Lawrence, K. M., Tait, C. L. J. & Xu, C. B. Production of 2,5-furandicarboxylic acid (FDCA) from starch, glucose, or high-fructose corn syrup: techno-economic analysis. *Biofuel Bioprod. Bior.* **13**, 1234-1245 (2019).
48. https://www.iata.org/en/publications/economics/fuel-monitor/
49. Chen, X., Zhang, K., Xiao, L. P., Sun, R. C., & Song, G. Total utilization of lignin and carbohydrates in Eucalyptus grandis: an integrated biorefinery strategy towards phenolics, levulinic acid, and furfural. *Biotechnol. biofuels*, **13**, 1-10 (2020).
50. Yan, L. S., Ma, R. S., Wei, H. X., Li, L. Z., Zou, B. & Xu, Y. W. Ruthenium trichloride catalyzed conversion of cellulose into 5-hydroxymethylfurfural in biphasic system. *Bioresource Technol.* **279**, 84-91 (2019).
51. Wang, Y. B., Yu, K., Lei, D., Si, W., Feng, Y. J., Lou, L. L. & Liu, S. X. Basicity-Tuned Hydrotalcite-Supported Pd Catalysts for Aerobic Oxidation of 5-Hydroxymethyl-2-furfural under Mild Conditions. *ACS Sustainable Chem. Eng.* **4**, 4752-4761 (2016).
52. Qiao, F. L., Hou, Y. B., Qin, B., Jiang, J. L., Li, C. F., Gao, M., Yu, W. F., Quaternary ammonium salt surfactant, preparation method and applications thereof, Patent, CN111072511 (2018).
53. Van Dael, M., Marquez, N., Reumerman, P., Pelkmans, L., Kuppens, T., & Van Passel, S. Development and techno-economic evaluation of a biorefinery based on biomass (waste) streams - case study in the Netherlands. *Biofuel Bioprod. Bior*. **8**, 635-644 (2014).
54. Tan, J. B., Jamali, N. S., Tan, W. E., Man, H. C. & Abidin, Z. Z. Techno-Economic Assessment of On-Farm Anaerobic Digestion System Using Attached-Biofilm Reactor in the Dairy Industry. *Sustainability,* **13**, 2063 (2021).
55. Thomassen, G., Vila, U. E., Van Dael, M., Lemmens, B. & Van Passel, S. A techno-economic assessment of an algal-based biorefinery. *Clean Technol. Envir.* **18**, 1849-1862 (2016).
56. Gogar, R., Viamajala, S., Relue, P. A. & Varanasi, S. Techno-Economic Assessment of Mixed-Furan Production from Diverse Biomass Hydrolysates. *ACS Sustainable Chem. Eng.* **9**, 3428-3438 (2021).
